# Fibroblasts from the Human Skin Dermo-Hypodermal Junction are Distinct from Dermal Papillary and Reticular Fibroblasts and from Mesenchymal Stem Cells and Exhibit a Specific Molecular Profile Related to Extracellular Matrix Organization and Modeling

**DOI:** 10.3390/cells9020368

**Published:** 2020-02-05

**Authors:** Valérie Haydont, Véronique Neiveyans, Philippe Perez, Élodie Busson, Jean-Jacques Lataillade, Daniel Asselineau, Nicolas O. Fortunel

**Affiliations:** 1 Advanced Research, L’Oréal Research and Innovation, 93600 Aulnay-sous-Bois, France; veronique.neiveyans@rd.loreal.com (V.N.); philippe.perez@rd.loreal.com (P.P.); daniel.asselineau@rd.loreal.com (D.A.); 2 Department of Medical and Surgical Assistance to the Armed Forces, French Forces Biomedical Research Institute (IRBA), 91223 CEDEX Brétigny sur Orge, France; elodie.busson.eb@gmail.com (É.B.); jjlataillade@gmail.com (J.-J.L.); 3 Laboratoire de Génomique et Radiobiologie de la Kératinopoïèse, Institut de Biologie François Jacob, CEA/DRF/IRCM, 91000 Evry, France; 4 INSERM U967, 92260 Fontenay-aux-Roses, France; 5 Université Paris-Diderot, 75013 Paris 7, France; 6 Université Paris-Saclay, 78140 Paris 11, France

**Keywords:** human dermis, fibroblasts, extracellular matrix (ECM), dermo-hypodermal junction, papillary fibroblasts (Fp), reticular fibroblasts (Fr), Tenascin C (TNC), mesenchymal stem cells (MSCs)

## Abstract

Human skin dermis contains fibroblast subpopulations in which characterization is crucial due to their roles in extracellular matrix (ECM) biology. This study investigates the properties of fibroblasts localized at the frontier of deep dermis and hypodermis, i.e., dermo-hypodermal junction fibroblasts (F-DHJ), which were compared to intermediate reticular dermis (Fr) and superficial papillary dermis (Fp) fibroblasts. F-DHJ differed from Fr and Fp cells in their wider potential for differentiation into mesodermal lineages and in their absence of contractility when integrated in a three-dimensional dermal equivalent. The transcriptomic profile of F-DHJ exhibited specificities in the expression of genes involved in ECM synthesis-processing and “tissue skeleton” organization. In accordance with transcriptome data, ECM proteins, notably Tenascin C, distributions differed between the reticular dermis and the dermo-hypodermal junction areas, which was documented in normal adult skin. Finally, genome-wide transcriptome profiling was used to evaluate the molecular proximity of F-DHJ with the two dermal fibroblast populations (Fp and Fr) and with the mesenchymal stem cells (MSCs) corresponding to five tissue origins (bone marrow, fat, amnion, chorion, and cord). This comparative analysis classified the three skin fibroblast types, including F-DHJ, as a clearly distinct group from the five MSC sample origins.

## 1. Introduction

In human skin, interfollicular dermis is a heterogeneous tissue compartment, considering its fibroblast content and extracellular matrix (ECM) structure. Its segmentation into two biologically distinct territories (i.e., superficial papillary dermis and deeper reticular dermis) occurs during the embryonic development at 12 weeks of gestation in humans [1,2]. Major structural specificities of these dermal territories concern collagen reticulation and organization of the elastin network, which are dynamic characteristics in constant evolution during the intra-uterine and postnatal life (for review, see [3]). 

Specificities of the different dermal territories also concern their fibroblast contents, in which characterization drives an increasing interest considering their widely expected functions in skin physiology. The existence of two dermal fibroblast populations, named papillary (Fp) and reticular (Fr) fibroblasts according to their dermal localization, was reported in human skin in the late seventies [4]. Since then, studies have been conducted to further explore their cellular properties [5] and molecular profiles [6,7]. Biological aspects that are attracting attention are the cellular and molecular changes that affect Fp and Fr cells through skin ageing [8,9]. 

Other fibroblast or fibroblast-like mesenchymal cell populations are present within the dermis, such as pericytes and telocytes. Pericytes appear in the fetal dermis at eight weeks of gestation in humans and acquire their mature characteristics at 21 weeks of gestation [10]. These cells contribute to the maintenance of capillary vessel integrity and may play a role in the maintenance of mesenchymal tissues in the contexts of homeostasis and/or wound healing [11]. In addition, pericytes may contribute to the niche that regulates the symmetrical versus asymmetrical division choice of epidermal keratinocyte precursors [12]. Telocytes possess an atypical fibroblast morphology characterized by long and slender moniliform cellular prolongations termed telopodes [13]. These cells serve as connecting devices, constructing homocellular junctions and connections with other cells types [14]. Telocytes are usually present at a low density (around 10 cells/mm^2^) [15]. These cells may participate in the stem cell niche, as shown in the intestine crypts [16]. Another described function of telocytes is the transmission of signals via atypical junctions [17] or extracellular vesicles [18], as reported in the heart. In the dermis, telocyte density augments with depth, together with the quantity of telopodes found in connection with endothelial cells, nerve endings, and hair follicle bulges [19]. Implications of telocytes in regeneration and wound healing is expected in skin but not fully demonstrated [19]. 

In the present study, we investigated the cellular and molecular properties of fibroblasts localized at the frontier of deep dermis and hypodermis, i.e., dermo-hypodermal junction fibroblasts (F-DHJ). Using parameters such as contractility, differentiation potential, and the supportive effect on epidermis reconstruction, we documented marked functional differences between F-DHJ and dermal (Fp and Fr) fibroblasts. At a molecular level, the study identified specific signatures in F-DHJ concerning the expression of genes involved in ECM synthesis-processing and “tissue skeleton” organization, which could explain structural properties of their tissue compartment. Finally, genome-wide transcriptome profiling was used to evaluate the molecular proximity of F-DHJ with the two dermal fibroblast populations (Fp and Fr) and the with mesenchymal stem cells (MSCs) corresponding to five tissue origins (bone marrow, fat, amnion, chorion, and cord). This approach identified skin fibroblasts and MSCs as distinct groups and will certainly contribute to the knowledge of the hierarchical clustering within the mesenchymal lineages. 

## 2. Materials and Methods

### 2.1. Fibroblast Isolation and Culture

#### 2.1.1. Human Skin Biopsy Collection 

**Figure 1:**
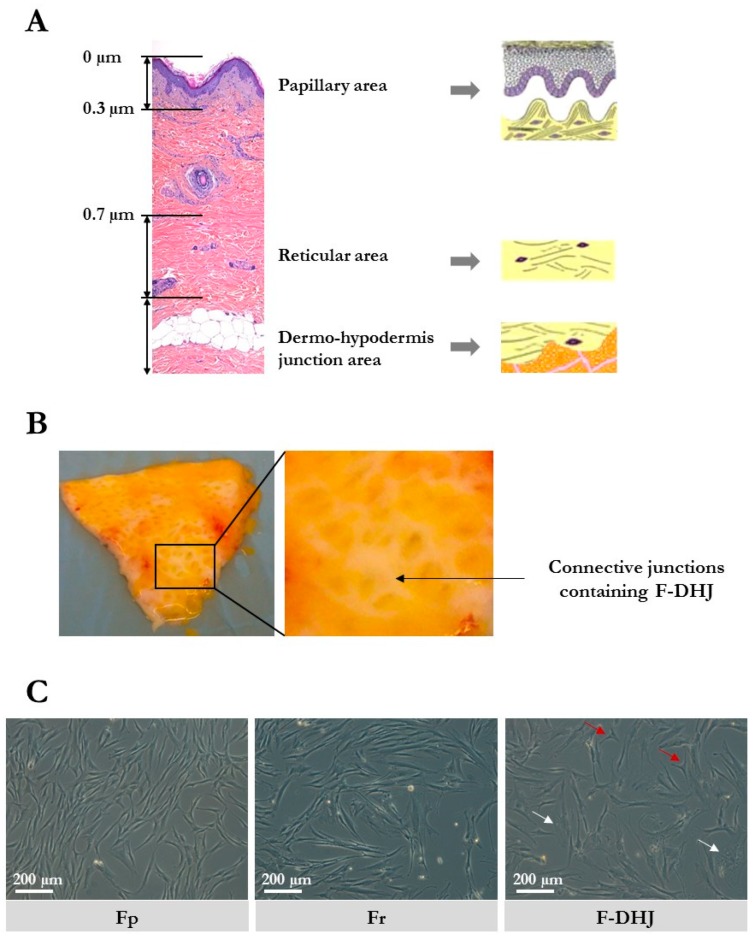
Skin localization and cellular morphology of papillary dermis fibroblasts (Fp), reticular dermis fibroblasts (Fr), and dermo-hypodermal junction (DHJ) fibroblasts. (**A**) Representation of the papillary dermis, reticular dermis, and dermo-hypodermis junction areas. A typical full-thickness skin section is shown, as well as schemes of the three areas of interest. (**B**) Photographs of skin pieces taken from the below side after fat tissue removal, showing the macroscopic aspect of the conjunctival junctions that connect the dermis to the hypodermis. (**C**) Cellular morphology of cultured Fp, Fr, and DHJ fibroblasts. In F-DHJ cultures, red arrows point to small tricuspid cells and white arrows to large cells with a visible trabecular cytoplasmic network.

Full-thickness biopsies of human breasts and abdominal skin, collected from healthy subjects undergoing reconstructive or aesthetical surgery, were obtained from Icelltis (Toulouse, France); Alphenyx (Marseille, France); and Biopredic (Saint-Grégoire, France) under the authorizations delivered by the French Ministry of Research with the approval of the French Ethical Committee. The written informed consent was obtained from all individuals. The tissue collection used in this study included 10 biopsies of breast skin (mammoplasties) with ages ranging between 18 and 65 years and 6 biopsies of abdominal skin (abdominoplasties) with ages between 42 and 51 years. A typical skin section showing the papillary, reticular, and dermo-hypodermal dermis regions is shown in Figure 1A.

F-DHJ hypodermis was gently removed from skin biopsies by dissection using clamps and scissors to preserve the junction between hypodermis and dermis. Then, the tissue area containing the conjunctival junctions that connect the dermis to the hypodermis (Figure 1B) was harvested by dissection for extraction of fibroblasts from the demo-hypodermal junction (DHJ). Dissected pieces were checked under binocular loupe and selected according to the presence of both adipose tissue and conjunctival structures, validating their DHJ localization. F-DHJ were then extracted by tissue digestion with type II collagenase 0.2% (Gibco, France) for 2 h at 37 °C. Tissue dissociation was facilitated by 30 s of vortexing every 30 min. 

#### 2.1.2. Fp and Fr 

After removing the epidermis by treatment with 2.4 U/mL dispase (Roche, Boulogne-Billancourt, France) for 16 h at 4°C and then mechanical dissection, papillary fibroblasts (Fp) were extracted by digestion of the tissue in type II collagenase 0.2% (Gibco, France) for 3 h at 37 °C. Tissue dissociation was facilitated by 30 s of vortexing every 30 min. Then, a second cut was performed on the noncut remaining part of the sample at a depth of 700 µm. This intermediate region of the dermis (depth between 300 and 700 µm) was not used for fibroblast extraction to avoid mixing papillary and reticular material. The deepest dermis slice (700 µm depth from skin surface and below) corresponded strictly to the reticular dermis and was used to extract the Fr fibroblast fraction by tissue digestion in type II collagenase 0.2% (Gibco, France) for 5 h at 37 °C. Tissue dissociation was facilitated by 30 s of vortexing every 30 min. 

#### 2.1.3. Bidimensional Mass Culture 

Fp, Fr, and F-DHJ cells were cultured in similar conditions. Seeding density was 3800 cells/cm², and culture medium was composed of MEM supplemented with 10% FBS (PAN Biotech GmbH, Aidenbach, Germany); penicillin-streptomycin (20 U/mL) (Biochrom Ltd., Cambridge, UK); sodium pyruvate (Gibco, France); nonessential amino acids (Gibco, France); and glutamine (2 mM) (Invitrogen, Carlsbad, CA, USA). Cultures were incubated at 37 °C in a 90% humidified atmosphere containing 5% CO_2_. 

### 2.2. Mesenchymal Stem Cell (MSC) Isolation and Culture 

All human samples were collected and handled in full respect of the Declaration of Helsinki.

#### 2.2.1. BM-MSCs

Human bone marrow MSCs (BM-MSCs) were obtained from patients undergoing routine total hip replacement surgery in Percy Hospital (Clamart, France) after written informed consent. As previously reported [20], spongious bone fragments were mixed in phosphate-buffered saline (PBS, PAN-Dominique Dutscher, Issy-les-Moulineaux, France); 1 mM EDTA (Prolabo-VWR, Paris, France); ACD-A; and 0.5% human serum-albumin (HAS, LFB). After 20 min of settling, the supernatant was collected, centrifuged at 480 *g* for 10 min, and filtered (70 µm). Bone marrow mononuclear cells (BM-MNCs) were counted using an automated cell analyzer (Sysmex, Villepinte, France)

#### 2.2.2. Ad-MSCs

Human adipose tissue MSCs (Ad-MSCs) were isolated from fat obtained after liposuction surgery in Percy Hospital (Clamart, France) after written informed consent. Fat was washed by an addition of PBS supplemented with 1 µg/mL ciprofloxacin (Panpharma, Luitré, France). After centrifugation at 815 *g* for 2 min, the washing solution (containing blood, lipids, and adrenalin added before surgery) was discarded. This operation was repeated until washing solution was clear. Fat tissue was then enzymatically digested in 0.075% type I collagenase (75 mg/100 mL fat) for 45 min at 37 °C with agitation each 15 min. Digested fat was then centrifuged at 200 *g* for 5 min. The supernatant that contained lipids and adipocytes was discarded. The pellet that contained the stoma-vascular fraction was washed three times with α-MEM (Cliniscience, Nanterre, France) and filtered (70 µm). Cell numeration was performed after sample treatment with Zap Oglobin lytic reagent (Beckman Coulter, Villepinte, France).

#### 2.2.3. Amnion, Chorion, and Umbilical Cord MSCs 

Perinatal tissues were obtained from full-term deliveries after maternal written informed consent (Hôpital d’Instruction des Armées Bégin, Saint-Mandé). As previously reported [20], samples of placental membranes (amnion and chorion) and umbilical cords were incubated in an antibiotic and antifungal solution for 90 min at room temperature and then cut into pieces. Amnion and chorion 2 cm^2^ pieces were digested in PBS containing 0.1% type IV collagenase (Thermo-Fisher for Life Technologies, Waltham, MA, USA) and 2.4 U/mL grade II dispase (Roche, Boulogne-Billancourt, France) for 90 min at 37 °C and then in PBS containing 0.025% trypsin-EDTA (Thermo-Fisher for Life Technologies, Waltham, MA, USA) for 30 min at 37 °C. Umbilical cord 2 cm-long pieces were cut into smaller formats (around 1–2 mm^3^) for digestion in PBS containing 300 U/mL type I collagenase (Thermo-Fisher for Life Technologies, Waltham, MA, USA) and 1 mg/mL hyaluronidase (Calbiochem-Merck, Fontenay sous Bois, France) for 60 min at 37 °C and then in PBS containing 0.025% trypsin-EDTA (Thermo-Fisher for Life Technologies, Waltham, MA, USA) for 30 min at 37 °C. Cell samples were filtered through a 100 µm cell strainer (BD Biosciences, Le Pont de Claix, France) and then centrifuged at 200 *g* for 10 min. Cells were counted in a Malassez chamber using the trypan blue exclusion method. 

#### 2.2.4. Bidimensional Mass Cultures 

Samples from the different tissue origins were cultured in the same conditions. Freshly-extracted cells were seeded at a density of 30000 cells/cm^2^ in a medium composed of α-MEM (Clinisciences, Nanterre, France) supplemented with 0.01 mg/mL ciprofloxacin; 2 U/mL heparin (Choay-Sanofi Aventis); and 5% platelet lysate (obtained from a platelet apheresis collection performed at the ‘Centre de Transfusion Sanguine des Armées’, Clamart). The medium was renewed 3 times a week. Cultures were trypsinized when reaching the stage of 80% confluence (trypsin-EDTA, Thermo-Fisher for Life Technologies, Waltham, MA, USA). Then, MSC subcultures were initiated at a density of 4,000 cells/cm^2^. For storage, MSC samples were frozen in α-MEM (Clinisciences, Nanterre, France) supplemented with 10% human serum-albumin and 10% DMSO (Sigma-Aldrich, St Louis, MO, USA). 

### 2.3. Colony Assay

Cells were plated at low densities in 10 cm diameter culture-treated plastic petri dishes (400 cells/dish for Fp and 800 cells/dish for Fr and F-DHJ) and cultured during 3 weeks in a medium of similar composition to that used for mass cultures, which was renewed 3 times a week. Cultures were then fixed in 70% ethanol and stained with blue RAL. Colonies were counted manually. 

### 2.4. Three-Dimensional Fibroblast Contractility Assay 

Dermal equivalents (lattices) were produced by mixing 100000 fibroblasts in MEM containing 10% FBS and 26% (*w*/*v*) bovine type I collagen (Symatèse, Chaponost, France) in a total volume of 5 mL (3.4 mm diameter petri dishes). Spontaneous collagen polymerization occurred within a few hours of culture. Lattices were then detached from the plastic surface of petri dishes 48 h after culture initiation, enabling a contraction process that led to progressive reduction of the lattice diameter. Kinetics of contraction was characterized by measurement of the lattice diameter (millimeter scale) after 1 h, 3 h, 6 h, and 24 h. Full description of the assay principle is provided in [8]. 

### 2.5. Three-Dimensional Skin Reconstruction 

Reconstructed skins were prepared as previously described [21]. Briefly, fibroblasts (1 × 10^6^ cells per sample of reconstructed dermis) were embedded into a bovine type I collagen gel (Symatese, Chaponost, France). Thereafter, keratinocytes (50,000 cells per sample) were seeded onto the lattices and stuck to the bottom of 60 mm diameter petri dishes. The keratinocytes used in this study were frozen banked samples from a single donor amplified in a serum-containing medium in the presence of a feeder-layer of growth-arrested murine 3T3 fibroblasts [5] according to the principle described by Rheinwald and Green [22]. Developing skin reconstructs were maintained for 1 week immersed in a medium composed of MEM (Invitrogen, Carlsbad, CA, USA) supplemented with 10% FBS (Sigma, St Louis, MO, USA); epidermal growth factor (EGF) (10 ng/mL) (BD Biosciences, San Jose, CA, USA); hydrocortisone (0.4 mg/mL) (Sigma, St Louis, MO, USA); and cholera toxin (0.1 nM) (Biomol Int., Plymouth, PA, USA). Complete epidermal stratification and full differentiation was obtained 1 week after raising the system at the air-liquid interface. During the whole process of skin reconstruction, cultures were maintained at 37 °C in a fully humidified atmosphere containing 5% CO_2_. Reconstructed skin samples were embedded in a paraffin and used to prepare hematoxylin eosin saffron-stained sections. 

### 2.6. Neosynthetized ECM Samples

Protocol was adapted from [23]. Fibroblasts were seeded on glass slides and cultured till postconfluence. After an additional 48 h, slides were washed twice in PBS, and cells were then lysed using a solution containing 0.5% Triton X-100 and 20 mM of NH_4_OH. Cell debris were washed in PBS. Slides coated with ECM components synthesized by fibroblasts were stored in PBS at 4 °C until characterization. 

### 2.7. Mesodermal Differentiation Assays

#### 2.7.1. Adipocyte Lineage 

Fibroblasts were seeded at a density of 1400 cells/cm^2^ and cultured in the medium used for mass expansion and colony assay till confluency. After an additional 48 h, the fibroblast cultures medium was substituted by an adipocyte differentiation medium composed of DMEM/20% fetal calf serum (PAN Biotech GmbH, Aidenbach, Germany); 60 µM indometacin (Dr. Ehrenstorfer GmbH, Germany); 0.5 mM 3-isobutyl-1-methylxanthine (IBMX) (Sigma, St Louis, MO, USA); and 10^−6^ M dexamethasone (Sigma, St Louis, MO, USA). After 3 weeks of cultures in the adipocyte differentiation medium, cultures were fixed in 4% paraformaldehyde. Cells differentiated into adipocytes were visualized and quantified under microscope according to the presence of refringent lipid droplets in the cytoplasm. 

#### 2.7.2. Osteoblast Lineage 

As for adipocyte differentiation, the fibroblast culture medium was substituted 48 h postconfluency by an osteoblast differentiation medium composed of MEM/10% fetal calf serum (PAN Biotech GmbH, Aidenbach, Germany); 2 mM β-glycerophosphate (Sigma, St Louis, MO, USA); and 10^−7^ M dexamethasone (Sigma, St Louis, MO, USA). After 3 weeks of cultures in the osteoblast differentiation medium, cultures were fixed in 4% paraformaldehyde. Cells differentiated into osteoblasts were visualized and counted after alizarin-red staining of the calcified extracellular matrix. 

#### 2.7.3. Chondrocyte Lineage 

For each sample, 10^5^ cells were centrifuged and kept as pellets for 24 h to initiate formation of spheroid structures. The fibroblast culture medium was then substituted by a chondrocyte differentiation medium composed of MEM; 0.5 µg/mL insulin (Gibco, France); 0.5 µg/mL transferrin (Sigma, St Louis, MO, USA); 0.5 ng/mL sodium selenite (Gibco, France); 6.25 µg/mL linoleic acid (Gibco, France); 6.25 µg/mL oleic acid (Gibco, France); 1.25 mg/mL bovine serum-albumin (Sigma, St Louis, MO, USA); 1 mM of sodium pyruvate (Gibco, France); 0.17 mM ascorbic acid 2-phosphate (Sigma, St Louis, MO, USA); 0.1 µM dexamethasone (Sigma, St Louis, MO, USA); 0.35 mM proline; and 0.01 µg/mL of TGF-β1 (RnD System, France). After 3 weeks of cultures in the chondrocyte differentiation medium, spheroids were included in OCT for cryosectioning. Chondrocyte differentiation was revealed by toluidine blue (Sigma, St Louis, MO, USA) and safranin O (Thermo-Fisher, France) staining and immunostaining of aggrecan (ACAN) and collagen XIα1 (ColXIα1). 

### 2.8. Transcriptome Analysis

#### 2.8.1. RNA Extraction 

Total RNA was extracted using the RNeasy kit (QIAgen, Courtaboeuf, France), using cultured fibroblasts at the stage of 7 to 10 population doublings. To limit the impact of experimental variations on gene expression profiles, culture conditions were standardized as follows: RNA extraction was systematically performed at 80% culture confluency and 24 h after a full medium renewal. Extracted RNA samples were split into aliquots in the perspective of microarray and qRT-PCR analyses. 

#### 2.8.2. Microarray Transcriptome Profiling 

Human full-genome Affymetrix GeneChip HG-U133 Plus 2.0 (PartnerChip, Evry, France) were used following the manufacturer’s recommendations. These microarrays contain 55000 probe sets (25 nucleotides per set) covering 30000 transcripts. Briefly, RNA quality and quantity were estimated using the Nanodrop (ND-1000) and BioAnalyzer 2100 systems (Agilent, Les Ulis, France). When too-high concentrations of salts or solvents were detected, RNA precipitation and washing were performed before sample processing. Quantification of array fluorescence signals was carried out using a GeneChip 3000 scanner. Then, array data were analyzed using the Affymetrix Command Console software. Quality control and statistical analyses were performed using the Affymetrix Expression Console and GeneSpring GX11 softwares.

#### 2.8.3. qRT-PCR

RNA samples were reverse-transcribed using the random primer and Superscript II Reverse transcriptase system following the manufacturer’s instructions (Invitrogen, France). Amplifications were performed using a MyiQ^TM^ LightCycler (Biorad, Marnes-la-Coquette, France). Real-time quantitative PCR was performed using a MyiQ^TM^ LightCycler (Biorad, Marnes-la-Coquette, France) and analyzed using the iQ^TM^ 5 software. Gene expression (primers listed in Table 1) was normalized according to *GAPDH* and *TBP* transcript levels.

### 2.9. Immunofluorescence

#### 2.9.1. Tissue Section Staining

Skin samples were fixed in neutral formalin and then embedded in a paraffin. Tissue sections of 5 µm thickness were prepared. For antibody staining, sections were permeabilized in 0.1% SDS after deparaffinization and epitope retrieval in a citrate buffer (pH = 6). To limit background signals, unspecific antibody fixation sites were saturated by sample incubation in 5% BSA. Sections were incubated with primary and secondary Alexa Fluor-coupled antibodies (see Table 2 for antibody references and working dilutions). Stained skin sections were mounted in ProLong Gold supplemented with DAPI (Thermo for Molecular Probes, Waltham, MA, USA, and images were acquired using a Leica microscope coupled with a QIMAGINE RETIGA 2000R Fast 1394 camera. Signal quantification was performed using ImageJ software. Quantification of cells positive for KLF9 expression was performed by visual counting on skin samples from 4 donors. Percentages of KLF9^+^ cells were determined in a total of 806 cells for Fr, 289 cells for Fr, and 246 cells for F-DHJ fibroblasts. 

#### 2.9.2. Cell Staining 

Cultured cells were fixed in 4% paraformaldehyde, permeabilized in 0.1% SDS, and incubated in 5% BSA for saturation of unspecific antibody binding sites and then with primary and secondary Alexa Fluor-coupled antibodies (see Table 2). Labeled cells were mounted in ProLong Gold supplemented with DAPI (Thermo - Molecular Probes, Waltham, MA, USA). Immunofluorescence images were acquired using a Leica microscope coupled with a QIMAGINE RETIGA 2000R Fast 1394 camera (QImageing, Canada). Signal quantification was performed using ImageJ software. 

#### 2.9.3. ECM Staining 

ECM slides were incubated in 5% BSA for saturation of unspecific antibody binding sites. Incubation with Alexa Fluor-coupled antibodies (Zenon technology – Thermo – Molecular Probes, Waltham, MA, USA) was performed during 30 min at room temperature (see Table 2). Immunofluorescence images were acquired using a Leica microscope coupled with a QIMAGINE RETIGA 2000R Fast 1394 camera (QImageing, Surrey, BC, Canada). Signal quantification was performed using ImageJ software. 

### 2.10. Western Blot Analysis

Expression of KLF9 was assessed by western blot analysis on total protein extracts from cell cultures. Protein extracts were prepared using a radioimmunoprecipitation assay (RIPA) buffer. Proteins (40 μg) were separated by 15% sodium dodecyl sulphate-polyacrylamide gel electrophoresis (PAGE) and electrotransferred onto a 0.45 µm nitrocellulose membrane. The membrane was incubated with the primary antibody, washed, and probed with the peroxidase-labeled secondary antibody. Detection was achieved by enhanced chemiluminescence (West Femto HRP substrates, ThermoFisher Scientific, France). After dehybridization, control loading was achieved by anti-glyceraldehyde-3-phosphate dehydrogenase antibodies. Densitometric analyses were performed using ImageJ.

### 2.11. Statistics 

Error bars represent SEM. The Wilcoxon-Mann-Whitney test and the Friedman test were applied to determine p-values. Data with *p* < 0.05 (*) or *p* < 0.01 (**) were considered as statistically significant. 

## 3. Results

### 3.1. Cellular Characteristics and Growth Potential Distinguish F-DHJ from Fp and Fr 

The cellular morphology of the three fibroblast populations were isolated based on their skin localization, i.e., papillary dermis fibroblasts (Fp), reticular dermis fibroblasts (Fr), and dermo-hypodermal junction fibroblasts (F-DHJ) were examined in cultures and compared (Figure 1C). As previously described [4], Fp cells exhibited a thin morphology, with bi or tricuspid shapes, whereas Fr had spread morphologies and stellate shapes. F-DHJ were more heterogeneous, from small tricuspids (red arrow) to larger cells with stellate shapes (white arrow) with visible trabecular networks. 

**Figure 2:**
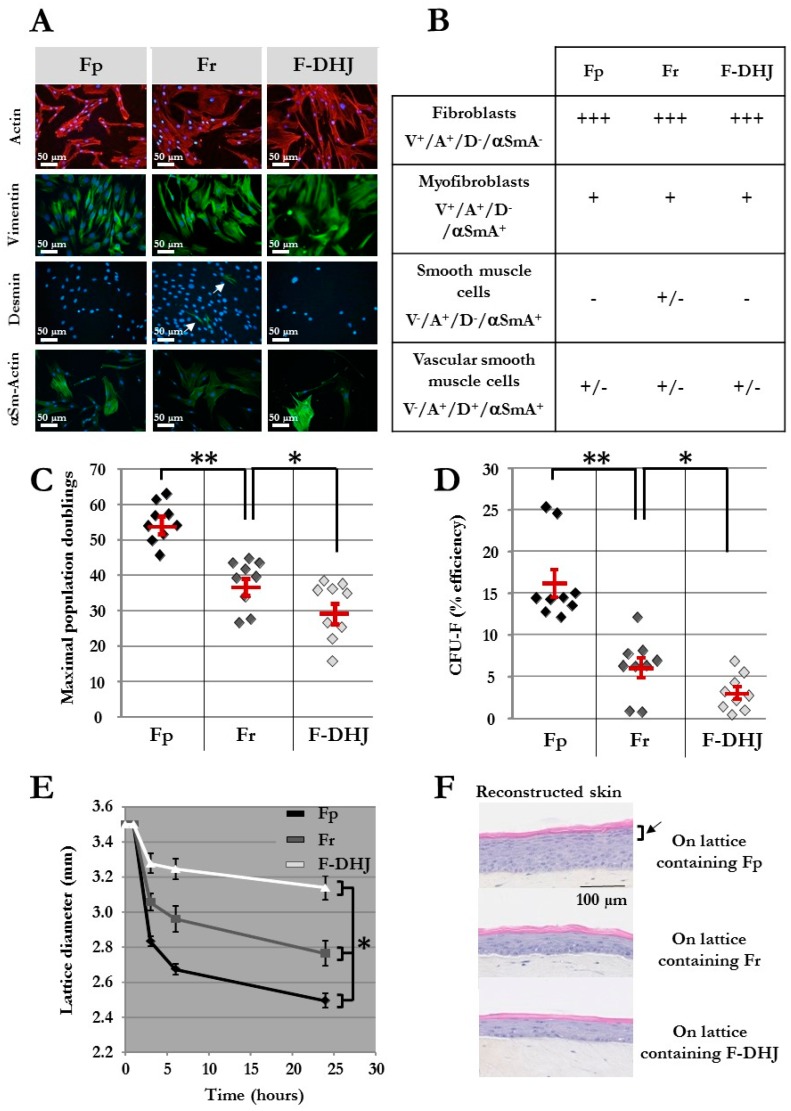
Phenotypic and functional properties of Fp, Fr, and F-DHJ fibroblasts. (**A**) Detection of actin (ACT) and vimentin (VIM), desmin (DES), and α-smooth muscle actin (α‑SMA) by immunochemistry in cultured Fp, Fr, and F-DHJ fibroblasts in the perspective of scoring according to Gabbiani’s classification [24]. White arrow points to rare DES+ cells present within the Fr population. (**B**) Scoring of Fp, Fr, and F‑DHJ fibroblasts according to ACT, VIM, DES, and α‑SMA detection: (−) = not present, (+/−) = low representation, (++) = frequent representation, and (+++) = major representation. (**C**) Long-term growth capacity of Fp, Fr, and F‑DHJ cells. Maximal cumulative population doubling values obtained with samples from independent donors are shown. Means ± SEM are indicated (* *p* < 0.05, ** *p* < 0.01; Wilcoxon test). (**D**) Colony-forming unit efficiency of Fp, Fr, and F‑DHJ cells. Fibroblast colony-forming unit (CFU-F) efficiency values (% of plated cells) obtained with samples from independent donors are shown. Means ± SEM are indicated (* *p* < 0.05, ** *p* < 0.01; Wilcoxon test). (**E**) Contractile capacity of Fp, Fr, and F‑DHJ cells in the 3D context of collagen lattices. Kinetics of lattice diameter evolutions. Means ± SEM are indicated (values obtained with samples from 9 independent donors) (* *p* < 0.05, Friedman’s test). (**F**) Efficiency of Fp, Fr, and F‑DHJ cells in promoting epidermis organogenesis by keratinocytes in a 3D reconstructed skin model. Representative reconstructed skin sections are shown (3 independent donors, each fibroblast sample tested in triplicates). The black arrow points to the epidermal granular layer that was obtained only in the presence of Fp fibroblasts.

Analysis of the four markers proposed in Gabbiani’s classification [24] (Figure 2A,B) confirmed the fibroblast statuses of the Fp, Fr, and F-DHJ cellular material, as all cell types expressed almost homogenously actin (ACT) and vimentin (VIM) but expressed neither desmin (DES) nor α-smooth muscle actin (α‑SMA): ACT^+^/VIM^+^/DES^−^/α‑SMA^−^ phenotype. In each population, only a minority of cells exhibited the myofibroblast ACT^+^/VIM^+^/DES^−^/α‑SMA^+^ phenotype, probably due to the cultures’ environments. Few cells corresponding to the ACT^+^/VIM^−^/DES^+^/α‑SMA^+^ vascular smooth muscle cell phenotype were also detected. In addition, the Fr population contained few ACT^+^/VIM^−^/DES^−^/α‑SMA^+^ cells, corresponding to smooth muscle cells probably originating from arrector pili muscles. 

The proliferative capacity of Fp, Fr, and F-DHJ cells was assessed in mass long-term cultures (Figure 2C) and using a colony assay (Figure 2D) (cell samples from n = 9 individuals were studied). As previously described [4,5], the proliferative capacity of Fr was lower than that of Fp, according to both criteria. Indeed, the maximum population doublings (PD) reached by Fp was 54 ± 2 versus 37 ± 2 for Fr (*p* < 0.01), and colony-forming efficiency was 16.2% ± 1.7 for Fp and 6.1% ± 1.2 for Fr (*p* < 0.01). F-DHJ exhibited the lowest growth capacity of the three fibroblast types, with a maximum PD reaching only 29 ± 3 and colony-forming efficiency 3% ± 0.7 (*p* < 0.05, calculated versus Fr). 

Taken together, these data show that Fp, Fr, and F-DHJ fibroblasts exhibit different cellular characteristics. 

### 3.2. Behavior in 3D Tissue and Differentiation Potential Distinguish F-DHJ from Fp and Fr 

A functional assay was designed to assess fibroblast contractile capacity in a three-dimensional environment based on a follow-up of collagen lattice contractions. Fp, Fr, and F-DHJ integrated in collagen lattices exhibited nonequivalent contraction behaviors (Figure 2E) (cell samples from n = 9 individuals were tested). Reduction of the lattice diameter was more rapid and marked with Fp than with Fr cells. In contrast, a more moderate lattice diameter reduction was observed with F-DHJ cells, indicating a lower contractile capacity for this fibroblast population. The three lattice contraction curves showed statistically significant differences (*p* < 0.05).

The next functional property of Fp, Fr, and F-DHJ that was investigated and compared was their efficiency in promoting epidermis organogenesis by keratinocytes in a model of in vitro three-dimensional skin reconstruction. Lattices containing either Fp, Fr, or F-DHJ cells were produced and used as dermal equivalents. On top of which, keratinocytes were then plated in order to obtain epidermis development (Figure 2F) (fibroblasts samples from n = 3 individuals were tested, in association with keratinocytes from a single donor). Fp cells were the most efficient fibroblast population for promoting the development of a correctly differentiated epidermis comprising a regular basal layer, as well as fully differentiated granular and horny layers. Epidermis reconstructs were of a lower quality with dermal lattices containing Fr fibroblasts; basal keratinocytes were of bigger sizes and less regular, and differentiation of the granular layer was incomplete. Dermal lattices populated with F-DHJ promoted poor epidermis stratification and differentiation. 

**Figure 3:**
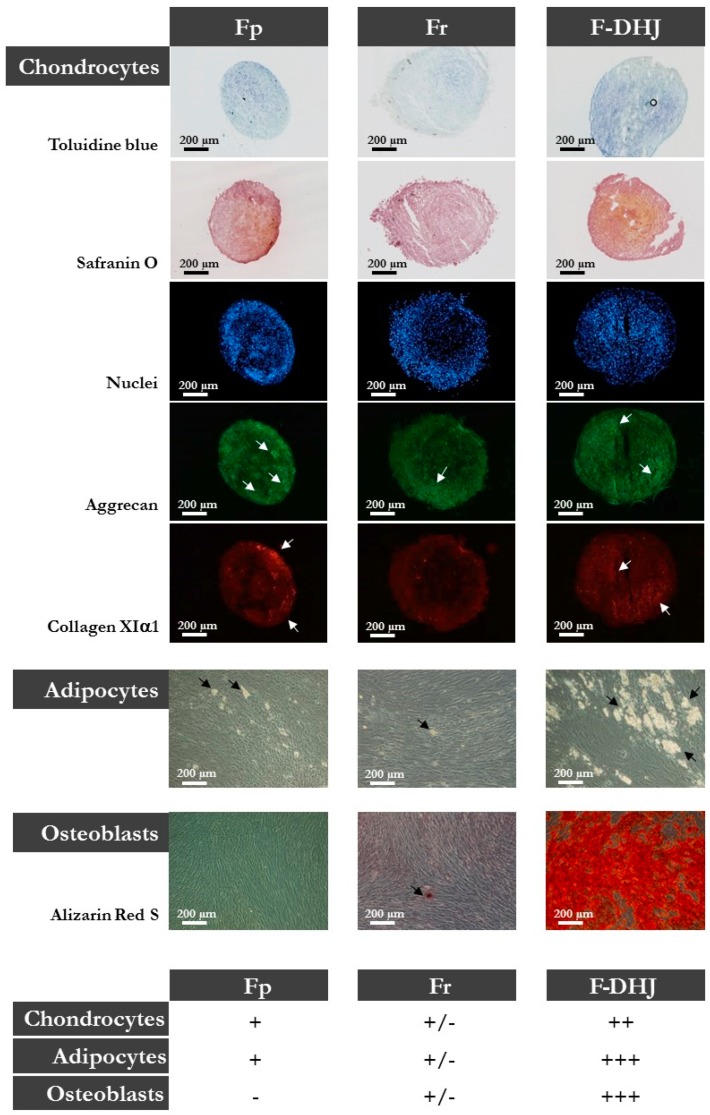
Differentiation capacities of Fp, Fr, and F-DHJ fibroblasts into mesodermal lineages. Samples from 5 independent “young” donors (20, 22, 25, 28, and 31 years old) were tested for their capacity to differentiate into chondrocytes (toluidine blue and safranin O staining, aggrecan (ACAN) and collagen XIα1 (ColXIα1) expression, white arrows); adipocytes (presence of cytoplasmic lipid droplets, black arrows); and osteoblasts (alizarin-red staining). Scoring of differentiation capabilities are presented: (−) = not present, (+/−) = low representation, (++) = frequent representation, and (+++) = major representation.

Finally, Fp, Fr, and F-DHJ were studied for their differentiation capacity into three mesodermal cell lineages: adipocytes (presence of cytoplasmic lipid droplets); osteoblasts (alizarin-red staining); and chondrocytes (toluidine blue and safranin O staining, aggrecan (ACAN) and collagen XIα1 (ColXIα1) expression). This functionality was documented using cells obtained from skin biopsies corresponding to ages ranging between 20 and 31 years (Figure 3). Interestingly, F‑DHJ exhibited a wider differentiation potential than that of Fp and Fr fibroblasts, as these cells efficiently responded to the three lineage-oriented differentiation conditions. Fp fibroblasts gave rise to fewer quantities of adipocytes and chondrocytes and did not differentiate into osteoblasts. Fr fibroblasts could give rise to differentiated cells of the three lineages but with a much lower efficiency than F-DHJ cells. 

Taken together, these data show that Fp, Fr, and F-DHJ fibroblasts exhibit different functional characteristics. 

### 3.3. Molecular Profiles Distinguish the Fp, Fr, and F-DHJ Fibroblast Populations

**Figure 4:**
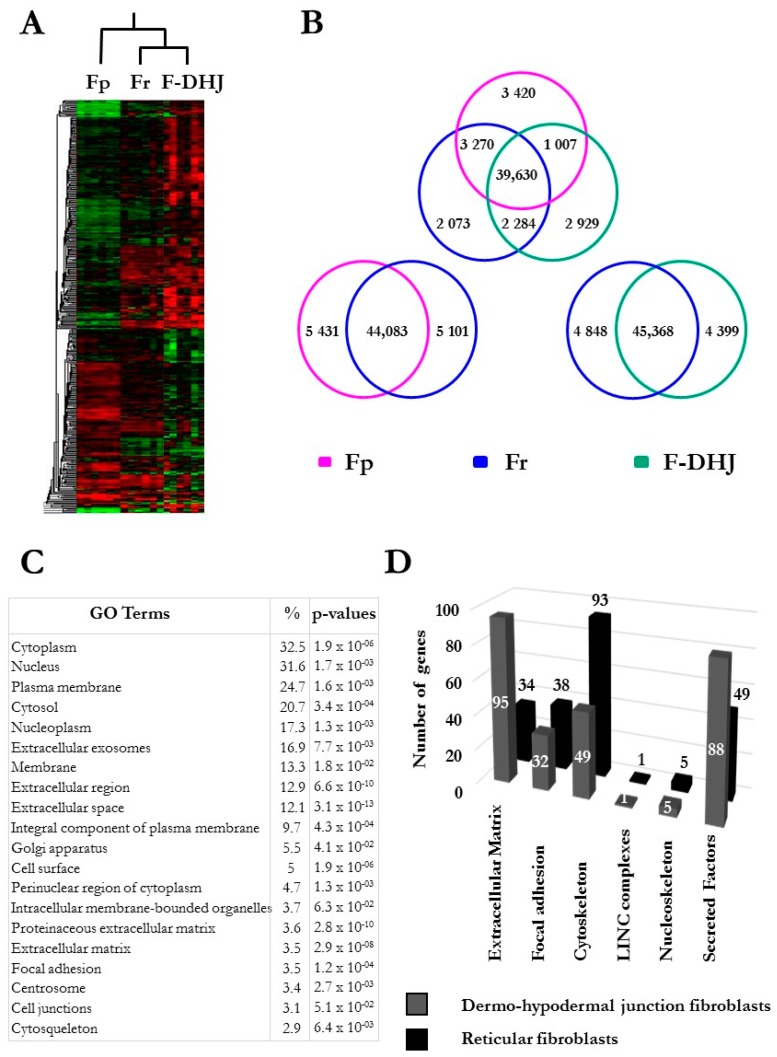
Microarray analysis of the transcriptome profiles of Fp, Fr, and F-DHJ fibroblasts (donors’ ages: 22, 25, 28, 55, 61, and 65 y.o.). (**A**) Hierarchical clustering based on 1078 differentially expressed transcripts (fold-change cutoff at 3 and *p* < 0.05). (**B**) Venn Diagrams summarizing Fp, Fr, and F-DHJ-enriched transcriptional signatures (*p* < 0.05). (**C**) List of the 20 most significant gene ontology (GO) terms differentiating F-DHJ from Fr cells, based on 2540 probe sets (1647 transcripts) exhibiting differential signals (fold-change >1.5 and *p* < 0.05). (**D**) Signatures identifying Fr fibroblasts (black bars) and F-DHJ fibroblasts (grey bars) among transcripts related to the tissue skeleton biology (fold-change >1.5 and *p* < 0.05).

**Figure 5:**
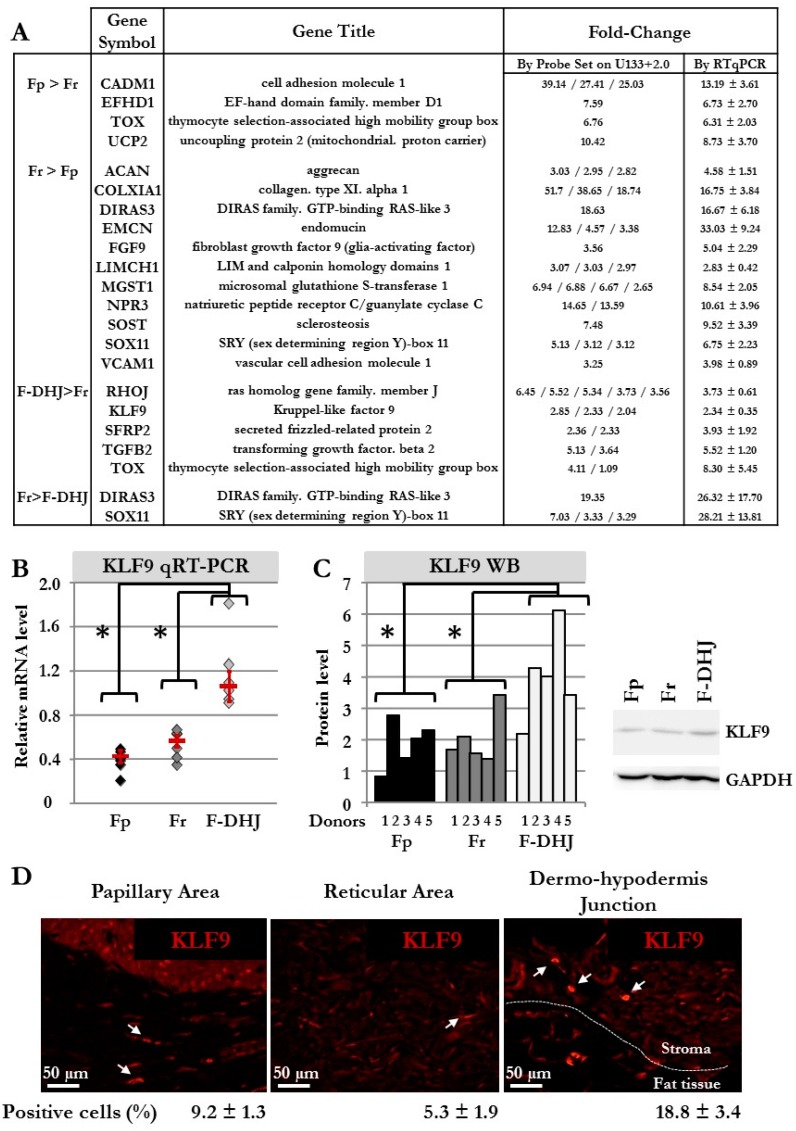
Biomarker validations at mRNA and protein levels. (**A**) Selection of transcripts in which differential expression was confirmed by qRT-PCR in cell samples from 6 donors (donors’ ages: 22, 25, 28, 55, 61, and 65 y.o). (**B**) Detailed qRT-PCR comparative analysis of the *KLF9* transcript in cells from the 6 donors (donors’ ages: 22, 25, 28, 55, 61, and 65 y.o). Means ± SEM are indicated (* *p* < 0.05, Wilcoxon test). (**C**) Western blot comparative analysis of the KLF9 protein in cells from the 5 donors (* *p* < 0.05, Wilcoxon test). A histogram detailing quantifications and a representative western blot gel is shown. (**D**) Immunofluorescence detection of the KLF9 protein in skin sections (breast). The percentage of KLF9^+^ cells was determined by observation under a fluorescence microscope of a total of 806 cells for Fp, 289 cells for Fr, and 246 cells for F-DHJ fibroblasts (skin sections from 4 donors were included in the analysis).

The molecular profiles of Fp, Fr, and F-DHJ cells were characterized and compared by microarray genome-wide transcriptome profiling (Figure 4 and Figure 5A). In the perspective of identifying molecular signatures distinguishing the Fp, Fr, and F-DHJ fibroblast populations whatever the donor’s age, the selected donor cohort covered both young and older ages: 22, 25, 28, 55, 61, and 65 y.o. As a first screen, a fold-change threshold value of three, together with a *p*-value of 0.05, were used to identify differential signals. According to these filters, a hierarchical clustering was built based on expression levels of 1078 transcripts, identifying signatures that validated at the transcriptome level of the distinct natures of Fp, Fr, and F-DHJ (Figure 4A). Next, transcriptome data were reanalyzed considering only the statistical significance threshold (*p*-value < 0.05) independently of fold-change values (Figure 4B). This analysis identified 3420, 2073, and 2929 probe sets, which could be used to define signatures of Fp, Fr, and F-DHJ cells, respectively. Fr cells shared the highest level of commonalities with the other fibroblast populations, probably due to their intermediate tissue localization: 3270 probe sets in common with Fp (not detected in F-DHJ) and 2284 probe sets in common with F-DHJ (not detected in Fp). 

F-DHJ were then compared more specifically with Fr cells, which are in spatial proximity in the tissue. A gene ontology (GO) term analysis was performed based on 2540 probe sets (1647 genes) exhibiting differential signals between the two populations (parameters: fold-change >1.5 and *p*-value < 0.05) (Figure 4C). Notably, this analysis revealed marked differences between Fr and F‑DHJ concerning the expression of transcripts related to the tissue skeleton (see [9]), as 26% of the transcripts differentially expressed were linked to the structuration of this network (Figure 4D and Table 3). In particular, differentially expressed probe sets were enriched in transcripts related to ECM components, cytoskeleton, and secreted factors. 

For validation of our microarray data, 19 genes were selected from the signatures that distinguished Fp, Fr, and F-DHJ identities, and transcript levels were analyzed by qRT-PCR in cell samples from the six donors (Figure 5A,B). Validation of microarray data was obtained for the 19 selected transcripts. As an attempt to identify a biomarker of F-DHJ cells, a focus was made on KLF9, which the transcript was detected as overexpressed in F-DHJ versus Fr by both technics in the six tested donors. The transcription factor KLF9 regulates the early phases of adipocyte differentiation [25], and thus, attracted attention due to the proximity of F-DHJ cells with hypodermis adipose tissue. Expression of KLF9 was analyzed at the protein level by western blot in cultured cells from six donors (Figure 5C) and by immune-fluorescence in skin biopsies from four individuals (Figure 5D). As expected from transcriptome data, the KLF9 protein was expressed at a higher level in cultured F-DHJ than in cultured Fp and Fr (*p* < 0.05). In skin sections, the percentage of cells expressing KLF9 was higher in F-DHJ than in Fp and Fr regions, respectively 18.8 ± 3.4% versus 9.2 ± 1.3% and 5.3 ± 1.9%. 

### 3.4. The Dermo-Hypodermal Junction and Reticular Dermis Differ in Their Matrix Architectural Meshwork

**Figure 6:**
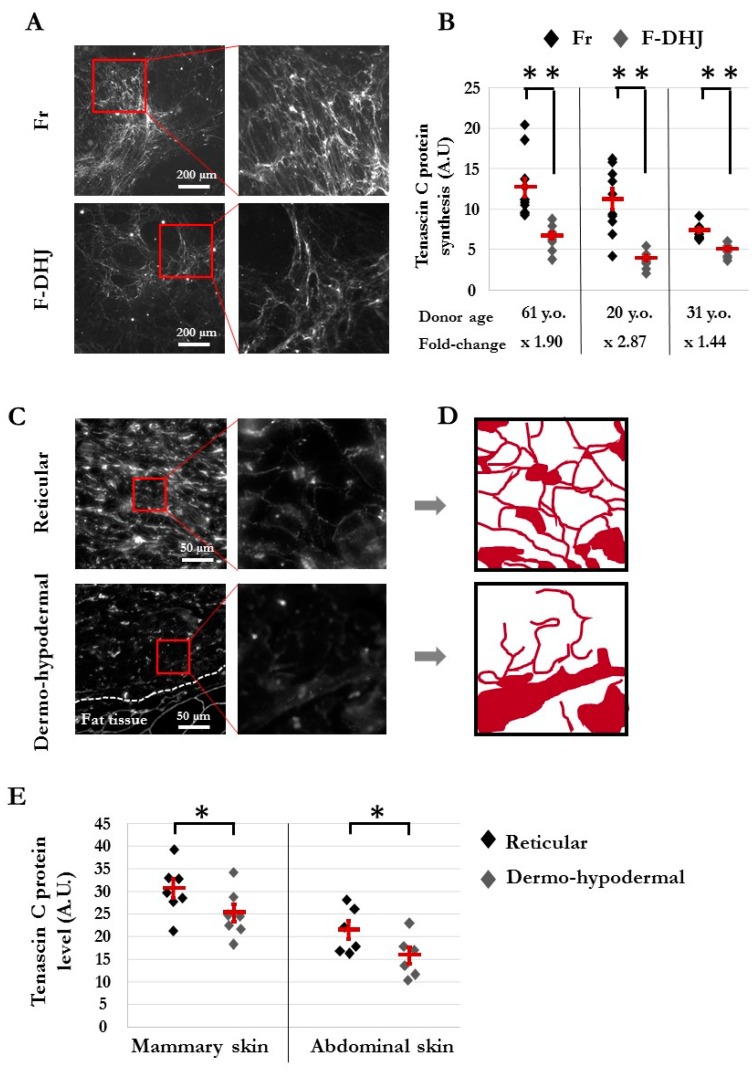
Architecture of the tenascin C (TNC) meshwork produced in vitro by Fr and F-DHJ fibroblasts in skin sections. (**A**) Immunostaining pictures of the TNC meshwork produced by Fr and F-DHJ cells in 2D cultures. (**B**) Quantification of TNC secreted in 2D cultures. Cell samples from 3 donors were used. Values corresponding to 10 replicate analyses for each cell sample are shown. Means ± SEM are indicated (** *p* < 0.01, Wilcoxon test) A.U. for arbitrary units. (**C**) Photographs of TNC immunostaining in skin sections, illustrating the structural differences between reticular dermis and the dermo-hypodermal junction area (representative from 13 analyzed donors). (**D**) Image reconstitution of TNC meshwork architectures based on the immunostaining photographs shown in panel (**C**). (**E**) Quantification of TNC in the skin reticular and dermo-hypodermal areas. Values obtained from the analysis of skin samples from 13 donors are shown. Samples from two anatomical localizations: breast skin (7 donors of ages between 18 and 65 years) and abdominal skin (6 donors of ages between 42 and 51 years). No age-related changes in TNC synthesis/meshwork were observed. Means ± SEM are indicated (* *p* < 0.05, Wilcoxon test) A.U. for arbitrary units.

From the lists of transcripts differentially expressed between F-DHJ and Fr, our attention was attracted by tenascin C (TNC), considering its major role in the organization of collagen fibril anchoring points. Indeed, TNC forms a typical disulfide-linked hexamer, called the hexabrachion, in which six flexible arms emanate from a central globular particle, which possibly catches and stabilizes a bifurcation of the ECM fibrils composed of FN1 and type I collagen to underlie the extracellular meshwork architecture (for review, see [26]). Our transcriptome analysis indicated a 2.61-fold lower expression of TNC in F-DHJ versus Fr cells (Table 3). To explore this property at the protein level, immunostaining of TNC was performed on samples of ECM synthesized by F-DHJ and Fr cells in vitro (Figure 6A,B) (cells from n = 3 individuals were tested). Notably, reticulation of TNC was more marked in ECM samples synthesized by Fr than in ECM secreted by F-DHJ (Figure 6A). Moreover, signal quantification indicated TNC levels lower in ECM produced by F-DHJ versus Fr (*p* < 0.01) (Figure 6B). 

Architectural differences between the DHJ and reticular areas were confirmed in skin sections (Figure 6C–E). In the reticular area, TNC protein-staining revealed a thin mesh structuration around collagen bundles in agreement with the alveolar organization of this dermal territory, whereas this structuration was not present in the DHJ area (Figure 6C,D). In addition, quantification of the TNC immunostaining signals performed in sections of mammary skin (biopsies from seven individuals) and abdominal skin (six individuals) indicated a higher level of TNC in the reticular dermis area than in the HDJ area for both skin anatomical origins (*p* < 0.05) (Figure 6E). 

### 3.5. F-DHJ Fibroblasts and Adipose-Derived MSCs Exhibit Distinct Transcriptome Profiles 

**Figure 7:**
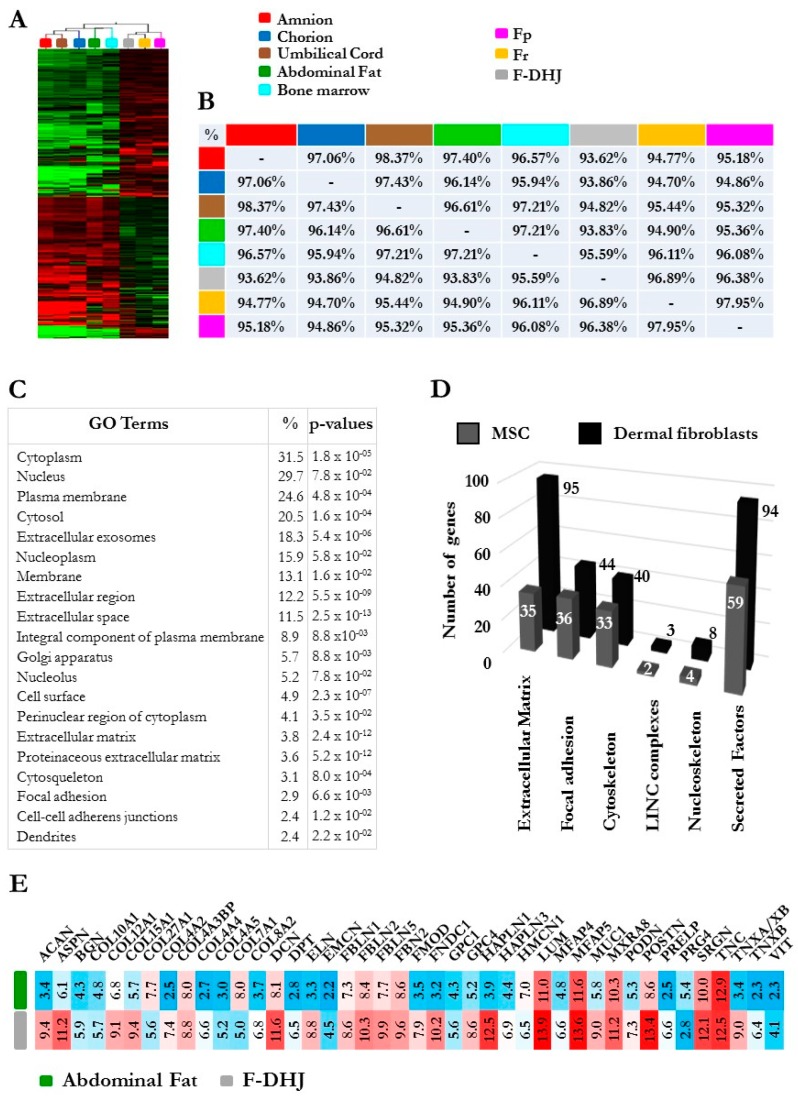
Comparative microarray transcriptome profiling of the three fibroblasts populations (Fp, Fr, and F-DHJ) and mesenchymal stem cell (MSC) samples corresponding to five sources (bone marrow aspirates, adipose tissue, amnion, chorion, and umbilical cord jelly). (**A**) Hierarchical clustering of fibroblast and MSC samples based on the 380 most discriminant probe sets showing a marked distinction between the “fibroblast” and “MSC” groups. (**B**) Pearson correlation coefficients evaluating sample-to-sample proximity based on comparisons of global transcriptome profiles. Notably, this analysis showed the low proximity between F-DHJ with adipose tissue MSCs (93.83% similarity) and high proximity with Fr fibroblasts (96.89% similarity). (**C**) List of the 20 most significant gene ontology (GO) terms differentiating the “fibroblast” and “MSC” groups based on 2974 probe sets (1984 transcripts) exhibiting differential signals (fold-change > 2 and *p* < 0.05). (**D**) Signatures identifying the “fibroblast” group (black bars) and the “MSC” group (grey bars) among transcripts related to the tissue skeleton biology (fold-change >2 and *p* < 0.05). (**E**) Focus on 42 transcripts directly involved in the structuration and composition of the ECM network and identified within the signature that distinguishes the “fibroblast” and “MSC” groups. Values were obtained by GCRMA microarray signals and corresponded to an indication of transcript levels (arbitrary units) in F-DHJ and adipose MSCs.

Given the anatomical proximity between F-DHJ and MSCs derived from hypodermal adipose tissues, their molecular characteristics were explored at the level of the global transcriptome to determine whether these two cell populations have a distinct identity or not. To widen this question, the three fibroblast types (Fp, Fr, and F-DHJ) were analyzed together with MSC samples corresponding to five sources (bone marrow aspirates, adipose tissue, amnion, chorion, and umbilical cord jelly) (Figure 7). A hierarchical clustering based on 380 discriminant probe sets revealed a clear segmentation between the “fibroblast” group and the “MSC” group (Figure 7A), which confirmed the distinct identities of F-DHJ and adipose MSCs. Within the “fibroblast” group, F-DHJ appeared more similar with Fr than they were with Fp cells. Within the “MSC” group, cells from the three fetal origins (amnion, chorion, and cord) were more similar to each other than they were with the two adult origins (marrow and adipose). This clustering was confirmed when a full transcriptome analysis was considered (Pearson correlation coefficients) (Figure 7B). To document biological characteristics distinguishing the fibroblast and MSC groups, a gene ontology (GO) term analysis was performed based on 2974 probe sets (1984 genes) distinguishing the two sample groups (parameters: fold-change >2 and *p*-value <0.05). Among the twenty most significant GO terms, transcripts related to structuration of the tissue skeleton were largely represented, including numerous ECM, focal adhesion, cytoskeleton, LINC complexes, nucleoskeleton, and secreted factor transcripts, in which their levels distinguish fibroblasts from MSCs (Figure 7C,D and Table 4). In particular, a signature of 42 transcripts directly related to ECM structure and composition was identified (Figure 7E), constituting a pool of candidates to further explore the biological differences between F-DHJ and adipose MSCs. 

### 3.6. Differentiation Capacity is Reduced in F-DHJ from Aged Skin 

**Figure 8:**
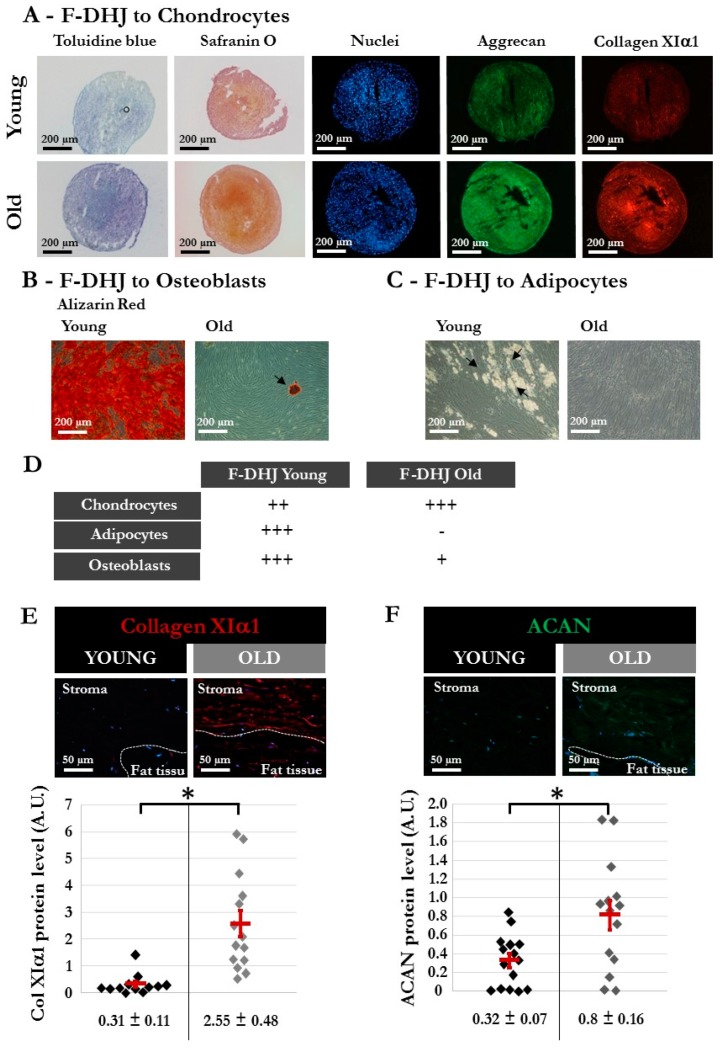
Different characteristics of DHJ components in skin from “young” and “old” donors. The “young” group comprised 5 donors (20, 22, 25, 28, and 31 years old) (same donors as in Figure 3), and the “old” group comprised 4 donors (55, 61, 65, and 65 years old). (**A–C**) Capacity of “young” and “old” F-DHJ cells to differentiate in vitro into three mesenchymal lineages: (**A**) chondrocytes (toluidine blue and safranin O staining, aggrecan (ACAN) and collagen XIα1 (ColXIα1) expression); (**B**) adipocytes (presence of cytoplasmic lipid droplets, black arrows); and (**C**) osteoblasts (alizarin-red staining). For panels (**A–C**), representative photographs are shown. (**D**) Summary of the differentiation capacity into chondrocytes, adipocytes, and osteoblasts of F-DHJ from “young” and “old” skin. Scoring of differentiation capabilities are presented: (−) = not present, (+) = low representation, (++) = frequent representation, and (+++) = major representation. (**E**,**F**) Immunofluorescence detection of ColXIα1 (**E**) and ACAN (**F**) in skin biopsies from “young” and “old” donors. Representative photographs are shown, in association with quantification values corresponding to a total of 12 (**E**) and 15 (**F**) regions of interest (ROI) for the 3 analyzed “young” donors (20, 22, and 28 years old) and a total of 14 (**E**) and 15 (**F**) ROI for the 3 analyzed “old” donors (57, 61, and 65 years old). Means ± SEM are indicated (* *p* < 0.05, Wilcoxon test).

Finally, the capacity for differentiation into the adipocyte, osteoblast, and chondrocyte lineages was compared in F-DHJ samples from “young” (between 20 and 31 years, n = five donors) and “older” (between 55 and 65 years, n = four donors) ages (Figure 8). The three-lineage mesenchymal differentiation potential of F-DHJ described in Figure 3 appeared altered in cell samples from older skin biopsies (Figure 8A–D). Although chondrocyte differentiation was maintained (Figure 8A–D), the capacity for differentiation into osteoblasts was reduced (Figure 8B,D) and differentiation into the adipocyte lineage was almost lost (Figure 8C,D). A comparative analysis of the differentiation potential of Fp, Fr, and F-DHJ from old donors indicated functional differences (data not shown). The capacity for differentiation into adipocytes persisted with a low efficiency in old Fp and Fr, although it was lost with age in F-DHJ. In contrast, differentiation into osteoblasts was not obtained with old Fp or Fr, whereas this capacity was present in old F-DHJ. Finally, we observed that the capacity for differentiation into chondrocytes was increased with age in the three cell types but remained more efficient in F-DHJ, as compared with Fp and Fr cells. In addition to these age-related changes in the F-DHJ differentiation potential, the extracellular deposition of ColXIα1 and ACAN were respectively 3.6-fold and 2.5-fold higher in skin biopsies from the older than in the young donor group (*p* < 0.05) (Figure 8E,F). These observations pinpoint the interest of considering F-DHJ cells in future studies on skin ageing. 

## 4. Discussion

The present work investigates the properties of a fibroblast compartment localized within the conjunctival junctions that connects the dermis to the hypodermis, i.e., dermo-hypodermal junction fibroblasts (F-DHJ), which were compared to intermediate reticular dermis (Fr) and superficial papillary dermis (Fp) fibroblasts. Cellular functional assays, combined with transcriptome profiling, indicated that F-DHJ had distinct characteristics from those of Fp and Fr cells. F-DHJ had the lowest proliferation and clonogenic capacity of the three fibroblast populations in bidimensional culture conditions. Moreover, when integrated within the dermal component of an in vitro three-dimensional reconstructed skin model, F-DHJ showed a low capacity for collagen lattice contractions and had a poor capacity for promoting epidermis organogenesis by keratinocytes. Inefficient dialog with keratinocytes observed here in vitro is in agreement with F-DHJ natural deep localizations, which are not in proximity with the epidermis, unlike the superficial Fp population. The lattice contraction assay provided the opportunity to assess the contractile capacity of specific cell types in a three-dimensional matrix environment. The contraction of the lattice is proportional to the force exerted by the cells in the matrix. Parameters that impact lattice contractions include characteristics of cell matrix anchoring structures, cytoskeleton organization, and the capacity of cells to coordinate and exert unidirectional forces. These parameters are governed by components of the “tissue skeleton” network [8,9] and may participate in vivo to confer specific biophysical characteristics to the different dermal tissue compartments. Extrapolation of the in vitro observations to the specific in vivo functions of Fp, Fr, and F-DHJ will require further studies, considering the high matrix complexity of the dermis. 

We observed that F-DHJ exhibited an efficient capacity for three-lineage mesenchymal differentiation (i.e., adipocyte, osteoblast, and chondrocyte lineages), which could be interpreted as an MSC-like cellular identity, considering their anatomical proximity with the hypodermis, a tissue that contains adipose MSCs. Interestingly, the hierarchical clustering built on the basis of the transcriptome profiles of the three skin fibroblast populations (Fp, Fr, and F-DHJ) and five MSC origins (bone marrow, adipose, amnion, chorion, and cord) indicated a clear “fibroblast” molecular identity of F-DHJ, which did not segregate together with the MSC group. 

The molecular signature that identified F-DHJ cells comprised transcripts involved in the stabilization of monomeric proteoglycan aggregates associated with hyaluronic acid molecules, such as *HAPLN1* and *HAPLN3* [27], which were found overexpressed in F‑DHJ in comparison with all MSC types. Transcripts overexpressed in F-DHJ also included *ACAN*, which is involved in conferring tissue biomechanical resistance [28]. In addition, the overexpressed F-DHJ signature also comprised transcripts related to the collagen meshwork, such as *FMOD* and *TNX*, which are involved in collagen processing [29]; transcripts related to collagen fibril anchorage points, such as *POSTN* and *FNDC1* [26]; and transcripts related to the elastic network, such as *ELN*; *DCN*; *MFAP4* and *5*; *FBN2*; and *FBLN1*, *2*, and *5* [30]. On the contrary, the comparison of F-DHJ and Fr molecular profiles identified a signature of transcripts underexpressed in the F-DHJ population, which could be interpreted in accordance with the reduced ECM mesh structuration within the DHJ area, in comparison with the reticular dermis. Notably, this character was documented by lower levels of the *TNC* transcript in F-DHJ than in Fr cells, which is associated with a lower accumulation of the TNC protein and loss of the TNC network in the DHJ area. Thus, the molecular specificities that distinguish F-DHJ and Fr cells may contribute to the different ECM characteristics of the reticular dermis and DHJ areas. 

The existence of a fibroblast population exhibiting adipocyte-like molecular characteristics within the deep reticular dermis has been reported both in mouse [31,32] and human skin [33,34]. In human skin, the capacity for adipocyte differentiation was reported to be low for FAP^+^/CD90^-^ papillary fibroblasts, intermediate for FAP^+^/CD90^+^ fibroblasts from the superior reticular dermis, and high for FAP^-^/CD90^+^ deep reticular dermis fibroblasts [33]. This gradation is consistent with the data shown in the present study, showing a correlation between the capacity for adipocyte differentiation and the depth of fibroblast dermal localization. The study by Korosec et al., which used cells from skin donors of ages ranging between 26 and 61 years, did not report an age-related reduction of the adipocyte differentiation capacity [33], as documented here for F-DHJ cells, although this phenomenon has been previously described for dermal fibroblast cells [35]. In the present study, “F-DHJ” is used to name the fibroblast population that we isolated according to its junctional localization between the deep reticular dermis and the hypodermis. This terminology distinguishes the deepest dermal part from the reticular dermis compartment, which is in agreement with their particular molecular and functional characteristics that may be critical for modeling their local ECM environment. 

Interestingly, data were obtained pointing to age-related changes in the DHJ region characteristics, such as augmented levels of the ECM proteins ColXIα1 and ACAN and a reduced adipocyte differentiation potential of F-DHJ in old skin. Data from the literature concerning the evolution of the dermal fibroblast capacity for differentiation into adipocytes can appear contradictory, with regard to our observation of a decreased adipogenic potential. Indeed, a study performed on mice has, on the contrary, reported the acquisition of proadipogenic traits in dermal fibroblasts from aged animals [32], in which the difference may result from physiological species-related specificities. In a recent study, a single-cell RNA-sequencing analysis of 15,000 dermal fibroblasts isolated from human skin samples from young and old donors did not detect an up-modulation of adipogenic genes associated with ageing [36]. Of note, in humans, subcutaneous fat tissue masses tend to reduce with ageing, in particular in the face (for review, see [37]). 

As we performed here using collagen lattices as a dermal matrix model, human fibroblasts isolated from the deep dermis were used to populate acellular dead desepidermized dermis (DED) pieces and analyzed for their capacity to support epidermis reconstruction by keratinocytes [34]. The two studies converged to show that fibroblasts from the deep dermis do not promote the formation of a correctly differentiated multilayered epithelium, which is consistent with their distant skin localization. Interestingly, deep dermis fibroblasts spontaneously populated the deepest part of the DED [34], in which homing may be due to the recognition of specific ECM characteristics. 

Fibroblast-ECM interrelations are crucial for the maintenance of dermal integrity. In a mouse model, dermal fibroblasts were studied by intravital time-lapse, which revealed active membrane dynamics characterized by protrusions that rapidly grow and shrink from a more stable cell body [38]. By this process, fibroblasts may dialog with their cellular and ECM neighbors, and thus, adapt their behaviors and fate. Accordingly, the development of membrane extensions in living cells has been proposed to compensate for the appearance of cell-free volumes due to fibroblast deaths in the dermis of aged skin [38]. These observations may be explored at a molecular level considering genes related to the network termed as “tissue skeleton” that connects the cells with their tissue environment (comprising the nucleoskeleton, the cytoskeleton, linker complexes, ECM components, and focal adhesion points), in which their expressions differ in fibroblasts according to their dermal localization and evolve with ageing (present study and [8,9]. Disruption of this multiparametric network of interactions may result in changes that affect aged dermis, including the loss of contact surfaces between fibroblasts and their surrounding ECM [39] and modification of the deposition of ECM components, such as ColXIα1 and ACAN, as shown here. 

## 5. Patent 

V.H. and D.A. are the inventors on the filed patent application numbered 1759023 (28th September 2017) entitled “Molecular signatures of aging of 3 subpopulations of dermal fibroblasts (papillary, reticular, dermo-hypodermic junction) and dermal equivalents comprising aged fibroblasts”.

V.H is the inventor on the filed patent application numbered 1855987 (June 29th 2018) entitled “Modèle de peau comprenant des fibroblastes de la jonction dermo-hypodermique pour l’identification d’actif pro-différenciant vers des lignages adipocytaire, chondroblastique et ostéoblastique”.

## Figures and Tables

**Table 1:** qRT-PCR primers. Primer list and references are provided.

Gene Symbol	Supplier Reference
*ACAN*	QT00001365
*CADM1*	QT00050001
*COL11A1*	QT00088711
*DIRAS3*	QT00040558
*EFHD1*	QT00086163
*EMCN*	QT00025158
*FGF9*	QT00000091
*GAPDH*	QT01192646
*KLF9*	QT00208537
*LIMCH1*	QT00038794
*MGST1*	QT00063357
*NPR3*	QT00047250
*RHOJ*	QT00092078
*SFRP2*	QT00073220
*SOST*	QT00219968
*SOX11*	QT00221466
*TBP*	QT00000721
*TGFB2*	QT02290316
*TOX*	QT00070063
*UCP2*	QT00014140
*VCAM1*	QT00018347

**Table 2:** Antibodies. Antibody references and working dilutions are provided.

Protein name	Supplier	Reference	Dilution
alpha Sm actin	Sigma (Saint-Quentin Falaviers—France)	A5228	1/200
ACAN (Aggrecan) *	Abcam (Paris—France)	ab3778	1/20
Col XI a1	Sigma (Saint-Quentin Falaviers—France)	SAB4500393	1/50
Desmine (clone D33)	Dako—Agilent (France)	M0760	1/50
GAPDH	Interchim for Meridian (France)	H86504M	1/2000
KLF9	Abcam (Paris—France)	ab170980	1/100 (IHC)–1/1000 (WB)
Phalloïdine Rhodamin	Invitrogen (France)	R415	1/50
TNC (Tenascin C)	Novus Biologicals (Abington—UK)	NB110-68136	1/50
Vimentin	TEBU (Le Perray-en-Yvelines—France)	MON3005	1/10
Goat anti-Mouse Alexa 488	Molecular Probes Invitrogen (France)	A21121	1/250
Goat anti-Rabbit Alexa 555	Molecular Probes Invitrogen (France)	A21428	1/250
Zenon Alexa 488	Molecular Probes Invitrogen (France)	Z25002	
Goat anti-Rabbit HRP	Thermo-Fisher, France	32460	1/2000

* Pre-processing: keratanase (0.1 U/mL) + chondroitinase (0.1 U/mL)—3 h—37 °C.

**Table 3:** Transcripts related to the tissue skeleton differentially expressed in reticular dermis fibroblasts (Fr) and dermo-hypodermal junction fibroblasts (F-DHJ). This transcript list was extracted from microarray data using a fold-change >1.5 and *p* < 0.05 as inclusion parameters. The transcript signature with predominant expression in Fr cells concerned 297 probe sets corresponding to transcripts directly involved in the tissue skeleton structure, comprising 33 transcripts related to the extracellular matrix (ECM), 125 focal adhesion point transcripts, 60 cytoskeleton transcripts, 1 LINC complex transcript, and 8 nucleoskeleton transcripts. The transcript signature with predominant expression in F-DHJ cells concerned 359 probe sets corresponding to transcripts directly involved in the tissue skeleton structure, comprising 94 transcripts related to ECM, 76 focal adhesion point transcripts, 50 cytoskeleton transcripts, 1 LINC complex transcript, and 7 nucleoskeleton transcripts. In addition, transcripts encoding soluble factors were found in both signatures, respectively 70 and 131 for Fr and F-DHJ cells.

UP in Fr					UP in F-DHJ				
**Extracellular Matrix Genes**									
**Probe Set ID**	**Gene Symbol**	**Gene Title**	**adj-pval**	**FC**	**Probe Set ID**	**Gene Symbol**	**Gene Title**	**adj-pval**	**FC**
205941_s_at	COL10A1	collagen, type X, alpha 1	9.41 × 10^−3^	4.23	220518_at	ABI3BP	ABI family, member 3 (NESH) binding protein	2.37 × 10^−2^	2.92
211343_s_at	COL13A1	collagen, type XIII, alpha 1	1.44 × 10^−2^	2.26	1559077_at	ABI3BP	ABI family, member 3 (NESH) binding protein	7.43 × 10^−2^	2.49
211809_x_at	COL13A1	collagen, type XIII, alpha 1	2.74 × 10^−2^	1.74	222486_s_at	ADAMTS1	ADAM metallopeptidase with thrombospondin type 1 motif, 1	2.76 × 10^−2^	2.33
221900_at	COL8A2	collagen, type VIII, alpha 2	2.88 × 10^−1^	2.23	222162_s_at	ADAMTS1	ADAM metallopeptidase with thrombospondin type 1 motif, 1	4.36 × 10^−2^	1.91
226824_at	CPXM2	carboxypeptidase X (M14 family), member 2	2.01 × 10^−1^	1.88	226997_at	ADAMTS12	ADAM metallopeptidase with thrombospondin type 1 motif, 12	3.75 × 10^−3^	2.78
221541_at	CRISPLD2	cysteine-rich secretory protein LCCL domain containing 2	5.98 × 10^−2^	2.03	214913_at	ADAMTS3	ADAM metallopeptidase with thrombospondin type 1 motif, 3	1.94 × 10^−2^	1.94
206595_at	CST6	cystatin E/M	2.34 × 10^−2^	3.12	237411_at	ADAMTS6	ADAM metallopeptidase with thrombospondin type 1 motif, 6	1.36 × 10^−1^	1.61
225681_at	CTHRC1	collagen triple helix repeat containing 1	1.31 × 10^−2^	2.82	224396_s_at	ASPN	asporin	2.47 × 10^−2^	4.48
202450_s_at	CTSK	cathepsin K	2.05 × 10^−1^	1.76	219087_at	ASPN	asporin	1.32 × 10^−2^	3.54
213068_at	DPT	dermatopontin	8.16 × 10^−2^	3.67	203477_at	COL15A1	collagen, type XV, alpha 1	2.18 × 10^−1^	2.78
207977_s_at	DPT	dermatopontin	1.24 × 10^−1^	2.85	209082_s_at	COL18A1	collagen, type XVIII, alpha 1	6.56 × 10^−4^	2.99
222885_at	EMCN	endomucin	6.59 × 10^−2^	2.74	209081_s_at	COL18A1	collagen, type XVIII, alpha 1	3.92 × 10^−3^	2.83
227874_at	EMCN	endomucin	2.70 × 10^−1^	1.81	208096_s_at	COL21A1	collagen, type XXI, alpha 1	1.12 × 10^−2^	6.88
219436_s_at	EMCN	endomucin	1.79 × 10^−1^	1.77	232458_at	COL3A1	Collagen, type III, alpha 1	6.64 × 10^−3^	2.66
224374_s_at	EMILIN2	elastin microfibril interfacer 2	6.75 × 10^−2^	1.68	211981_at	COL4A1	collagen, type IV, alpha 1	2.12 × 10^−3^	1.93
203088_at	FBLN5	fibulin 5	2.24 × 10^−2^	1.88	211980_at	COL4A1	collagen, type IV, alpha 1	1.66 × 10^−3^	1.61
203638_s_at	FGFR2	fibroblast growth factor receptor 2	3.21 × 10^−3^	4.52	222073_at	COL4A3	collagen, type IV, alpha 3 (Goodpasture antigen)	2.28 × 10^−2^	1.70
208228_s_at	FGFR2	fibroblast growth factor receptor 2	2.04 × 10^−2^	2.52	229779_at	COL4A4	collagen, type IV, alpha 4	2.85 × 10^−8^	5.32
210187_at	FKBP1A	FK506 binding protein 1A, 12 kDa	8.48 × 10^−2^	1.81	214602_at	COL4A4	collagen, type IV, alpha 4	2.30 × 10^−5^	4.16
226145_s_at	FRAS1	Fraser syndrome 1	6.34 × 10^−2^	2.31	213110_s_at	COL4A5	collagen, type IV, alpha 5	1.05 × 10^−1^	3.62
204983_s_at	GPC4	glypican 4	3.14 × 10^−2^	2.07	52255_s_at	COL5A3	collagen, type V, alpha 3	2.47 × 10^−3^	3.07
204984_at	GPC4	glypican 4	1.57 × 10^−2^	2.02	218975_at	COL5A3	collagen, type V, alpha 3	2.83 × 10^−3^	2.65
235944_at	HMCN1	hemicentin 1	3.52 × 10^−5^	6.28	205832_at	CPA4	carboxypeptidase A4	2.37 × 10^−2^	4.34
203417_at	MFAP2	microfibrillar-associated prot 2	8.08 × 10^−2^	1.70	201116_s_at	CPE	carboxypeptidase E	7.11 × 10^−3^	2.37
204580_at	MMP12	matrix metallopeptidase 12 (macrophage elastase)	2.58 × 10^−1^	2.17	201117_s_at	CPE	carboxypeptidase E	4.90 × 10^−3^	2.10
205828_at	MMP3	matrix metallopeptidase 3	4.79 × 10^−3^	7.70	227138_at	CRTAP	cartilage associated protein	1.31 × 10^−2^	1.63
209596_at	MXRA5	matrix-remodelling associated 5	2.09 × 10^−2^	2.52	201360_at	CST3	cystatin C	6.11 × 10^−2^	1.55
236088_at	NTNG1	netrin G1	2.16 × 10^−2^	2.55	201487_at	CTSC	cathepsin C	6.11 × 10^−5^	2.41
222722_at	OGN	osteoglycin	1.91 × 10^−1^	2.04	225646_at	CTSC	cathepsin C	1.80 × 10^−3^	2.32
228186_s_at	RSPO3	R-spondin 3 homolog (X. laevis)	1.35 × 10^−2^	2.98	225647_s_at	CTSC	cathepsin C	1.37 × 10^−3^	2.25
218638_s_at	SPON2	spondin 2, extracellular matrix prot	3.07 × 10^−2^	2.84	231234_at	CTSC	cathepsin C	5.72 × 10^−3^	2.03
216005_at	TNC	Tenascin C	1.24 × 10^−2^	2.61	202295_s_at	CTSH	cathepsin H	4.00 × 10^−2^	1.85
201645_at	TNC	tenascin C	4.34 × 10^−2^	1.59	209335_at	DCN	decorin	1.02 × 10^−1^	2.05
					211896_s_at	DCN	decorin	2.16 × 10^−1^	1.69
					211813_x_at	DCN	decorin	1.52 × 10^−1^	1.68
					201893_x_at	DCN	decorin	1.77 × 10^−1^	1.56
					1568779_a_at	ECM2	extracellular matrix protein 2	1.76 × 10^−1^	1.71
					206101_at	ECM2	extracellular matrix protein 2	2.50 × 10^−1^	1.57
					201843_s_at	EFEMP1	EGF-containing fibulin-like extracellular matrix protein 1	7.68 × 10^−4^	3.62
					201842_s_at	EFEMP1	EGF-containing fibulin-like extracellular matrix protein 1	4.62 × 10^−4^	2.87
					228421_s_at	EFEMP1	EGF-containing fibulin-like extracellular matrix protein 1	2.23 × 10^−1^	1.61
					226911_at	EGFLAM	EGF-like, fibronectin type III and laminin G domains	3.95 × 10^−3^	4.36
					204834_at	FGL2	fibrinogen-like 2	4.12 × 10^−4^	6.37
					227265_at	FGL2	fibrinogen-like 2	4.28 × 10^−3^	3.56
					202709_at	FMOD	fibromodulin	1.12 × 10^−3^	2.96
					205206_at	KAL1	Kallmann syndrome 1 sequence	9.65 × 10^−4^	7.84
					227048_at	LAMA1	laminin, alpha 1	2.82 × 10^−1^	1.83
					216840_s_at	LAMA2	laminin, alpha 2	5.17 × 10^−3^	2.80
					205116_at	LAMA2	laminin, alpha 2	1.05 × 10^−2^	2.67
					213519_s_at	LAMA2	laminin, alpha 2	1.10 × 10^−2^	2.57
					210150_s_at	LAMA5	laminin, alpha 5	4.86 × 10^−2^	1.57
					211651_s_at	LAMB1	laminin, beta 1	8.27 × 10^−2^	1.54
					200770_s_at	LAMC1	laminin, gamma 1 (formerly LAMB2)	7.61 × 10^−6^	1.91
					200771_at	LAMC1	laminin, gamma 1 (formerly LAMB2)	7.75 × 10^−7^	1.90
					202267_at	LAMC2	laminin, gamma 2	7.41 × 10^−4^	10.0
					200923_at	LGALS3BP LOC100133842	lectin, galactoside-binding, soluble, 3 binding protein similar to lectin, galactoside-binding, soluble, 3 binding protein	1.02 × 10^−2^	2.45
					242767_at	LMCD1	LIM and cysteine-rich domains 1	1.72 × 10^−2^	2.01
					202998_s_at	LOXL2	lysyl oxidase-like 2	4.16 × 10^−3^	1.59
					227145_at	LOXL4	lysyl oxidase-like 4	3.15 × 10^−2^	2.43
					219922_s_at	LTBP3	latent transforming growth factor beta binding protein 3	7.19 × 10^−2^	1.64
					227308_x_at	LTBP3	latent transforming growth factor beta binding protein 3	2.22 × 10^−3^	1.53
					213765_at	MFAP5	microfibrillar associated prot 5	3.51 × 10^−3^	1.61
					213764_s_at	MFAP5	microfibrillar associated prot 5	3.23 × 10^−3^	1.53
					210605_s_at	MFGE8	milk fat globule-EGF factor 8 protein	1.80 × 10^−1^	1.75
					202291_s_at	MGP	matrix Gla protein	3.10 × 10^−4^	7.14
					207847_s_at	MUC1	mucin 1, cell surface associated	1.58 × 10^−1^	2.38
					213693_s_at	MUC1	mucin 1, cell surface associated	2.03 × 10^−2^	2.04
					204114_at	NID2	nidogen 2 (osteonidogen)	2.32 × 10^−3^	2.83
					223315_at	NTN4	netrin 4	1.76 × 10^−4^	14.27
					201860_s_at	PLAT	plasminogen activator, tissue	2.56 × 10^−2^	2.15
					211668_s_at	PLAU	plasminogen activator, urokinase	2.43 × 10^−1^	1.99
					228224_at	PRELP	proline/arginine-rich end leucine-rich repeat protein	2.74 × 10^−2^	3.46
					204223_at	PRELP	proline/arginine-rich end leucine-rich repeat protein	3.27 × 10^−2^	3.27
					205923_at	RELN	reelin	3.58 × 10^−8^	9.15
					202376_at	SERPINA3	serpin peptidase inhibitor, clade A (alpha-1 antiproteinase, antitrypsin), member 3	6.25 × 10^−2^	2.05
					204614_at	SERPINB2	serpin peptidase inhibitor, clade B (ovalbumin), member 2	9.41 × 10^−2^	5.27
					209723_at	SERPINB9	serpin peptidase inhibitor, clade B (ovalbumin), member 9	5.41 × 10^−3^	2.25
					200986_at	SERPING1	serpin peptidase inhibitor, clade G (C1 inhibitor), member 1	2.27 × 10^−1^	1.72
					205352_at	SERPINI1	serpin peptidase inhibitor, clade I (neuroserpin), member 1	1.54 × 10^−3^	2.71
					213493_at	SNED1	sushi, nidogen and EGF-like domains 1	3.24 × 10^−2^	2.47
					213488_at	SNED1	sushi, nidogen and EGF-like domains 1	2.30 × 10^−1^	1.94
					205236_x_at	SOD3	superoxide dismutase 3, extracellular	1.15 × 10^−1^	1.71
					202363_at	SPOCK1	sparc/osteonectin, cwcv and kazal-like domains proteoglycan (testican) 1	5.31 × 10^−3^	2.13
					201858_s_at	SRGN	serglycin	3.34 × 10^−4^	8.11
					201859_at	SRGN	serglycin	3.81 × 10^−4^	4.87
					219552_at	SVEP1	sushi, von Willebrand factor type A, EGF and pentraxin domain containing 1	1.07 × 10^−1^	1.70
					213247_at	SVEP1	sushi, von Willebrand factor type A, EGF and pentraxin domain containing 1	6.04 × 10^−2^	1.70
					226506_at	THSD4	thrombospondin, type I, domain containing 4	6.68 × 10^−4^	3.97
					222835_at	THSD4	thrombospondin, type I, domain containing 4	5.26 × 10^−5^	3.43
					219058_x_at	TINAGL1	tubulointerstitial nephritis antigen-like 1	8.12 × 10^−3^	2.50
					216333_x_at	TNXA TNXB	tenascin XA pseudogene tenascin XB	7.97 × 10^−5^	11.18
					206093_x_at	TNXA TNXB	tenascin XA pseudogene tenascin XB	5.32 × 10^−5^	10.76
					213451_x_at	TNXA TNXB	tenascin XA pseudogene tenascin XB	1.65 × 10^−4^	9.71
					208609_s_at	TNXB	tenascin XB	7.07 × 10^−5^	8.99
**Focal Adhesion Points**									
**Probe Set ID**	**Gene Symbol**	**Gene Title**	**adj-pval**	**FC**	**Probe Set ID**	**Gene Symbol**	**Gene Title**	**adj-pval**	**FC**
205730_s_at	ABLIM3	actin binding LIM protein family, member 3	1.06 × 10^−1^	1.53	200965_s_at	ABLIM1	actin binding LIM protein 1	2.02 × 10^−5^	4.17
213497_at	ABTB2	ankyrin repeat and BTB (POZ) domain containing 2	6.59 × 10^−2^	1.61	205882_x_at	ADD3	adducin 3 (gamma)	6.45 × 10^−3^	1.59
205268_s_at	ADD2	adducin 2 (beta)	8.16 × 10^−4^	7.50	201752_s_at	ADD3	adducin 3 (gamma)	9.71 × 10^−3^	1.56
205771_s_at	AKAP7	A kinase (PRKA) anchor prot 7	1.03 × 10^−1^	1.58	227529_s_at	AKAP12	A kinase (PRKA) anchor prot 12	6.81 × 10^−3^	8.03
205257_s_at	AMPH	amphiphysin	1.45 × 10^−7^	6.02	227530_at	AKAP12	A kinase (PRKA) anchor prot 12	4.43 × 10^−3^	6.10
1552619_a_at	ANLN	anillin, actin binding protein	3.48 × 10^−2^	2.47	210517_s_at	AKAP12	A kinase (PRKA) anchor prot 12	6.53 × 10^−3^	4.66
222608_s_at	ANLN	anillin, actin binding protein	4.60 × 10^−2^	2.25	202920_at	ANK2	ankyrin 2, neuronal	8.40 × 10^−3^	2.34
203526_s_at	APC	adenomatous polyposis coli	3.34 × 10^−3^	1.60	206385_s_at	ANK3	ankyrin 3, node of Ranvier (ankyrin G)	3.69 × 10^−3^	3.16
204492_at	ARHGAP11A	Rho GTPase activating protein 11A	3.11 × 10^−2^	2.12	227337_at	ANKRD37	ankyrin repeat domain 37	4.35 × 10^−6^	6.04
37577_at	ARHGAP19	Rho GTPase activating protein 19	9.72 × 10^−4^	1.72	204671_s_at	ANKRD6	ankyrin repeat domain 6	3.20 × 10^−2^	2.12
206298_at	ARHGAP22	Rho GTPase activating protein 22	8.57 × 10^−3^	1.58	204672_s_at	ANKRD6	ankyrin repeat domain 6	5.69 × 10^−2^	1.96
201288_at	ARHGDIB	Rho GDP dissociation inhibitor (GDI) beta	3.85 × 10^−2^	1.54	228368_at	ARHGAP20	Rho GTPase activating prot 20	1.15 × 10^−4^	5.72
204092_s_at	AURKA	aurora kinase A	2.07 × 10^−2^	2.88	227911_at	ARHGAP28	Rho GTPase activating prot 28	1.07 × 10^−3^	2.38
208079_s_at	AURKA	aurora kinase A	3.80 × 10^−2^	2.45	206167_s_at	ARHGAP6	Rho GTPase activating prot 6	1.09 × 10^−1^	1.65
209464_at	AURKB	aurora kinase B	1.04 × 10^−2^	2.92	205109_s_at	ARHGEF4	Rho guanine nucleotide exchange factor (GEF) 4	1.49 × 10^−1^	1.53
205294_at	BAIAP2	BAI1-associated protein 2	2.67 × 10^−3^	1.51	201615_x_at	CALD1	caldesmon 1	1.61 × 10^−1^	1.79
210334_x_at	BIRC5	baculoviral IAP repeat-containing 5	1.01 × 10^−2^	2.69	201616_s_at	CALD1	caldesmon 1	1.89 × 10^−2^	1.53
202094_at	BIRC5	baculoviral IAP repeat-containing 5	4.10 × 10^−2^	2.63	236473_at	CC2D2A	coiled-coil and C2 domain containing 2A	2.18 × 10^−3^	2.56
202095_s_at	BIRC5	baculoviral IAP repeat-containing 5	1.72 × 10^−2^	2.47	203139_at	DAPK1	death-associated protein kinase 1	2.66 × 10^−2^	3.97
220935_s_at	CDK5RAP2	CDK5 regulatory subunit associated protein 2	1.91 × 10^−7^	1.78	229800_at	DCLK1	Doublecortin-like kinase 1	1.78 × 10^−1^	1.99
204962_s_at	CENPA	centromere protein A	3.83 × 10^−2^	2.88	217208_s_at	DLG1	discs, large homolog 1 (Drosophila)	6.19 × 10^−3^	2.32
210821_x_at	CENPA	centromere protein A	4.21 × 10^−3^	1.98	202515_at	DLG1	discs, large homolog 1 (Drosophila)	1.59 × 10^−3^	1.72
205046_at	CENPE	centromere protein E, 312 kDa	7.08 × 10^−2^	2.65	202514_at	DLG1	discs, large homolog 1 (Drosophila)	6.69 × 10^−3^	1.72
209172_s_at	CENPF	centromere protein F, 350/400 ka (mitosin)	1.33 × 10^−2^	2.98	230229_at	DLG1	Discs, large homolog 1 (Drosophila)	1.49 × 10^−1^	1.63
207828_s_at	CENPF	centromere protein F, 350/400 ka (mitosin)	2.29 × 10^−2^	2.94	202516_s_at	DLG1	discs, large homolog 1 (Drosophila)	5.92 × 10^−2^	1.60
231772_x_at	CENPH	centromere protein H	2.74 × 10^−2^	1.82	203881_s_at	DMD	dystrophin	1.06 × 10^−4^	5.02
214804_at	CENPI	centromere protein I	5.29 × 10^−2^	1.96	208086_s_at	DMD	dystrophin	2.36 × 10^−2^	1.58
207590_s_at	CENPI	centromere protein I	1.62 × 10^−2^	1.88	227081_at	DNALI1	dynein, axonemal, light intermediate chain 1	2.64 × 10^−2^	1.64
223513_at	CENPJ	centromere protein J	2.83 × 10^−2^	1.69	226875_at	DOCK11	dedicator of cytokinesis 11	4.24 × 10^−4^	1.78
222848_at	CENPK	centromere protein K	1.00 × 10^−1^	1.94	1554863_s_at	DOK5	docking protein 5	1.93 × 10^−2^	1.59
1554271_a_at	CENPL	centromere protein L	1.69 × 10^−1^	1.54	214844_s_at	DOK5	docking protein 5	2.98 × 10^−3^	1.52
218741_at	CENPM	centromere protein M	1.86 × 10^−2^	2.36	220161_s_at	EPB41L4B	erythrocyte membrane protein band 4.1 like 4B	5.64 × 10^−2^	2.33
219555_s_at	CENPN	centromere protein N	9.25 × 10^−3^	1.89	209829_at	FAM65B	family with sequence similarity 65, member B	3.97 × 10^−2^	2.58
222118_at	CENPN	centromere protein N	1.24 × 10^−1^	1.84	206707_x_at	FAM65B	family with sequence similarity 65, member B	3.02 × 10^−2^	2.20
228559_at	CENPN	centromere protein N	8.63 × 10^−2^	1.74	226129_at	FAM83H	family with sequence similarity 83, member H	2.04 × 10^−2^	1.75
226118_at	CENPO	centromere protein O	6.22 × 10^−2^	1.78	227948_at	FGD4	FYVE, RhoGEF and PH domain containing 4	9.89 × 10^−4^	3.94
219294_at	CENPQ	centromere protein Q	5.50 × 10^−2^	1.56	230559_x_at	FGD4	FYVE, RhoGEF and PH domain containing 4	3.65 × 10^−3^	2.66
205642_at	CEP110	centrosomal protein 110 kDa	6.59 × 10^−3^	1.87	225167_at	FRMD4A	FERM domain containing 4A	1.45 × 10^−2^	2.04
239413_at	CEP152	centrosomal protein 152 kDa	3.34 × 10^−3^	1.71	225163_at	FRMD4A	FERM domain containing 4A	8.39 × 10^−3^	1.98
218542_at	CEP55	centrosomal protein 55 kDa	2.63 × 10^−2^	2.44	225168_at	FRMD4A	FERM domain containing 4A	2.22 × 10^−2^	1.76
206324_s_at	DAPK2	death-associated protein kinase 2	7.47 × 10^−2^	1.73	1560031_at	FRMD4A	FERM domain containing 4A	1.07 × 10^−3^	1.71
227666_at	DCLK2	doublecortin-like kinase 2	7.38 × 10^−2^	1.52	208476_s_at	FRMD4A	FERM domain containing 4A	7.85 × 10^−3^	1.69
207147_at	DLX2	distal-less homeobox 2	1.94 × 10^−2^	6.12	1554034_a_at	FRMD4A	FERM domain containing 4A	2.32 × 10^−1^	1.57
215116_s_at	DNM1	dynamin 1	2.03 × 10^−4^	3.83	239290_at	FRMPD4	FERM and PDZ domain containing 4	1.74 × 10^−1^	1.56
219279_at	DOCK10	dedicator of cytokinesis 10	2.60 × 10^−2^	1.66	203037_s_at	MTSS1	metastasis suppressor 1	2.31 × 10^−3^	4.32
213160_at	DOCK2	dedicator of cytokinesis 2	1.12 × 10^−4^	1.76	212096_s_at	MTUS1	mitochondrial tumor supp 1	1.02 × 10^−1^	2.47
205003_at	DOCK4	dedicator of cytokinesis 4	1.44 × 10^−1^	1.54	212095_s_at	MTUS1	mitochondrial tumor supp 1	1.08 × 10^−1^	1.74
206710_s_at	EPB41L3	erythrocyte membrane protein band 4.1-like 3	1.31 × 10^−2^	3.52	228098_s_at	MYLIP	myosin regulatory light chain interacting protein	5.16 × 10^−2^	1.57
212681_at	EPB41L3	erythrocyte membrane protein band 4.1-like 3	1.38 × 10^−2^	3.22	220319_s_at	MYLIP	myosin regulatory light chain interacting protein	4.55 × 10^−2^	1.50
211776_s_at	EPB41L3	erythrocyte membrane protein band 4.1-like 3	1.48 × 10^−2^	3.20	237206_at	MYOCD	myocardin	8.62 × 10^−3^	3.85
218980_at	FHOD3	formin homology 2 domain containing 3	7.93 × 10^−3^	3.13	213782_s_at	MYOZ2	myozenin 2	8.68 × 10^−2^	2.16
238621_at	FMN1	formin 1	6.44 × 10^−3^	2.47	207148_x_at	MYOZ2	myozenin 2	8.91 × 10^−2^	2.07
1555471_a_at	FMN2	formin 2	2.05 × 10^−2^	1.85	219073_s_at	OSBPL10	oxysterol binding protein-like 10	3.31 × 10^−2^	2.11
223618_at	FMN2	formin 2	2.05 × 10^−2^	1.82	209621_s_at	PDLIM3	PDZ and LIM domain 3	8.38 × 10^−2^	3.31
215017_s_at	FNBP1L	formin binding protein 1-like	3.68 × 10^−3^	1.52	213684_s_at	PDLIM5	PDZ and LIM domain 5	6.28 × 10^−3^	1.87
230645_at	FRMD3	FERM domain containing 3	2.69 × 10^−1^	1.63	221994_at	PDLIM5	PDZ and LIM domain 5	2.46 × 10^−3^	1.81
230831_at	FRMD5	FERM domain containing 5	1.02 × 10^−2^	2.87	203242_s_at	PDLIM5	PDZ and LIM domain 5	1.86 × 10^−3^	1.68
238756_at	GAS2L3	Growth arrest-specific 2 like 3	1.22 × 10^−2^	2.33	216804_s_at	PDLIM5	PDZ and LIM domain 5	4.90 × 10^−3^	1.60
235709_at	GAS2L3	growth arrest-specific 2 like 3	2.92 × 10^−2^	1.81	207717_s_at	PKP2	plakophilin 2	2.96 × 10^−2^	3.09
226308_at	HAUS8	HAUS augmin-like complex, subunit 8	3.75 × 10^−2^	1.71	201927_s_at	PKP4	plakophilin 4	2.90 × 10^−1^	1.68
226364_at	HIP1	Huntingtin interacting protein 1	1.05 × 10^−3^	2.73	227148_at	PLEKHH2	pleckstrin homology domain containing, family H member 2	3.16 × 10^−3^	3.05
205425_at	HIP1	huntingtin interacting protein 1	9.54 × 10^−3^	2.66	203407_at	PPL	periplakin	7.99 × 10^−3^	3.20
218934_s_at	HSPB7	heat shock 27 kDa protein family, member 7 (cardiovascular)	7.51 × 10^−2^	2.16	226627_at	SEPT8	septin 8	1.20 × 10^−1^	1.74
227750_at	KALRN	kalirin, RhoGEF kinase	8.61 × 10^−3^	1.53	226438_at	SNTB1	syntrophin, beta 1 (dystrophin-associated protein A1, 59 kDa, basic component 1)	1.03 × 10^−2^	1.84
229125_at	KANK4	KN motif and ankyrin repeat domains 4	1.76 × 10^−2^	3.51	214708_at	SNTB1	syntrophin, beta 1 (dystrophin-associated protein A1, 59 kDa, basic component 1)	3.83 × 10^−2^	1.53
204444_at	KIF11	kinesin family member 11	4.00 × 10^−2^	2.43	227179_at	STAU2	staufen, RNA binding protein, homolog 2 (Drosophila)	1.58 × 10^−2^	1.89
236641_at	KIF14	kinesin family member 14	1.15 × 10^−2^	3.50	212565_at	STK38L	serine/threonine kinase 38 like	5.74 × 10^−5^	1.94
206364_at	KIF14	kinesin family member 14	5.29 × 10^−2^	2.87	212572_at	STK38L	serine/threonine kinase 38 like	5.09 × 10^−3^	1.53
219306_at	KIF15	kinesin family member 15	1.87 × 10^−2^	2.65	202796_at	SYNPO	synaptopodin	7.05 × 10^−2^	2.18
221258_s_at	KIF18A	kinesin family member 18A	1.25 × 10^−2^	2.79	227662_at	SYNPO2	synaptopodin 2	1.29 × 10^−1^	3.30
222039_at	KIF18B	kinesin family member 18B	6.34 × 10^−2^	2.25	213135_at	TIAM1	T-cell lymphoma invasion and metastasis 1	1.12 × 10^−1^	1.65
218755_at	KIF20A	kinesin family member 20A	1.17 × 10^−2^	2.81	209904_at	TNNC1	troponin C type 1 (slow)	6.34 × 10^−2^	2.76
205235_s_at	KIF20B	kinesin family member 20B	1.83 × 10^−2^	1.95	215389_s_at	TNNT2	troponin T type 2 (cardiac)	5.06 × 10^−2^	3.22
216969_s_at	KIF22	kinesin family member 22	9.75 × 10^−2^	1.92	210276_s_at	TRIOBP	TRIO and F-actin binding prot	6.96 × 10^−2^	1.52
202183_s_at	KIF22	kinesin family member 22	6.19 × 10^−3^	1.65	223279_s_at	UACA	uveal autoantigen with coiled-coil domains and ankyrin repeats	6.84 × 10^−3^	1.79
204709_s_at	KIF23	kinesin family member 23	3.68 × 10^−2^	2.55	238868_at	UACA	uveal autoantigen with coiled-coil domains and ankyrin repeats	1.37 × 10^−1^	1.66
244427_at	KIF23	Kinesin family member 23	2.68 × 10^−3^	1.76					
209408_at	KIF2C	kinesin family member 2C	3.86 × 10^−2^	2.95					
211519_s_at	KIF2C	kinesin family member 2C	2.04 × 10^−2^	2.80					
218355_at	KIF4A	kinesin family member 4A	3.29 × 10^−2^	2.60					
209680_s_at	KIFC1	kinesin family member C1	1.72 × 10^−2^	2.43					
206316_s_at	KNTC1	kinetochore associated 1	1.88 × 10^−2^	1.86					
224823_at	MYLK	myosin light chain kinase	1.91 × 10^−1^	1.72					
236718_at	MYO10	myosin X	1.05 × 10^−3^	1.86					
244350_at	MYO10	myosin X	1.45 × 10^−2^	1.70					
241966_at	MYO5A	myosin VA (heavy chain 12, myoxin)	2.21 × 10^−2^	1.51					
201774_s_at	NCAPD2	non-SMC condensin I complex, subunit D2	1.30 × 10^−1^	1.57					
212789_at	NCAPD3	non-SMC condensin II complex, subunit D3	5.55 × 10^−2^	1.56					
218663_at	NCAPG	non-SMC condensin I complex, subunit G	1.03 × 10^−1^	2.23					
218662_s_at	NCAPG	non-SMC condensin I complex, subunit G	8.24 × 10^−2^	2.11					
219588_s_at	NCAPG2	non-SMC condensin II complex, subunit G2	2.03 × 10^−2^	1.86					
212949_at	NCAPH	non-SMC condensin I complex, subunit H	3.01 × 10^−2^	2.58					
204641_at	NEK2	NIMA (never in mitosis gene a)-related kinase 2	2.34 × 10^−2^	2.97					
211080_s_at	NEK2	NIMA (never in mitosis gene a)-related kinase 2	4.89 × 10^−3^	2.64					
223381_at	NUF2	NUF2, NDC80 kinetochore complex component, homolog (S. cerevisiae)	8.44 × 10^−2^	2.67					
219978_s_at	NUSAP1	nucleolar and spindle associated protein 1	1.39 × 10^−1^	2.48					
218039_at	NUSAP1	nucleolar and spindle associated protein 1	2.98 × 10^−2^	2.42					
204972_at	OAS2	2’-5’-oligoadenylate synthetase 2, 69/71 kDa	2.83 × 10^−1^	1.68					
209626_s_at	OSBPL3	oxysterol binding protein-like 3	5.77 × 10^−2^	1.67					
238575_at	OSBPL6	oxysterol binding protein-like 6	1.17 × 10^−2^	2.13					
223805_at	OSBPL6	oxysterol binding protein-like 6	9.15 × 10^−3^	2.09					
218644_at	PLEK2	pleckstrin 2	4.94 × 10^−3^	2.63					
218009_s_at	PRC1	protein regulator of cytokinesis 1	2.03 × 10^−2^	2.37					
222077_s_at	RACGAP1	Rac GTPase activating protein 1	1.49 × 10^−2^	1.99					
219263_at	RNF128	ring finger protein 128	2.50 × 10^−2^	3.03					
230730_at	SGCD	sarcoglycan, delta (35 kDa dystrophin-associated glycoprotein)	2.17 × 10^−2^	3.65					
213543_at	SGCD	sarcoglycan, delta (35 kDa dystrophin-associated glycoprotein)	2.12 × 10^−2^	3.57					
228602_at	SGCD	sarcoglycan, delta (35 kDa dystrophin-associated glycoprotein)	6.41 × 10^−2^	3.55					
214492_at	SGCD	sarcoglycan, delta (35 kDa dystrophin-associated glycoprotein)	5.41 × 10^−3^	3.18					
210329_s_at	SGCD	sarcoglycan, delta (35 kDa dystrophin-associated glycoprotein)	8.64 × 10^−3^	2.72					
210330_at	SGCD	sarcoglycan, delta (35 kDa dystrophin-associated glycoprotein)	3.47 × 10^−2^	2.43					
207302_at	SGCG	sarcoglycan, gamma (35 kDa dystrophin-associated glycoprotein)	1.42 × 10^−^1	3.09					
217678_at	SLC7A11	solute carrier family 7, (cationic amino acid transporter, y+ system) member 11	7.43 × 10^−2^	1.53					
209921_at	SLC7A11	solute carrier family 7, (cationic amino acid transporter, y+ system) member 11	3.18 × 10^−2^	1.53					
1556583_a_at	SLC8A1	solute carrier family 8 (sodium/calcium exchanger), member 1	1.96 × 10^−1^	1.85					
241752_at	SLC8A1	solute carrier family 8 (sodium/calcium exchanger), member 1	2.94 × 10^−1^	1.61					
200783_s_at	STMN1	stathmin 1	1.16 × 10^−2^	2.14					
222557_at	STMN3	stathmin-like 3	1.48 × 10^−2^	1.83					
212703_at	TLN2	talin 2	5.18 × 10^−4^	1.84					
206117_at	TPM1	tropomyosin 1 (alpha)	5.66 × 10^−3^	2.26					
210052_s_at	TPX2	TPX2, microtubule-associated, homolog (Xenopus laevis)	1.78 × 10^−2^	2.56					
1555938_x_at	VIM	vimentin	2.81 × 10^−2^	2.00					
202663_at	WIPF1	WAS/WASL interacting protein family, member 1	6.39 × 10^−3^	1.64					
202664_at	WIPF1	WAS/WASL interacting protein family, member 1	5.61 × 10^−4^	1.58					
202665_s_at	WIPF1	WAS/WASL interacting protein family, member 1	2.97 × 10^−3^	1.51					
**Cytoskeleton**									
**Probe Set ID**	**Gene Symbol**	**Gene Title**	**adj-pval**	**FC**	**Probe Set ID**	**Gene Symbol**	**Gene Title**	**adj-pval**	**FC**
205132_at	ACTC1	actin, alpha, cardiac muscle 1	1.80 × 10^−3^	4.22	203563_at	AFAP1	actin filament associated protein 1	2.27 × 10^−3^	1.90
230925_at	APBB1IP	amyloid beta (A4) precursor protein-binding, family B, member 1 interacting protein	1.66 × 10^−2^	2.50	206488_s_at	CD36	CD36 molecule (thrombospondin receptor)	1.46 × 10^−8^	20.69
226292_at	CAPN5	calpain 5	2.20 × 10^−4^	1.51	209555_s_at	CD36	CD36 molecule (thrombospondin receptor)	3.58 × 10^−8^	19.05
217523_at	CD44	CD44 molecule (Indian blood group)	1.89 × 10^−2^	1.64	228766_at	CD36	CD36 molecule (thrombospondin receptor)	2.97 × 10^−6^	11.94
220115_s_at	CDH10	cadherin 10, type 2 (T2-cadherin)	1.94 × 10^−1^	1.90	201005_at	CD9	CD9 molecule	7.82 × 10^−4^	2.58
207030_s_at	CSRP2	cysteine and glycine-rich protein 2	1.93 × 10^−2^	1.74	201131_s_at	CDH1	cadherin 1, type 1, E-cadherin (epithelial)	4.31 × 10^−2^	2.54
211126_s_at	CSRP2	cysteine and glycine-rich protein 2	2.34 × 10^−2^	1.71	204726_at	CDH13	cadherin 13, H-cadherin (heart)	3.20 × 10^−2^	2.60
214724_at	DIXDC1	DIX domain containing 1	1.14 × 10^−2^	1.53	203256_at	CDH3	cadherin 3, type 1, P-cadherin (placental)	3.21 × 10^−2^	1.84
202668_at	EFNB2	ephrin-B2	1.85 × 10^−1^	3.24	200621_at	CSRP1	cysteine and glycine-rich protein 1	1.70 × 10^−2^	1.56
205031_at	EFNB3	ephrin-B3	2.95 × 10^−10^	2.47	203716_s_at	DPP4	dipeptidyl-peptidase 4	4.05 × 10^−2^	1.93
1555480_a_at	FBLIM1	filamin binding LIM protein 1	1.07 × 10^−2^	1.89	211478_s_at	DPP4	dipeptidyl-peptidase 4	2.67 × 10^−1^	1.80
1554795_a_at	FBLIM1	filamin binding LIM protein 1	2.26 × 10^−2^	1.61	203717_at	DPP4	dipeptidyl-peptidase 4	1.08 × 10^−1^	1.67
225258_at	FBLIM1	filamin binding LIM protein 1	2.87 × 10^−3^	1.56	227955_s_at	EFNA5	ephrin-A5	4.68 × 10^−2^	1.94
204379_s_at	FGFR3	fibroblast growth factor receptor 3	9.77 × 10^−2^	2.01	214036_at	EFNA5	ephrin-A5	1.32 × 10^−1^	1.53
242592_at	GPR137C	G protein-coupled receptor 137C	1.50 × 10^−2^	2.18	201983_s_at	EGFR	epidermal growth factor receptor (erythroblastic leukemia viral (v-erb-b) oncogene homolog, avian)	1.75 × 10^−3^	1.82
235961_at	GPR161	G protein-coupled receptor 161	4.34 × 10^−4^	1.56	201809_s_at	ENG	endoglin	2.31 × 10^−3^	2.06
230369_at	GPR161	G protein-coupled receptor 161	3.44 × 10^−2^	1.53	201539_s_at	FHL1	four and a half LIM domains 1	8.39 × 10^−3^	6.09
229055_at	GPR68	G protein-coupled receptor 68	7.83 × 10^−3^	1.70	214505_s_at	FHL1	four and a half LIM domains 1	6.48 × 10^−3^	5.76
234303_s_at	GPR85	G protein-coupled receptor 85	7.25 × 10^−2^	2.04	210299_s_at	FHL1	four and a half LIM domains 1	1.55 × 10^−3^	5.39
203632_s_at	GPRC5B	G protein-coupled receptor, family C, group 5, member B	1.76 × 10^−1^	1.92	210298_x_at	FHL1	four and a half LIM domains 1	6.70 × 10^−3^	5.18
222899_at	ITGA11	integrin, alpha 11	1.21 × 10^−2^	1.59	201540_at	FHL1	four and a half LIM domains 1	4.21 × 10^−4^	3.20
227314_at	ITGA2	integrin, alpha 2 (CD49B, alpha 2 subunit of VLA-2 receptor)	5.68 × 10^−2^	2.53	222853_at	FLRT3	fibronectin leucine rich transmembrane protein 3	7.40 × 10^−4^	3.36
205032_at	ITGA2	integrin, alpha 2 (CD49B, alpha 2 subunit of VLA-2 receptor)	3.78 × 10^−2^	2.41	219250_s_at	FLRT3	fibronectin leucine rich transmembrane protein 3	4.52 × 10^−2^	2.12
228080_at	LAYN	layilin	4.84 × 10^−3^	2.59	212950_at	GPR116	G protein-coupled receptor 116	1.98 × 10^−1^	3.52
216250_s_at	LPXN	leupaxin	2.03 × 10^−5^	3.08	213094_at	GPR126	G protein-coupled receptor 126	9.65 × 10^−4^	5.14
210869_s_at	MCAM	melanoma cell adhesion molecule	1.03 × 10^−6^	7.67	232267_at	GPR133	G protein-coupled receptor 133	1.46 × 10^−2^	2.44
209087_x_at	MCAM	melanoma cell adhesion molecule	1.08 × 10^−6^	7.38	228949_at	GPR177	G protein-coupled receptor 177	3.16 × 10^−4^	2.71
211340_s_at	MCAM	melanoma cell adhesion molecule	1.07 × 10^−6^	6.81	228950_s_at	GPR177	G protein-coupled receptor 177	3.49 × 10^−3^	2.63
209086_x_at	MCAM	melanoma cell adhesion molecule	2.85 × 10^−8^	5.09	221958_s_at	GPR177	G protein-coupled receptor 177	1.52 × 10^−3^	2.50
203062_s_at	MDC1	mediator of DNA damage checkpoint 1	3.87 × 10^−3^	1.58	229105_at	GPR39	G protein-coupled receptor 39	2.66 × 10^−2^	1.90
212843_at	NCAM1	neural cell adhesion molecule 1	8.99 × 10^−7^	4.58	212070_at	GPR56	G protein-coupled receptor 56	2.12 × 10^−2^	1.78
227394_at	NCAM1	neural cell adhesion molecule 1	1.26 × 10^−6^	3.16	203108_at	GPRC5A	G protein-coupled receptor, family C, group 5, member A	2.08 × 10^−3^	8.54
213438_at	NFASC	neurofascin homolog (chicken)	3.12 × 10^−2^	2.57	202638_s_at	ICAM1	intercellular adhesion molecule 1	1.31 × 10^−1^	2.21
230242_at	NFASC	neurofascin homolog (chicken)	5.17 × 10^−3^	2.19	202637_s_at	ICAM1	intercellular adhesion molecule 1	7.04 × 10^−2^	1.70
243645_at	NFASC	neurofascin homolog (chicken)	2.12 × 10^−3^	2.03	205885_s_at	ITGA4	integrin, alpha 4 (antigen CD49D, alpha 4 subunit of VLA-4 receptor)	2.52 × 10^−1^	1.71
219773_at	NOX4	NADPH oxidase 4	8.60 × 10^−2^	1.80	216331_at	ITGA7	integrin, alpha 7	1.01 × 10^−2^	3.76
37966_at	PARVB	parvin, beta	4.78 × 10^−10^	2.88	209663_s_at	ITGA7	integrin, alpha 7	4.49 × 10^−2^	3.70
204629_at	PARVB	parvin, beta	2.58 × 10^−8^	2.28	204990_s_at	ITGB4	integrin, beta 4	8.14 × 10^−2^	1.67
37965_at	PARVB	parvin, beta	7.26 × 10^−5^	1.98	226189_at	ITGB8	integrin, beta 8	2.19 × 10^−2^	1.69
216253_s_at	PARVB	parvin, beta	4.90 × 10^−3^	1.80	220765_s_at	LIMS2	LIM and senescent cell antigen-like domains 2	1.80 × 10^−2^	1.80
225977_at	PCDH18	protocadherin 18	4.98 × 10^−3^	2.18	226974_at	NEDD4L	neural precursor cell expressed, developmentally down-regulated 4-like	4.84 × 10^−2^	2.02
225975_at	PCDH18	protocadherin 18	1.29 × 10^−2^	1.76	212448_at	NEDD4L	neural precursor cell expressed, developmentally down-regulated 4-like	9.48 × 10^−2^	1.78
207011_s_at	PTK7	PTK7 protein tyrosine kinase 7	3.74 × 10^−3^	2.23	212445_s_at	NEDD4L	neural precursor cell expressed, developmentally down-regulated 4-like	1.04 × 10^−1^	1.76
1555324_at	PTK7	PTK7 protein tyrosine kinase 7	5.32 × 10^−3^	1.78	202150_s_at	NEDD9	neural precursor cell expressed, developmentally down-regulated 9	3.21 × 10^−3^	1.97
207419_s_at	RAC2	ras-related C3 botulinum toxin substrate 2 (rho family, small GTP binding protein Rac2)	1.39 × 10^−2^	2.37	202149_at	NEDD9	neural precursor cell expressed, developmentally down-regulated 9	3.93 × 10^−4^	1.90
213603_s_at	RAC2	ras-related C3 botulinum toxin substrate 2 (rho family, small GTP binding protein Rac2)	8.95 × 10^−3^	2.13	228635_at	PCDH10	protocadherin 10	2.55 × 10^−3^	3.91
223168_at	RHOU	ras homolog gene family, member U	1.13 × 10^−5^	3.13	223435_s_at	PCDHA1 PCDHA10 PCDHA11 PCDHA12 PCDHA13 PCDHA2 PCDHA3 PCDHA4 PCDHA5 PCDHA6 PCDHA7 PCDHA8 PCDHA9 PCDHAC1 PCDHAC2	protocadherin alpha 1 protocadherin alpha 10 protocadherin alpha 11 protocadherin alpha 12 protocadherin alpha 13 protocadherin alpha 2 protocadherin alpha 3 protocadherin alpha 4 protocadherin alpha 5 protocadherin alpha 6 protocadherin alpha 7 protocadherin alpha 8 protocadherin alpha 9 protocadherin alpha C, 1 protocadherin alpha C, 2	2.40 × 10^−3^	2.23
201286_at	SDC1	syndecan 1	7.78 × 10^−3^	3.11	202565_s_at	SVIL	supervillin	5.25 × 10^−3^	3.45
201287_s_at	SDC1	syndecan 1	3.47 × 10^−3^	3.04	202566_s_at	SVIL	supervillin	5.31 × 10^−2^	2.32
202898_at	SDC3	syndecan 3	2.98 × 10^−2^	1.66	206702_at	TEK	TEK tyrosine kinase, endothelial	4.91 × 10^−4^	3.67
218087_s_at	SORBS1	sorbin and SH3 domain containing 1	1.05 × 10^−2^	4.48					
222513_s_at	SORBS1	sorbin and SH3 domain containing 1	3.04 × 10^−2^	2.95					
208850_s_at	THY1	Thy-1 cell surface antigen	2.80 × 10^−1^	1.91					
213869_x_at	THY1	Thy-1 cell surface antigen	1.38 × 10^−1^	1.77					
208851_s_at	THY1	Thy-1 cell surface antigen	2.50 × 10^−1^	1.70					
217853_at	TNS3	tensin 3	5.51 × 10^−5^	2.61					
217979_at	TSPAN13	tetraspanin 13	1.08 × 10^−4^	4.67					
227307_at	TSPAN18	Tetraspanin 18	2.78 × 10^−4^	3.95					
227236_at	TSPAN2	tetraspanin 2	1.85 × 10^−1^	2.19					
214606_at	TSPAN2	tetraspanin 2	1.19 × 10^−1^	1.83					
**LINC Complexes**									
**Probe Set ID**	**Gene Symbol**	**Gene Title**	**adj-pval**	**FC**	**Probe Set ID**	**Gene Symbol**	**Gene Title**	**adj-pval**	**FC**
203145_at	SPAG5	sperm associated antigen 5	2.63 × 10^−2^	2.48	219888_at	SPAG4	sperm associated antigen 4	8.03 × 10^−2^	1.87
**Nucleoskeleton**									
**Probe Set ID**	**Gene Symbol**	**Gene Title**	**adj-pval**	**FC**	**Probe Set ID**	**Gene Symbol**	**Gene Title**	**adj-pval**	**FC**
205436_s_at	H2AFX	H2A histone family, member X	1.88 × 10^−2^	1.71	215071_s_at	HIST1H2AC	histone cluster 1, H2ac	2.13 × 10^−2^	1.70
214463_x_at	HIST1H4J	histone cluster 1, H4j	1.24 × 10^−2^	1.53	214455_at	HIST1H2BC	histone cluster 1, H2bc	1.34 × 10^−2^	1.71
201795_at	LBR	lamin B receptor	8.68 × 10^−4^	1.88	236193_at	HIST1H2BC	histone cluster 1, H2bc	1.57 × 10^−2^	1.60
203276_at	LMNB1	lamin B1	8.33 × 10^−2^	2.42	209911_x_at	HIST1H2BD	histone cluster 1, H2bd	1.63 × 10^−2^	1.63
209753_s_at	TMPO	thymopoietin	5.06 × 10^−3^	1.98	208527_x_at	HIST1H2BE	histone cluster 1, H2be	4.22 × 10^−3^	1.54
224944_at	TMPO	thymopoietin	5.13 × 10^−3^	1.84	232035_at	HIST1H4B	Histone cluster 1, H4b	5.60 × 10^−3^	1.96
209754_s_at	TMPO	thymopoietin	2.51 × 10^−2^	1.83	208180_s_at	HIST1H4B	Histone cluster 1, H4b	1.34 × 10^−1^	1.59
203432_at	TMPO	thymopoietin	1.02 × 10^−1^	1.64					
**Secreted Factors**									
**Probe Set ID**	**Gene Symbol**	**Gene Title**	**adj-pval**	**FC**	**Probe Set ID**	**Gene Symbol**	**Gene Title**	**adj-pval**	**FC**
205608_s_at	ANGPT1	angiopoietin 1	1.27 × 10^−4^	3.04	231773_at	ANGPTL1	angiopoietin-like 1	3.01 × 10^−2^	2.12
205609_at	ANGPT1	angiopoietin 1	7.38 × 10^−6^	2.97	224339_s_at	ANGPTL1	angiopoietin-like 1	7.59 × 10^−2^	1.71
213001_at	ANGPTL2	angiopoietin-like 2	1.03 × 10^−1^	1.50	239183_at	ANGPTL1	angiopoietin-like 1	8.51 × 10^−2^	1.50
220988_s_at	C1QTNF3	C1q and tumor necrosis factor related protein 3	9.99 × 10^−2^	1.78	221009_s_at	ANGPTL4	angiopoietin-like 4	6.59 × 10^−3^	2.88
1405_i_at	CCL5	chemokine (C-C motif) ligand 5	1.12 × 10^−1^	1.56	223333_s_at	ANGPTL4	angiopoietin-like 4	1.80 × 10^−1^	2.11
203666_at	CXCL12	chemokine (C-X-C motif) ligand 12 (stromal cell-derived factor 1)	6.11 × 10^−3^	2.98	209546_s_at	APOL1	apolipoprotein L, 1	1.42 × 10^−1^	1.78
209687_at	CXCL12	chemokine (C-X-C motif) ligand 12 (stromal cell-derived factor 1)	1.99 × 10^−2^	2.24	221087_s_at	APOL3	apolipoprotein L, 3	6.19 × 10^−2^	1.83
222484_s_at	CXCL14	chemokine (C-X-C motif) ligand 14	5.41 × 10^−4^	7.47	205239_at	AREG	amphiregulin	1.14 × 10^−1^	1.75
218002_s_at	CXCL14	chemokine (C-X-C motif) ligand 14	1.22 × 10^−3^	6.46	202701_at	BMP1	bone morphogenetic protein 1	5.22 × 10^−2^	1.54
204602_at	DKK1	dickkopf homolog 1 (Xenopus laevis)	2.42 × 10^−2^	2.04	205289_at	BMP2	bone morphogenetic protein 2	2.65 × 10^−2^	2.54
219908_at	DKK2	dickkopf homolog 2 (Xenopus laevis)	7.38 × 10^−4^	4.54	205290_s_at	BMP2	bone morphogenetic protein 2	8.44 × 10^−2^	2.25
228952_at	ENPP1	ectonucleotide pyrophosphatase/phosphodiesterase 1	5.77 × 10^−7^	4.95	211518_s_at	BMP4	bone morphogenetic protein 4	1.68 × 10^−2^	4.03
229088_at	ENPP1	ectonucleotide pyrophosphatase/phosphodiesterase 1	5.38 × 10^−7^	4.83	206176_at	BMP6	bone morphogenetic protein 6	2.52 × 10^−2^	2.33
205066_s_at	ENPP1	ectonucleotide pyrophosphatase/phosphodiesterase 1	3.10 × 10^−5^	4.76	239349_at	C1QTNF7	C1q and tumor necrosis factor related protein 7	2.49 × 10^−1^	1.53
205065_at	ENPP1	ectonucleotide pyrophosphatase/phosphodiesterase 1	3.78 × 10^−7^	3.82	202357_s_at	C2 CFB	complement component 2 complement factor B	3.16 × 10^−3^	3.85
205110_s_at	FGF13	fibroblast growth factor 13	3.58 × 10^−8^	4.73	217767_at	C3	complement component 3	2.71 × 10^−1^	2.42
214240_at	GAL	galanin prepropeptide	9.61 × 10^−2^	1.59	208451_s_at	C4A C4B	complement component 4A (Rodgers blood group) complement component 4B (Chido blood group)	1.98 × 10^−1^	1.83
205505_at	GCNT1	glucosaminyl (N-acetyl) transferase 1, core 2 (beta-1,6-N-acetylglucosaminyltransferase)	1.70 × 10^−5^	1.66	206407_s_at	CCL13	chemokine (C-C motif) ligand 13	8.28 × 10^−2^	3.52
240509_s_at	GREM2	gremlin 2, cysteine knot superfamily, homolog (Xenopus laevis)	2.66 × 10^−3^	3.23	216598_s_at	CCL2	chemokine (C-C motif) ligand 2	5.57 × 10^−4^	8.18
235504_at	GREM2	gremlin 2, cysteine knot superfamily, homolog (Xenopus laevis)	9.10 × 10^−3^	3.11	206508_at	CD70	CD70 molecule	1.14 × 10^−1^	2.44
220794_at	GREM2	gremlin 2, cysteine knot superfamily, homolog (Xenopus laevis)	2.06 × 10^−2^	2.76	213800_at	CFH	complement factor H	8.63 × 10^−5^	6.48
206326_at	GRP	gastrin-releasing peptide	3.05 × 10^−2^	1.59	215388_s_at	CFH CFHR1	complement factor H complement factor H-related 1	4.95 × 10^−6^	10.91
203821_at	HBEGF	heparin-binding EGF-like growth factor	1.31 × 10^−1^	1.64	206910_x_at	CFHR2	complement factor H-related 2	8.81 × 10^−3^	1.60
203819_s_at	IGF2BP3	insulin-like growth factor 2 mRNA binding protein 3	4.18 × 10^−2^	2.51	209395_at	CHI3L1	chitinase 3-like 1 (cartilage glycoprotein-39)	1.10 × 10^−2^	4.08
203820_s_at	IGF2BP3	insulin-like growth factor 2 mRNA binding protein 3	6.30 × 10^−2^	2.13	209396_s_at	CHI3L1	chitinase 3-like 1 (cartilage glycoprotein-39)	4.55 × 10^−2^	1.94
212143_s_at	IGFBP3	insulin-like growth factor binding protein 3	1.26 × 10^−2^	1.54	206315_at	CRLF1	cytokine receptor-like factor 1	8.40 × 10^−3^	3.23
227760_at	IGFBPL1	insulin-like growth factor binding protein-like 1	3.76 × 10^−3^	1.94	209774_x_at	CXCL2	chemokine (C-X-C motif) ligand 2	1.37 × 10^−1^	3.03
204773_at	IL11RA	interleukin 11 receptor, alpha	1.36 × 10^−3^	1.97	207850_at	CXCL3	chemokine (C-X-C motif) ligand 3	2.50 × 10^−1^	2.07
206172_at	IL13RA2	interleukin 13 receptor, alpha 2	8.39 × 10^−2^	3.56	219837_s_at	CYTL1	cytokine-like 1	1.99 × 10^−1^	2.02
227997_at	IL17RD	interleukin 17 receptor D	9.70 × 10^−2^	1.70	219501_at	ENOX1	ecto-NOX disulfide-thiol exchanger 1	1.82 × 10^−2^	2.35
222062_at	IL27RA	interleukin 27 receptor, alpha	4.50 × 10^−4^	3.73	226213_at	ERBB3	v-erb-b2 erythroblastic leukemia viral oncogene homolog 3 (avian)	1.84 × 10^−2^	2.49
205926_at	IL27RA	interleukin 27 receptor, alpha	5.58 × 10^−4^	1.67	205738_s_at	FABP3	fatty acid binding protein 3, muscle and heart (mammary-derived growth inhibitor)	2.56 × 10^−1^	1.70
226218_at	IL7R	interleukin 7 receptor	9.41 × 10^−2^	1.71	203980_at	FABP4	fatty acid binding protein 4, adipocyte	5.11 × 10^−2^	2.16
205798_at	IL7R	interleukin 7 receptor	1.78 × 10^−1^	1.59	205117_at	FGF1	fibroblast growth factor 1 (acidic)	8.61 × 10^−3^	2.95
231798_at	NOG	noggin	6.76 × 10^−5^	3.98	1552721_a_at	FGF1	fibroblast growth factor 1 (acidic)	1.47 × 10^−2^	2.71
206343_s_at	NRG1	neuregulin 1	6.34 × 10^−3^	2.68	208240_s_at	FGF1	fibroblast growth factor 1 (acidic)	9.05 × 10^−2^	1.73
206237_s_at	NRG1	neuregulin 1	7.90 × 10^−2^	2.15	231382_at	FGF18	Fibroblast growth factor 18	1.71 × 10^−1^	2.09
208230_s_at	NRG1	neuregulin 1	1.20 × 10^−2^	1.90	211029_x_at	FGF18	fibroblast growth factor 18	2.54 × 10^−1^	1.64
204766_s_at	NUDT1	nudix (nucleoside diphosphate linked moiety X)-type motif 1	2.22 × 10^−5^	1.59	221577_x_at	GDF15	growth differentiation factor 15	3.41 × 10^−2^	2.03
213131_at	OLFM1	olfactomedin 1	1.82 × 10^−1^	1.71	206614_at	GDF5	growth differentiation factor 5	4.96 × 10^−2^	2.01
213125_at	OLFML2B	olfactomedin-like 2B	2.17 × 10^−1^	1.93	201348_at	GPX3	glutathione peroxidase 3 (plasma)	2.15 × 10^−2^	3.24
218162_at	OLFML3	olfactomedin-like 3	3.28 × 10^−2^	1.72	214091_s_at	GPX3	glutathione peroxidase 3 (plasma)	7.64 × 10^−2^	1.99
222719_s_at	PDGFC	platelet derived growth factor C	2.01 × 10^−3^	1.61	209960_at	HGF	hepatocyte growth factor (hepapoietin A; scatter factor)	7.99 × 10^−2^	3.24
201578_at	PODXL	podocalyxin-like	1.11 × 10^−2^	5.15	210997_at	HGF	hepatocyte growth factor (hepapoietin A; scatter factor)	2.26 × 10^−2^	2.96
210195_s_at	PSG1	pregnancy specific beta-1-glycoprotein 1	2.14 × 10^−1^	1.82	210998_s_at	HGF	hepatocyte growth factor (hepapoietin A; scatter factor)	3.94 × 10^−2^	1.80
208134_x_at	PSG2	pregnancy specific beta-1-glycoprotein 2	1.56 × 10^−3^	3.17	210619_s_at	HYAL1	hyaluronoglucosaminidase 1	1.86 × 10^−3^	3.19
203399_x_at	PSG3	pregnancy specific beta-1-glycoprotein 3	8.06 × 10^−3^	3.33	209540_at	IGF1	insulin-like growth factor 1 (somatomedin C)	2.78 × 10^−1^	1.97
215821_x_at	PSG3	pregnancy specific beta-1-glycoprotein 3	2.08 × 10^−2^	2.47	209542_x_at	IGF1	insulin-like growth factor 1 (somatomedin C)	2.03 × 10^−1^	1.57
211741_x_at	PSG3	pregnancy specific beta-1-glycoprotein 3	1.76 × 10^−2^	2.22	202718_at	IGFBP2	insulin-like growth factor binding protein 2, 36 kDa	8.69 × 10^−2^	3.80
204830_x_at	PSG5	pregnancy specific beta-1-glycoprotein 5	8.14 × 10^−3^	3.53	201508_at	IGFBP4	insulin-like growth factor binding protein 4	1.12 × 10^−1^	1.68
209738_x_at	PSG6	pregnancy specific beta-1-glycoprotein 6	7.00 × 10^−3^	3.35	203426_s_at	IGFBP5	insulin-like growth factor binding protein 5	2.55 × 10^−2^	3.31
208106_x_at	PSG6	pregnancy specific beta-1-glycoprotein 6	4.99 × 10^−3^	3.26	211958_at	IGFBP5	insulin-like growth factor binding protein 5	2.16 × 10^−1^	2.76
209594_x_at	PSG9	pregnancy specific beta-1-glycoprotein 9	5.91 × 10^−3^	3.49	1555997_s_at	IGFBP5	insulin-like growth factor binding protein 5	1.64 × 10^−1^	2.46
207733_x_at	PSG9	pregnancy specific beta-1-glycoprotein 9	1.20 × 10^−2^	3.03	203425_s_at	IGFBP5	insulin-like growth factor binding protein 5	1.58 × 10^−1^	1.80
212187_x_at	PTGDS	prostaglandin D2 synthase 21 kDa (brain)	5.94 × 10^−3^	1.91	206295_at	IL18	interleukin 18 (interferon-gamma-inducing factor)	3.70 × 10^−2^	3.75
211748_x_at	PTGDS	prostaglandin D2 synthase 21 kDa (brain)	2.12 × 10^−3^	1.69	207526_s_at	IL1RL1	interleukin 1 receptor-like 1	1.32 × 10^−1^	2.07
206631_at	PTGER2	prostaglandin E receptor 2 (subtype EP2), 53 kDa	4.68 × 10^−2^	1.71	242809_at	IL1RL1	Interleukin 1 receptor-like 1	2.38 × 10^−1^	1.60
211737_x_at	PTN	pleiotrophin	7.25 × 10^−2^	3.28	221111_at	IL26	interleukin 26	4.68 × 10^−2^	3.44
209465_x_at	PTN	pleiotrophin	4.83 × 10^−2^	3.24	209821_at	IL33	interleukin 33	1.79 × 10^−1^	1.50
209466_x_at	PTN	pleiotrophin	1.03 × 10^−1^	2.42	205207_at	IL6	interleukin 6 (interferon, beta 2)	2.42 × 10^−4^	3.20
209897_s_at	SLIT2	slit homolog 2 (Drosophila)	1.55 × 10^−2^	1.98	204863_s_at	IL6ST	interleukin 6 signal transducer (gp130, oncostatin M receptor)	1.97 × 10^−2^	1.99
205016_at	TGFA	transforming growth factor, alpha	4.19 × 10^−2^	3.27	211000_s_at	IL6ST	interleukin 6 signal transducer (gp130, oncostatin M receptor)	1.21 × 10^−2^	1.88
203085_s_at	TGFB1	transforming growth factor, beta 1	7.50 × 10^−^3	1.60	204926_at	INHBA	inhibin, beta A	1.44 × 10^−2^	2.47
236561_at	TGFBR1	Transforming growth factor, beta receptor 1	4.05 × 10^−2^	1.71	210511_s_at	INHBA	inhibin, beta A	4.64 × 10^−3^	2.42
203887_s_at	THBD	thrombomodulin	2.94 × 10^−1^	2.27	205266_at	LIF	leukemia inhibitory factor (cholinergic differentiation factor)	7.91 × 10^−2^	2.44
239336_at	THBS1	Thrombospondin 1	1.84 × 10^−1^	1.58	219181_at	LIPG	lipase, endothelial	3.51 × 10^−2^	2.89
227420_at	TNFAIP8L1	tumor necrosis factor, alpha-induced protein 8-like 1	3.38 × 10^−3^	1.71	205381_at	LRRC17	leucine rich repeat containing 17	2.67 × 10^−4^	18.57
219478_at	WFDC1	WAP four-disulfide core domain 1	3.17 × 10^−2^	5.38	216320_x_at	MST1	macrophage stimulating 1 (hepatocyte growth factor-like)	1.05 × 10^−1^	1.53
221029_s_at	WNT5B	wingless-type MMTV integration site family, member 5B	5.20 × 10^−4^	2.39	231361_at	NLGN1	Neuroligin 1	4.86 × 10^−2^	3.33
223537_s_at	WNT5B	wingless-type MMTV integration site family, member 5B	2.99 × 10^−2^	1.65	205893_at	NLGN1	neuroligin 1	7.72 × 10^−2^	3.23
					204501_at	NOV	nephroblastoma overexpressed gene	2.01 × 10^−2^	2.95
					214321_at	NOV	nephroblastoma overexpressed gene	1.69 × 10^−2^	2.60
					217525_at	OLFML1	olfactomedin-like 1	5.70 × 10^−2^	3.66
					213075_at	OLFML2A	olfactomedin-like 2A	1.64 × 10^−2^	2.12
					205729_at	OSMR	oncostatin M receptor	6.53 × 10^−2^	1.71
					224942_at	PAPPA	pregnancy-associated plasma protein A, pappalysin 1	2.20 × 10^−2^	1.88
					1559400_s_at	PAPPA	pregnancy-associated plasma protein A, pappalysin 1	3.28 × 10^−2^	1.84
					201981_at	PAPPA	pregnancy-associated plasma protein A, pappalysin 1	3.78 × 10^−2^	1.74
					224940_s_at	PAPPA	pregnancy-associated plasma protein A, pappalysin 1	1.72 × 10^−2^	1.73
					224941_at	PAPPA	pregnancy-associated plasma protein A, pappalysin 1	1.36 × 10^−2^	1.69
					228128_x_at	PAPPA	pregnancy-associated plasma protein A, pappalysin 1	2.13 × 10^−2^	1.64
					205560_at	PCSK5	proprotein convertase subtilisin/kexin type 5	2.21 × 10^−3^	2.70
					213652_at	PCSK5	Proprotein convertase subtilisin/kexin type 5	5.18 × 10^−4^	2.66
					205559_s_at	PCSK5	proprotein convertase subtilisin/kexin type 5	1.23 × 10^−3^	2.52
					227759_at	PCSK9	proprotein convertase subtilisin/kexin type 9	2.34 × 10^−2^	1.97
					216867_s_at	PDGFA	platelet-derived growth factor alpha polypeptide	2.46 × 10^−2^	1.97
					222860_s_at	PDGFD	platelet derived growth factor D	1.50 × 10^−1^	1.57
					1555778_a_at	POSTN	periostin, osteoblast specific factor	4.41 × 10^−3^	4.30
					214981_at	POSTN	periostin, osteoblast specific factor	2.20 × 10^−3^	2.96
					210809_s_at	POSTN	periostin, osteoblast specific factor	8.30 × 10^−3^	2.47
					207808_s_at	PROS1	protein S (alpha)	3.88 × 10^−3^	1.51
					213421_x_at	PRSS3	protease, serine, 3	2.41 × 10^−2^	2.68
					210367_s_at	PTGES	prostaglandin E synthase	3.17 × 10^−4^	4.21
					207388_s_at	PTGES	prostaglandin E synthase	4.23 × 10^−2^	2.51
					224950_at	PTGFRN	prostaglandin F2 receptor negative regulator	4.86 × 10^−2^	1.78
					211892_s_at	PTGIS	prostaglandin I2 (prostacyclin) synthase	4.91 × 10^−2^	2.06
					210702_s_at	PTGIS	prostaglandin I2 (prostacyclin) synthase	1.13 × 10^−1^	1.70
					208131_s_at	PTGIS	prostaglandin I2 (prostacyclin) synthase	2.43 × 10^−2^	1.67
					211756_at	PTHLH	parathyroid hormone-like hormone	6.78 × 10^−2^	3.45
					206300_s_at	PTHLH	parathyroid hormone-like hormone	7.08 × 10^−2^	2.86
					210355_at	PTHLH	parathyroid hormone-like hormone	1.70 × 10^−1^	2.21
					206157_at	PTX3	pentraxin-related gene, rapidly induced by IL-1 beta	1.47 × 10^−4^	2.21
					201482_at	QSOX1	quiescin Q6 sulfhydryl oxidase 1	2.19 × 10^−3^	1.68
					223824_at	RNLS	renalase, FAD-dependent amine oxidase	5.18 × 10^−4^	1.74
					204035_at	SCG2	secretogranin II (chromogranin C)	2.66 × 10^−2^	2.56
					205475_at	SCRG1	scrapie responsive protein 1	6.88 × 10^−7^	7.73
					213716_s_at	SECTM1	secreted and transmembrane 1	3.32 × 10^−2^	3.29
					203071_at	SEMA3B	sema domain, immunoglobulin domain (Ig), short basic domain, secreted, (semaphorin) 3B	6.35 × 10^−2^	2.54
					203788_s_at	SEMA3C	sema domain, immunoglobulin domain (Ig), short basic domain, secreted, (semaphorin) 3C	6.34 × 10^−4^	2.51
					203789_s_at	SEMA3C	sema domain, immunoglobulin domain (Ig), short basic domain, secreted, (semaphorin) 3C	3.13 × 10^−3^	1.64
					33323_r_at	SFN	stratifin	1.80 × 10^−1^	2.42
					223122_s_at	SFRP2	secreted frizzled-related prot 2	2.86 × 10^−1^	2.33
					204051_s_at	SFRP4	secreted frizzled-related prot 4	4.79 × 10^−2^	4.23
					204052_s_at	SFRP4	secreted frizzled-related prot 4	1.56 × 10^−1^	2.58
					210665_at	TFPI	tissue factor pathway inhibitor (lipoprotein-associated coagulation inhibitor)	4.02 × 10^−2^	3.69
					210664_s_at	TFPI	tissue factor pathway inhibitor (lipoprotein-associated coagulation inhibitor)	2.35 × 10^−2^	3.50
					209676_at	TFPI	tissue factor pathway inhibitor (lipoprotein-associated coagulation inhibitor)	2.96 × 10^−2^	3.35
					213258_at	TFPI	tissue factor pathway inhibitor (lipoprotein-associated coagulation inhibitor)	2.61 × 10^−2^	3.24
					228121_at	TGFB2	transforming growth factor, beta 2	1.08 × 10^−6^	5.13
					209909_s_at	TGFB2	transforming growth factor, beta 2	5.47 × 10^−5^	3.64
					204731_at	TGFBR3	transforming growth factor, beta receptor III	2.06 × 10^−1^	1.70
					203083_at	THBS2	thrombospondin 2	4.84 × 10^−6^	2.83
					202644_s_at	TNFAIP3	tumor necrosis factor, alpha-induced protein 3	1.61 × 10^−3^	3.88
					202643_s_at	TNFAIP3	tumor necrosis factor, alpha-induced protein 3	4.35 × 10^−3^	3.52
					206025_s_at	TNFAIP6	tumor necrosis factor, alpha-induced protein 6	1.57 × 10^−2^	2.29
					206026_s_at	TNFAIP6	tumor necrosis factor, alpha-induced protein 6	3.57 × 10^−2^	2.15
					210260_s_at	TNFAIP8	tumor necrosis factor, alpha-induced protein 8	5.99 × 10^−4^	2.01
					208296_x_at	TNFAIP8	tumor necrosis factor, alpha-induced protein 8	1.11 × 10^−3^	1.94
					235737_at	TSLP	thymic stromal lymphopoietin	1.72 × 10^−1^	2.02
					210513_s_at	VEGFA	vascular endothelial growth factor A	9.18 × 10^−2^	1.57
					205648_at	WNT2	wingless-type MMTV integration site family member 2	2.83 × 10^−1^	2.08
					202643_s_at	TNFAIP3	tumor necrosis factor, alpha-induced protein 3	4.35 × 10^−3^	3.52
					206025_s_at	TNFAIP6	tumor necrosis factor, alpha-induced protein 6	1.57 × 10^−2^	2.29
					206026_s_at	TNFAIP6	tumor necrosis factor, alpha-induced protein 6	3.57 × 10^−2^	2.15
					210260_s_at	TNFAIP8	tumor necrosis factor, alpha-induced protein 8	5.99 × 10^−4^	2.01
					208296_x_at	TNFAIP8	tumor necrosis factor, alpha-induced protein 8	1.11 × 10^−3^	1.94
					235737_at	TSLP	thymic stromal lymphopoietin	1.72 × 10^−1^	2.02
					210513_s_at	VEGFA	vascular endothelial growth factor A	9.18 × 10^−2^	1.57
					205648_at	WNT2	wingless-type MMTV integration site family member 2	2.83 × 10^−1^	2.08

**Table 4:** Transcripts related to the tissue skeleton, in which differential expressions distinguish the “fibroblast” and mesenchymal stem cell or “MSC” groups. This transcript list was extracted from microarray data using fold-change >2 and *p* < 0.05 as inclusion parameters. The transcript signature with predominant expression in the “fibroblast” group concerned 424 probe sets corresponding to transcripts directly involved in the tissue skeleton structure, comprising 145 transcripts related to ECM, 63 focal adhesion point transcripts, 68 cytoskeleton transcripts, 4 LINC complex transcripts, and 12 nucleoskeleton transcripts. The transcript signature with predominant expression in the “MSC” group concerned 241 probe sets corresponding to transcripts directly involved in the tissue skeleton structure, comprising 53 transcripts related to ECM, 63 focal adhesion point transcripts, 52 cytoskeleton transcripts, 2 LINC complex transcripts, and 7 nucleoskeleton transcripts. In addition, transcripts encoding soluble factors were found in both signatures, respectively 132 and 79 for the “fibroblast” and “MSC” groups.

UP in Dermal Fibroblasts					UP in MSCs				
**Extracellular Matrix Genes**									
**Probe Set ID**	**Gene Symbol**	**Gene Title**	**adj-pval**	**FC**	**Probe Set ID**	**Gene Symbol**	**Gene Title**	**adj-pval**	**FC**
205679_x_at	ACAN	aggrecan	8.39 × 10^−22^	25.41	209765_at	ADAM19	ADAM metallopeptidase domain 19 (meltrin beta)	7.41 × 10^−28^	8.60
207692_s_at	ACAN	aggrecan	7.55 × 10^−21^	23.23	226997_at	ADAMTS12	ADAM metallopeptidase with thrombospondin type 1 motif, 12	9.13 × 10^−13^	4.00
217161_x_at	ACAN	aggrecan	1.14 × 10^−20^	20.79	214913_at	ADAMTS3	ADAM metallopeptidase with thrombospondin type 1 motif, 3	1.26 × 10^−10^	4.41
232570_s_at	ADAM33	ADAM metallopeptidase domain 33	2.07 × 10^−9^	4.20	1570351_at	ADAMTS6	ADAM metallopeptidase with thrombospondin type 1 motif, 6	3.98 × 10^−15^	2.31
233868_x_at	ADAM33	ADAM metallopeptidase domain 33	3.16 × 10^−8^	2.98	222043_at	CLU	clusterin	5.59 × 10^−4^	2.05
214454_at	ADAMTS2	ADAM metallopeptidase with thrombospondin type 1 motif, 2	4.55 × 10^−14^	2.74	225288_at	COL27A1	collagen, type XXVII, alpha 1	3.75 × 10^−9^	2.35
229357_at	ADAMTS5	ADAM metallopeptidase with thrombospondin type 1, motif, 5	1.26 × 10^−16^	22.33	213110_s_at	COL4A5	collagen, type IV, alpha 5	2.61 × 10^−3^	3.17
219935_at	ADAMTS5	ADAM metallopeptidase with thrombospondin type 1 motif, 5	6.18 × 10^−16^	21.71	204136_at	COL7A1	collagen, type VII, alpha 1	5.86 × 10^−5^	2.46
235368_at	ADAMTS5	ADAM metallopeptidase with thrombospondin type 1 motif, 5	1.86 × 10^−15^	11.70	223475_at	CRISPLD1	cysteine-rich secretory protein LCCL domain containing 1	2.40 × 10^−5^	2.02
219087_at	ASPN	asporin	3.78 × 10^−15^	18.79	201487_at	CTSC	cathepsin C	2.84 × 10^−13^	3.90
224396_s_at	ASPN	asporin	6.26 × 10^−6^	3.63	225646_at	CTSC	cathepsin C	1.77 × 10^−7^	2.77
201262_s_at	BGN	biglycan	4.64 × 10^−10^	3.10	225647_s_at	CTSC	cathepsin C	4.24 × 10^−7^	2.64
213905_x_at	BGN	biglycan	5.80 × 10^−7^	2.15	231234_at	CTSC	cathepsin C	7.29 × 10^−8^	2.40
201261_x_at	BGN	biglycan	1.53 × 10^−6^	2.00	229115_at	DYNC1H1	dynein, cytoplasmic 1, heavy chain 1	8.64 × 10^−5^	2.02
241986_at	BMPER	BMP binding endothelial regulator	1.17 × 10^−9^	2.75	207379_at	EDIL3	EGF-like repeats and discoidin I-like domains 3	2.94 × 10^−4^	2.09
227526_at	CDON	Cdon homolog (mouse)	1.16 × 10^−6^	2.34	226911_at	EGFLAM	EGF-like, fibronectin type III and laminin G domains	5.99 × 10^−5^	3.25
209732_at	CLEC2B	C-type lectin domain family 2, member B	9.58 × 10^−18^	66.68	203184_at	FBN2	fibrillin 2	5.58 × 10^−5^	6.34
1556209_at	CLEC2B	C-type lectin domain family 2, member B	4.34 × 10^−6^	4.46	236028_at	IBSP	integrin-binding sialoprotein	1.83 × 10^−3^	2.85
205200_at	CLEC3B	C-type lectin domain family 3, member B	1.76 × 10^−12^	17.53	223689_at	IGF2BP1	insulin-like growth factor 2 mRNA binding protein 1	1.37 × 10^−17^	5.58
217428_s_at	COL10A1	collagen, type X, alpha 1	3.42 × 10^−4^	3.59	203819_s_at	IGF2BP3	insulin-like growth factor 2 mRNA binding protein 3	1.72 × 10^−28^	39.96
205941_s_at	COL10A1	collagen, type X, alpha 1	5.32 × 10^−4^	3.30	203820_s_at	IGF2BP3	insulin-like growth factor 2 mRNA binding protein 3	1.52 × 10^−29^	30.51
231879_at	COL12A1	collagen, type XII, alpha 1	7.44 × 10^−15^	5.04	216493_s_at	IGF2BP3	insulin-like growth factor 2 mRNA binding protein 3	2.04 × 10^−22^	6.25
234951_s_at	COL12A1	collagen, type XII, alpha 1	3.01 × 10^−8^	3.71	205206_at	KAL1	Kallmann syndrome 1 sequence	8.59 × 10^−6^	5.25
225664_at	COL12A1	collagen, type XII, alpha 1	5.13 × 10^−11^	2.60	202728_s_at	LTBP1	latent transforming growth factor beta binding protein 1	3.50 × 10^−12^	4.74
231766_s_at	COL12A1	collagen, type XII, alpha 1	1.68 × 10^−6^	2.34	202729_s_at	LTBP1	latent transforming growth factor beta binding protein 1	2.67 × 10^−12^	3.43
203477_at	COL15A1	collagen, type XV, alpha 1	2.08 × 10^−11^	18.96	223614_at	MMP16	matrix metallopeptidase 16 (membrane-inserted)	7.85 × 10^−11^	3.87
211966_at	COL4A2	collagen, type IV, alpha 2	2.64 × 10^−4^	2.52	207012_at	MMP16	matrix metallopeptidase 16 (membrane-inserted)	4.43 × 10^−13^	3.63
226277_at	COL4A3BP	collagen, type IV, alpha 3 (Goodpasture antigen) binding protein	6.82 × 10^−34^	2.01	229346_at	NES	nestin	6.51 × 10^−18^	6.19
229779_at	COL4A4	collagen, type IV, alpha 4	2.16 × 10^−6^	3.00	218678_at	NES	nestin	3.67 × 10^−9^	3.84
221900_at	COL8A2	collagen, type VIII, alpha 2	1.22 × 10^−7^	4.76	201860_s_at	PLAT	plasminogen activator, tissue	3.31 × 10^−16^	6.86
52651_at	COL8A2	collagen, type VIII, alpha 2	3.02 × 10^−8^	3.51	205479_s_at	PLAU	plasminogen activator, urokinase	4.34 × 10^−28^	22.23
205713_s_at	COMP	cartilage oligomeric matrix protein	1.03 × 10^−26^	115.83	211668_s_at	PLAU	plasminogen activator, urokinase	2.42 × 10^−20^	12.30
226824_at	CPXM2	carboxypeptidase X (M14 family), member 2	1.74 × 10^−8^	5.71	211924_s_at	PLAUR	plasminogen activator, urokinase receptor	7.22 × 10^−17^	2.73
208978_at	CRIP2	cysteine-rich protein 2	6.66 × 10^−14^	6.14	210845_s_at	PLAUR	plasminogen activator, urokinase receptor	2.18 × 10^−22^	2.69
221541_at	CRISPLD2	cysteine-rich secretory protein LCCL domain containing 2	1.80 × 10^−5^	2.74	206007_at	PRG4	proteoglycan 4	1.12 × 10^−6^	2.17
204971_at	CSTA	cystatin A (stefin A)	9.43 × 10^−5^	3.43	221872_at	RARRES1	retinoic acid receptor responder (tazarotene induced) 1	5.43 × 10^−19^	6.46
209101_at	CTGF	connective tissue growth factor	5.63 × 10^−10^	2.09	206392_s_at	RARRES1	retinoic acid receptor responder (tazarotene induced) 1	1.34 × 10^−16^	4.46
200661_at	CTSA	cathepsin A	5.53 × 10^−17^	2.18	222784_at	SMOC1	SPARC related modular calcium binding 1	4.56 × 10^−4^	2.10
200766_at	CTSD	cathepsin D	5.83 × 10^−18^	2.96	201858_s_at	SRGN	serglycin	1.63 × 10^−6^	6.82
203657_s_at	CTSF	cathepsin F	1.31 × 10^−37^	7.43	201859_at	SRGN	serglycin	3.48 × 10^−7^	4.90
202295_s_at	CTSH	cathepsin H	8.10 × 10^−5^	2.03	209277_at	TFPI2	tissue factor pathway inhibitor 2	3.21 × 10^−13^	10.83
203758_at	CTSO	cathepsin O	5.40 × 10^−11^	2.37	209278_s_at	TFPI2	tissue factor pathway inhibitor 2	4.82 × 10^−12^	6.13
210042_s_at	CTSZ	cathepsin Z	4.41 × 10^−13^	2.63	209909_s_at	TGFB2	transforming growth factor, beta 2	1.36 × 10^−9^	7.65
209335_at	DCN	decorin	2.15 × 10^−17^	5.05	228121_at	TGFB2	transforming growth factor, beta 2	2.09 × 10^−7^	5.04
211896_s_at	DCN	decorin	2.64 × 10^−17^	4.08	220407_s_at	TGFB2	transforming growth factor, beta 2	2.05 × 10^−9^	3.60
211813_x_at	DCN	decorin	1.62 × 10^−16^	3.47	201042_at	TGM2	transglutaminase 2	7.33 × 10^−12^	8.42
201893_x_at	DCN	decorin	1.32 × 10^−16^	3.17	211573_x_at	TGM2	transglutaminase 2	6.53 × 10^−25^	4.14
213068_at	DPT	dermatopontin	2.61 × 10^−22^	47.71	211003_x_at	TGM2	transglutaminase 2	6.51 × 10^−23^	3.00
207977_s_at	DPT	dermatopontin	1.64 × 10^−19^	20.11	222835_at	THSD4	thrombospondin, type I, domain containing 4	2.65 × 10^−9^	4.10
213071_at	DPT	dermatopontin	2.41 × 10^−18^	14.09	226506_at	THSD4	thrombospondin, type I, domain containing 4	3.76 × 10^−7^	3.35
209365_s_at	ECM1	extracellular matrix protein 1	1.55 × 10^−26^	3.53	202643_s_at	TNFAIP3	tumor necrosis factor, alpha-induced protein 3	1.91 × 10^−9^	4.48
206101_at	ECM2	extracellular matrix protein 2, female organ and adipocyte specific	6.39 × 10^−13^	10.63	202644_s_at	TNFAIP3	tumor necrosis factor, alpha-induced protein 3	2.35 × 10^−8^	3.98
201843_s_at	EFEMP1	EGF-containing fibulin-like extracellular matrix protein 1	4.72 × 10^−3^	2.04	206025_s_at	TNFAIP6	tumor necrosis factor, alpha-induced protein 6	4.06 × 10^−5^	2.04
209356_x_at	EFEMP2	EGF-containing fibulin-like extracellular matrix protein 2	2.39 × 10^−21^	3.14					
212670_at	ELN	elastin	0.00 × 10^+00^	34.17					
222885_at	EMCN	endomucin	6.61 × 10^−8^	4.14					
227874_at	EMCN	endomucin	6.01 × 10^−6^	2.25					
204363_at	F3	coagulation factor III (thromboplastin, tissue factor)	2.04 × 10^−6^	5.30					
202995_s_at	FBLN1	fibulin 1	4.84 × 10^−8^	2.48					
203886_s_at	FBLN2	fibulin 2	7.69 × 10^−21^	18.44					
203088_at	FBLN5	fibulin 5	7.88 × 10^−28^	10.14					
203638_s_at	FGFR2	fibroblast growth factor receptor 2	1.16 × 10^−11^	6.98					
227265_at	FGL2	fibrinogen-like 2	1.58 × 10^−5^	6.33					
204834_at	FGL2	fibrinogen-like 2	2.20 × 10^−7^	5.59					
202709_at	FMOD	fibromodulin	4.30 × 10^−22^	8.88					
226930_at	FNDC1	fibronectin type III domain containing 1	2.46 × 10^−19^	47.43					
226145_s_at	FRAS1	Fraser syndrome 1	0.00 × 10^+00^	76.26					
202755_s_at	GPC1	glypican 1	3.75 × 10^−15^	2.10					
204984_at	GPC4	glypican 4	5.19 × 10^−15^	9.94					
204983_s_at	GPC4	glypican 4	8.43 × 10^−12^	5.40					
230204_at	HAPLN1	hyaluronan and proteoglycan link protein 1	2.18 × 10^−7^	7.98					
205523_at	HAPLN1	hyaluronan and proteoglycan link protein 1	5.24 × 10^−7^	7.86					
205524_s_at	HAPLN1	hyaluronan and proteoglycan link protein 1	5.34 × 10^−8^	7.86					
230895_at	HAPLN1	hyaluronan and proteoglycan link protein 1	6.93 × 10^−7^	7.40					
227262_at	HAPLN3	hyaluronan and proteoglycan link protein 3	5.18 × 10^−16^	4.01					
235944_at	HMCN1	hemicentin 1	5.33 × 10^−8^	4.24					
201185_at	HTRA1	HtrA serine peptidase 1	3.62 × 10^−10^	3.08					
209541_at	IGF1	insulin-like growth factor 1 (somatomedin C)	4.41 × 10^−6^	2.51					
202718_at	IGFBP2	insulin-like growth factor binding protein 2, 36 kDa	1.20 × 10^−2^	2.37					
212143_s_at	IGFBP3	insulin-like growth factor binding protein 3	1.20 × 10^−23^	28.32					
210095_s_at	IGFBP3	insulin-like growth factor binding protein 3	5.39 × 10^−19^	9.31					
211959_at	IGFBP5	insulin-like growth factor binding protein 5	2.35 × 10^−11^	5.19					
203424_s_at	IGFBP5	insulin-like growth factor binding protein 5	7.05 × 10^−7^	4.65					
211958_at	IGFBP5	insulin-like growth factor binding protein 5	1.68 × 10^−7^	3.69					
1555997_s_at	IGFBP5	insulin-like growth factor binding protein 5	9.64 × 10^−6^	2.41					
203426_s_at	IGFBP5	insulin-like growth factor binding protein 5	2.17 × 10^−4^	2.08					
203851_at	IGFBP6	insulin-like growth factor binding protein 6	2.87 × 10^−7^	2.03					
227760_at	IGFBPL1	insulin-like growth factor binding protein-like 1	6.32 × 10^−20^	2.75					
218574_s_at	LMCD1	LIM and cysteine-rich domains 1	1.08 × 10^−20^	5.35					
242767_at	LMCD1	LIM and cysteine-rich domains 1	2.61 × 10^−5^	2.23					
201744_s_at	LUM	lumican	3.33 × 10^−9^	2.53					
212713_at	MFAP4	microfibrillar-associated prot 4	5.47 × 10^−25^	11.79					
209758_s_at	MFAP5	microfibrillar associated prot 5	8.86 × 10^−9^	9.84					
213765_at	MFAP5	microfibrillar associated prot 5	1.07 × 10^−7^	9.67					
213764_s_at	MFAP5	microfibrillar associated prot 5	8.21 × 10^−8^	8.97					
210605_s_at	MFGE8	milk fat globule-EGF factor 8 protein	2.85 × 10^−8^	3.58					
202291_s_at	MGP	matrix Gla protein	5.61 × 10^−5^	5.61					
204475_at	MMP1	matrix metallopeptidase 1 (interstitial collagenase)	3.34 × 10^−4^	3.31					
204580_at	MMP12	matrix metallopeptidase 12 (macrophage elastase)	8.73 × 10^−4^	2.60					
205828_at	MMP3	matrix metallopeptidase 3 (stromelysin 1, progelatinase)	5.32 × 10^−8^	16.81					
213693_s_at	MUC1	mucin 1, cell surface associated	2.96 × 10^−18^	3.90					
207847_s_at	MUC1	mucin 1, cell surface associated	3.37 × 10^−11^	3.78					
209596_at	MXRA5	matrix-remodelling associated 5	6.87 × 10^−26^	30.61					
235836_at	MXRA7	matrix-remodelling associated 7	7.77 × 10^−10^	2.16					
213422_s_at	MXRA8	matrix-remodelling associated 8	9.88 × 10^−30^	2.13					
214321_at	NOV	nephroblastoma overexpressed gene	6.98 × 10^−20^	10.34					
204501_at	NOV	nephroblastoma overexpressed gene	4.71 × 10^−9^	2.90					
1564494_s_at	P4HB	prolyl 4-hydroxylase, beta polypeptide	7.83 × 10^−23^	3.48					
219295_s_at	PCOLCE2	procollagen C-endopeptidase enhancer 2	3.15 × 10^−6^	7.20					
226522_at	PODN	podocan	3.74 × 10^−12^	3.16					
1555778_a_at	POSTN	periostin, osteoblast specific factor	1.43 × 10^−3^	2.70					
210809_s_at	POSTN	periostin, osteoblast specific factor	3.60 × 10^−4^	2.31					
228224_at	PRELP	proline/arginine-rich end leucine-rich repeat protein	1.49 × 10^−13^	6.34					
204223_at	PRELP	proline/arginine-rich end leucine-rich repeat protein	3.05 × 10^−10^	4.16					
209496_at	RARRES2	retinoic acid receptor responder (tazarotene induced) 2	5.58 × 10^−9^	4.86					
205923_at	RELN	reelin	8.98 × 10^−4^	2.09					
228186_s_at	RSPO3	R-spondin 3 homolog (Xenopus laevis)	5.88 × 10^−14^	5.81					
202037_s_at	SFRP1	secreted frizzled-related prot 1	1.60 × 10^−4^	2.53					
202035_s_at	SFRP1	secreted frizzled-related prot 1	2.84 × 10^−3^	2.35					
202036_s_at	SFRP1	secreted frizzled-related prot 1	3.39 × 10^−3^	2.18					
223122_s_at	SFRP2	secreted frizzled-related prot 2	1.65 × 10^−29^	54.89					
223121_s_at	SFRP2	secreted frizzled-related prot 2	5.66 × 10^−18^	13.22					
203813_s_at	SLIT3	slit homolog 3 (Drosophila)	1.22 × 10^−21^	10.53					
223869_at	SOST	sclerosteosis	1.85 × 10^−7^	4.21					
213247_at	SVEP1	sushi, von Willebrand factor type A, EGF and pentraxin domain containing 1	5.98 × 10^−13^	5.61					
219552_at	SVEP1	sushi, von Willebrand factor type A, EGF and pentraxin domain containing 1	8.40 × 10^−11^	2.87					
205016_at	TGFA	transforming growth factor, alpha	9.81 × 10^−19^	9.55					
203085_s_at	TGFB1	transforming growth factor beta 1	9.57 × 10^−14^	2.17					
239336_at	THBS1	Thrombospondin 1	7.00 × 10^−26^	7.23					
201107_s_at	THBS1	thrombospondin 1	7.00 × 10^−26^	6.92					
201108_s_at	THBS1	thrombospondin 1	5.67 × 10^−28^	4.10					
235086_at	THBS1	Thrombospondin 1	1.09 × 10^−16^	3.35					
215775_at	THBS1	Thrombospondin 1	5.62 × 10^−18^	2.96					
201109_s_at	THBS1	thrombospondin 1	7.70 × 10^−23^	2.47					
201150_s_at	TIMP3	TIMP metallopeptidase inhibitor 3	2.68 × 10^−9^	2.06					
201149_s_at	TIMP3	TIMP metallopeptidase inhibitor 3	2.03 × 10^−5^	2.02					
201645_at	TNC	tenascin C	6.48 × 10^−7^	2.67					
216005_at	TNC	Tenascin C	1.97 × 10^−3^	2.27					
213451_x_at	TNXA / TNXB	tenascin XA pseudogene tenascin XB	7.61 × 10^−9^	7.23					
206093_x_at	TNXA / TNXB	tenascin XA pseudogene tenascin XB	3.25 × 10^−8^	6.27					
216333_x_at	TNXA / TNXB	tenascin XA pseudogene tenascin XB	1.07 × 10^−7^	6.23					
208609_s_at	TNXB	tenascin XB	2.48 × 10^−5^	3.26					
235616_at	TSHZ2	teashirt zinc finger homeobox 2	8.68 × 10^−8^	2.28					
227899_at	VIT	vitrin	6.33 × 10^−10^	3.99					
210102_at	VWA5A	von Willebrand factor A domain containing 5A	9.24 × 10^−6^	2.64					
**Focal Adhesion Points**									
**Probe Set ID**	**Gene Symbol**	**Gene Title**	**adj-pval**	**FC**	**Probe Set ID**	**Gene Symbol**	**Gene Title**	**adj-pval**	**FC**
200965_s_at	ABLIM1	actin binding LIM protein 1	5.19 × 10^−5^	2.74	206385_s_at	ANK3	ankyrin 3, node of Ranvier (ankyrin G)	4.82 × 10^−8^	3.98
205268_s_at	ADD2	adducin 2 (beta)	6.03 × 10^−7^	3.71	218950_at	ARAP3	ArfGAP with RhoGAP domain, ankyrin repeat and PH domain 3	3.65 × 10^−15^	2.95
202022_at	ALDOC	aldolase C, fructose-bisphosphate	6.50 × 10^−21^	6.09	227911_at	ARHGAP28	Rho GTPase activating protein 28	2.26 × 10^−11^	3.59
202920_at	ANK2	ankyrin 2, neuronal	7.80 × 10^−8^	3.58	206167_s_at	ARHGAP6	Rho GTPase activating protein 6	1.68 × 10^−8^	2.90
213606_s_at	ARHGDIA	Rho GDP dissociation inhibitor (GDI) alpha	2.12 × 10^−15^	2.88	1555812_a_at	ARHGDIB	Rho GDP dissociation inhibitor (GDI) beta	4.29 × 10^−5^	2.17
201167_x_at	ARHGDIA	Rho GDP dissociation inhibitor (GDI) alpha	2.10 × 10^−14^	2.36	218501_at	ARHGEF3	Rho guanine nucleotide exchange factor (GEF) 3	1.16 × 10^−15^	3.82
222696_at	AXIN2	axin 2	3.23 × 10^−6^	2.37	227372_s_at	BAIAP2L1	BAI1-associated protein 2-like 1	6.04 × 10^−14^	2.08
227850_x_at	CDC42EP5	CDC42 effector protein (Rho GTPase binding) 5	3.62 × 10^−8^	2.20	213373_s_at	CASP8	caspase 8, apoptosis-related cysteine peptidase	7.10 × 10^−17^	2.15
228739_at	CYS1	cystin 1	4.46 × 10^−26^	5.02	234936_s_at	CC2D2A	coiled-coil and C2 domain containing 2A	3.92 × 10^−26^	3.10
220559_at	EN1	engrailed homeobox 1	2.00 × 10^−19^	15.83	203881_s_at	DMD	dystrophin	8.99 × 10^−5^	2.84
206710_s_at	EPB41L3	erythrocyte membrane protein band 4.1-like 3	4.86 × 10^−4^	3.86	242283_at	DNAH14	dynein, axonemal, heavy chain 14	4.14 × 10^−18^	2.72
212681_at	EPB41L3	erythrocyte membrane protein band 4.1-like 3	4.79 × 10^−4^	3.39	205186_at	DNALI1	dynein, axonemal, light intermediate chain 1	3.02 × 10^−12^	2.71
211776_s_at	EPB41L3	erythrocyte membrane protein band 4.1-like 3	5.45 × 10^−4^	3.26	227081_at	DNALI1	dynein, axonemal, light intermediate chain 1	1.38 × 10^−6^	2.00
226129_at	FAM83H	family with sequence similarity 83, member H	1.37 × 10^−9^	2.03	212838_at	DNMBP	dynamin binding protein	2.88 × 10^−16^	2.05
212288_at	FNBP1	formin binding protein 1	9.29 × 10^−19^	2.31	228674_s_at	EML4	echinoderm microtubule associated protein like 4	1.66 × 10^−19^	2.39
230389_at	FNBP1	formin binding protein 1	2.64 × 10^−11^	2.19	220386_s_at	EML4	echinoderm microtubule associated protein like 4	1.34 × 10^−16^	2.32
230645_at	FRMD3	FERM domain containing 3	8.23 × 10^−12^	3.10	223068_at	EML4	echinoderm microtubule associated protein like 4	3.04 × 10^−22^	2.18
229893_at	FRMD3	FERM domain containing 3	1.74 × 10^−10^	2.18	201340_s_at	ENC1	ectodermal-neural cortex (with BTB-like domain)	4.58 × 10^−7^	2.76
226364_at	HIP1	Huntingtin interacting prot 1	2.94 × 10^−7^	2.09	1555137_a_at	FGD6	FYVE, RhoGEF and PH domain containing 6	2.15 × 10^−8^	2.23
209558_s_at	HIP1R	huntingtin interacting prot 1 related	3.28 × 10^−15^	2.09	219901_at	FGD6	FYVE, RhoGEF and PH domain containing 6	1.02 × 10^−8^	2.22
226352_at	JMY	junction mediating and regulatory protein, p53 cofactor	1.44 × 10^−15^	5.56	225167_at	FRMD4A	FERM domain containing 4A	2.68 × 10^−19^	4.09
241985_at	JMY	junction mediating and regulatory protein, p53 cofactor	1.04 × 10^−16^	2.12	208476_s_at	FRMD4A	FERM domain containing 4A	3.98 × 10^−15^	3.51
226534_at	KITLG	KIT ligand	1.06 × 10^−5^	2.21	225163_at	FRMD4A	FERM domain containing 4A	3.05 × 10^−17^	3.31
213371_at	LDB3	LIM domain binding 3	1.66 × 10^−2^	2.05	225168_at	FRMD4A	FERM domain containing 4A	9.69 × 10^−17^	2.99
227219_x_at	MAP1LC3A	microtubule-associated protein 1 light chain 3 alpha	1.93 × 10^−18^	2.79	1560031_at	FRMD4A	FERM domain containing 4A	2.08 × 10^−13^	2.76
224378_x_at	MAP1LC3A	microtubule-associated protein 1 light chain 3 alpha	2.64 × 10^−14^	2.72	230831_at	FRMD5	FERM domain containing 5	1.24 × 10^−19^	6.37
232011_s_at	MAP1LC3A	microtubule-associated protein 1 light chain 3 alpha	1.07 × 10^−13^	2.60	220773_s_at	GPHN	gephyrin	9.51 × 10^−19^	2.13
208786_s_at	MAP1LC3B	microtubule-associated protein 1 light chain 3 beta	5.66 × 10^−32^	2.26	223319_at	GPHN	gephyrin	7.91 × 10^−18^	2.06
205442_at	MFAP3L	microfibrillar-associated protein 3-like	6.52 × 10^−14^	6.61	202962_at	KIF13B	kinesin family member 13B	1.09 × 10^−19^	2.74
204631_at	MYH2	myosin, heavy chain 2, skeletal muscle, adult	7.89 × 10^−8^	6.38	226003_at	KIF21A	kinesin family member 21A	2.27 × 10^−25^	2.33
201058_s_at	MYL9	myosin, light chain 9, regulatory	1.46 × 10^−7^	2.00	231875_at	KIF21A	kinesin family member 21A	1.40 × 10^−16^	2.21
228098_s_at	MYLIP	myosin regulatory light chain interacting protein	5.37 × 10^−27^	7.57	225613_at	MAST4	Microtubule associated serine/threonine kinase family member 4	2.87 × 10^−16^	3.50
223130_s_at	MYLIP	myosin regulatory light chain interacting protein	1.59 × 10^−28^	7.13	225611_at	MAST4	Microtubule associated serine/threonine kinase family member 4	6.91 × 10^−15^	2.95
220319_s_at	MYLIP	myosin regulatory light chain interacting protein	7.00 × 10^−16^	2.93	40016_g_at	MAST4	microtubule associated serine/threonine kinase family member 4	5.49 × 10^−15^	2.43
223129_x_at	MYLIP	myosin regulatory light chain interacting protein	1.25 × 10^−14^	2.03	213511_s_at	MTMR1	myotubularin related protein 1	2.10 × 10^−20^	2.23
202555_s_at	MYLK	myosin light chain kinase	2.64 × 10^−5^	2.37	216095_x_at	MTMR1	myotubularin related protein 1	4.13 × 10^−21^	2.09
224823_at	MYLK	myosin light chain kinase	3.68 × 10^−5^	2.11	237206_at	MYOCD	myocardin	7.88 × 10^−3^	2.36
212338_at	MYO1D	myosin ID	3.13 × 10^−14^	8.06	219073_s_at	OSBPL10	oxysterol binding protein-like 10	5.46 × 10^−8^	2.27
223464_at	OSBPL5	oxysterol binding protein-like 5	1.87 × 10^−17^	2.26	219938_s_at	PSTPIP2	proline-serine-threonine phosphatase interacting protein 2	9.14 × 10^−13^	2.08
209019_s_at	PINK1	PTEN induced putative kinase 1	2.23 × 10^−26^	2.64	223471_at	RAB3IP	RAB3A interacting protein (rabin3)	4.80 × 10^−24^	4.06
209018_s_at	PINK1	PTEN induced putative kinase 1	8.46 × 10^−26^	2.62	219045_at	RHOF	ras homolog gene family, member F (in filopodia)	3.42 × 10^−18^	3.29
226627_at	SEPT8	septin 8	1.33 × 10^−9^	2.22	219263_at	RNF128	ring finger protein 128	3.33 × 10^−3^	2.11
230730_at	SGCD	sarcoglycan, delta (35 kDa dystrophin-associated glycoprotein)	3.81 × 10^−13^	10.01	204967_at	SHROOM2	shroom family member 2	2.29 × 10^−14^	15.26
213543_at	SGCD	sarcoglycan, delta (35 kDa dystrophin-associated glycoprotein)	4.91 × 10^−13^	8.70	213109_at	TNIK	TRAF2 and NCK interacting kinase	8.89 × 10^−6^	2.75
228602_at	SGCD	sarcoglycan, delta (35 kDa dystrophin-associated glycoprotein)	1.66 × 10^−8^	5.55	213107_at	TNIK	TRAF2 and NCK interacting kinase	1.18 × 10^−4^	2.51
210330_at	SGCD	sarcoglycan, delta (35 kDa dystrophin-associated glycoprotein)	1.25 × 10^−12^	4.80	216251_s_at	TTLL12	tubulin tyrosine ligase-like family, member 12	1.63 × 10^−17^	2.07
210329_s_at	SGCD	sarcoglycan, delta (35 kDa dystrophin-associated glycoprotein)	1.01 × 10^−10^	3.87	1552257_a_at	TTLL12	tubulin tyrosine ligase-like family, member 12	1.20 × 10^−18^	2.00
214492_at	SGCD	sarcoglycan, delta (35 kDa dystrophin-associated glycoprotein)	3.87 × 10^−7^	2.93	203702_s_at	TTLL4	tubulin tyrosine ligase-like family, member 4	3.28 × 10^−17^	2.24
207302_at	SGCG	sarcoglycan, gamma (35 kDa dystrophin-associated glycoprotein)	6.41 × 10^−28^	38.26					
228400_at	SHROOM3	shroom family member 3	1.15 × 10^−24^	15.85					
225548_at	SHROOM3	shroom family member 3	2.47 × 10^−24^	11.91					
217678_at	SLC7A11	solute carrier family 7, (cationic amino acid transporter, y+ system) member 11	1.52 × 10^−7^	2.36					
203516_at	SNTA1	syntrophin, alpha 1 (dystrophin-associated protein A1, 59 kDa, acidic component)	1.56 × 10^−19^	2.89					
201061_s_at	STOM	stomatin	2.06 × 10^−23^	2.38					
209306_s_at	SWAP70	SWAP-70 protein	3.61 × 10^−37^	2.22					
209307_at	SWAP70	SWAP-70 protein	3.18 × 10^−25^	2.07					
209904_at	TNNC1	troponin C type 1 (slow)	6.23 × 10^−5^	2.12					
238688_at	TPM1	Tropomyosin 1 (alpha)	1.82 × 10^−9^	2.70					
206117_at	TPM1	tropomyosin 1 (alpha)	6.69 × 10^−11^	2.33					
202479_s_at	TRIB2	tribbles homolog 2 (Drosophila)	1.37 × 10^−11^	2.20					
202478_at	TRIB2	tribbles homolog 2 (Drosophila)	3.53 × 10^−10^	2.09					
213908_at	WHAMML1 /2	WAS protein homolog associated with actin, golgi membranes and microtubules-like 1 /2	4.06 × 10^−21^	3.52					
1557261_at	WHAMML1 /2	WAS protein homolog associated with actin, golgi membranes and microtubules-like 1 /2	2.06 × 10^−15^	3.26					
**Cytoskeleton**									
**Probe Set ID**	**Gene Symbol**	**Gene Title**	**adj-pval**	**FC**	**Probe Set ID**	**Gene Symbol**	**Gene Title**	**adj-pval**	**FC**
205132_at	ACTC1	actin, alpha, cardiac muscle 1	1.23 × 10^−34^	78.69	202274_at	ACTG2	actin, gamma 2, smooth muscle, enteric	6.12 × 10^−10^	13.98
220115_s_at	CDH10	cadherin 10, type 2 (T2-cadherin)	1.12 × 10^−7^	3.11	210517_s_at	AKAP12	A kinase (PRKA) anchor protein 12	6.41 × 10^−6^	3.58
205532_s_at	CDH6	cadherin 6, type 2, K-cadherin (fetal kidney)	1.02 × 10^−2^	2.36	227529_s_at	AKAP12	A kinase (PRKA) anchor protein 12	1.17 × 10^−2^	2.42
232898_at	DAB2	disabled homolog 2, mitogen-responsive phosphoprotein (Drosophila)	8.32 × 10^−10^	3.32	227530_at	AKAP12	A kinase (PRKA) anchor protein 12	6.85 × 10^−3^	2.41
201279_s_at	DAB2	disabled homolog 2, mitogen-responsive phosphoprotein (Drosophila)	1.60 × 10^−18^	2.30	206298_at	ARHGAP22	Rho GTPase activating protein 22	4.33 × 10^−30^	4.16
201280_s_at	DAB2	disabled homolog 2, mitogen-responsive phosphoprotein (Drosophila)	3.78 × 10^−21^	2.28	201005_at	CD9	CD9 molecule	7.48 × 10^−6^	2.96
201278_at	DAB2	disabled homolog 2, mitogen-responsive phosphoprotein (Drosophila)	1.77 × 10^−23^	2.26	214297_at	CSPG4	chondroitin sulfate proteoglycan 4	8.70 × 10^−5^	2.37
210757_x_at	DAB2	disabled homolog 2, mitogen-responsive phosphoprotein (Drosophila)	4.14 × 10^−18^	2.17	220512_at	DLC1	deleted in liver cancer 1	2.59 × 10^−15^	3.03
240873_x_at	DAB2	disabled homolog 2, mitogen-responsive phosphoprotein (Drosophila)	1.06 × 10^−13^	2.08	211478_s_at	DPP4	dipeptidyl-peptidase 4	2.91 × 10^−19^	11.71
214724_at	DIXDC1	DIX domain containing 1	1.31 × 10^−9^	2.20	203716_s_at	DPP4	dipeptidyl-peptidase 4	2.07 × 10^−21^	10.11
224814_at	DPP7	dipeptidyl-peptidase 7	1.13 × 10^−21^	2.61	203717_at	DPP4	dipeptidyl-peptidase 4	9.35 × 10^−19^	6.79
205031_at	EFNB3	ephrin-B3	1.05 × 10^−9^	2.43	217901_at	DSG2	desmoglein 2	1.05 × 10^−10^	19.57
208228_s_at	FGFR2	fibroblast growth factor receptor 2	9.30 × 10^−10^	3.71	1553105_s_at	DSG2	desmoglein 2	4.29 × 10^−9^	7.96
204379_s_at	FGFR3	fibroblast growth factor receptor 3	1.66 × 10^−19^	4.70	227955_s_at	EFNA5	ephrin-A5	1.28 × 10^−10^	4.10
201539_s_at	FHL1	four and a half LIM domains 1	2.10 × 10^−4^	2.76	214036_at	EFNA5	ephrin-A5	1.68 × 10^−7^	2.33
214505_s_at	FHL1	four and a half LIM domains 1	6.14 × 10^−4^	2.37	202669_s_at	EFNB2	ephrin-B2	2.57 × 10^−2^	2.16
210299_s_at	FHL1	four and a half LIM domains 1	2.65 × 10^−4^	2.29	201983_s_at	EGFR	epidermal growth factor receptor	3.46 × 10^−10^	2.10
210298_x_at	FHL1	four and a half LIM domains 1	6.34 × 10^−4^	2.27	218796_at	FERMT1	fermitin family homolog 1 (Drosophila)	2.45 × 10^−12^	4.17
208748_s_at	FLOT1	flotillin 1	5.72 × 10^−7^	3.13	60474_at	FERMT1	fermitin family homolog 1 (Drosophila)	9.46 × 10^−13^	3.84
222899_at	ITGA11	integrin, alpha 11	1.08 × 10^−21^	26.89	242422_at	G3BP1	GTPase activating protein (SH3 domain) binding protein 1	3.82 × 10^−5^	2.28
215177_s_at	ITGA6	integrin, alpha 6	9.34 × 10^−11^	6.34	206383_s_at	G3BP2	GTPase activating protein (SH3 domain) binding protein 2	1.94 × 10^−17^	2.17
201656_at	ITGA6	integrin, alpha 6	5.30 × 10^−11^	4.58	206074_s_at	HMGA1	high mobility group AT-hook 1	8.66 × 10^−22^	2.89
214265_at	ITGA8	integrin, alpha 8	7.06 × 10^−7^	4.79	208025_s_at	HMGA2	high mobility group AT-hook 2	5.89 × 10^−29^	22.82
227297_at	ITGA9	integrin, alpha 9	6.70 × 10^−8^	2.35	1567224_at	HMGA2	high mobility group AT-hook 2	2.78 × 10^−21^	4.79
202803_s_at	ITGB2	integrin, beta 2 (complement component 3 receptor 3 and 4 subunit)	9.97 × 10^−9^	2.63	1558683_a_at	HMGA2	high mobility group AT-hook 2	3.31 × 10^−25^	4.32
226189_at	ITGB8	integrin, beta 8	8.02 × 10^−14^	6.70	1561633_at	HMGA2	high mobility group AT-hook 2	4.45 × 10^−22^	3.95
205422_s_at	ITGBL1	integrin, beta-like 1 (with EGF-like repeat domains)	2.20 × 10^−16^	12.03	1558682_at	HMGA2	high mobility group AT-hook 2	3.32 × 10^−22^	2.27
231993_at	ITGBL1	Integrin, beta-like 1 (with EGF-like repeat domains)	1.41 × 10^−15^	10.90	202638_s_at	ICAM1	intercellular adhesion molecule 1	3.48 × 10^−5^	2.74
214927_at	ITGBL1	integrin, beta-like 1 (with EGF-like repeat domains)	2.34 × 10^−13^	8.91	202637_s_at	ICAM1	intercellular adhesion molecule 1	6.68 × 10^−8^	2.33
1557080_s_at	ITGBL1	integrin, beta-like 1 (with EGF-like repeat domains)	1.48 × 10^−12^	8.22	213620_s_at	ICAM2	intercellular adhesion molecule 2	3.73 × 10^−13^	2.64
1557079_at	ITGBL1	Integrin, beta-like 1 (with EGF-like repeat domains)	3.54 × 10^−16^	7.56	213446_s_at	IQGAP1	IQ motif containing GTPase activating protein 1	1.13 × 10^−6^	2.08
228080_at	LAYN	layilin	8.17 × 10^−10^	2.89	206766_at	ITGA10	integrin, alpha 10	2.50 × 10^−6^	4.61
220765_s_at	LIMS2	LIM and senescent cell antigen-like domains 2	1.13 × 10^−10^	2.99	227314_at	ITGA2	integrin, alpha 2 (CD49B, alpha 2 subunit of VLA-2 receptor)	6.15 × 10^−8^	3.59
202674_s_at	LMO7	LIM domain 7	4.31 × 10^−14^	2.85	205032_at	ITGA2	integrin, alpha 2 (CD49B, alpha 2 subunit of VLA-2 receptor)	4.90 × 10^−7^	2.82
242722_at	LMO7	LIM domain 7	4.41 × 10^−10^	2.49	204627_s_at	ITGB3	integrin, beta 3 (platelet glycoprotein IIIa, antigen CD61)	9.77 × 10^−9^	2.61
213490_s_at	MAP2K2	mitogen-activated protein kinase kinase 2	7.78 × 10^−5^	2.09	223800_s_at	LIMS3	LIM and senescent cell antigen-like domains 3	4.73 × 10^−19^	4.89
213438_at	NFASC	neurofascin homolog (chicken)	1.78 × 10^−16^	16.35	209615_s_at	PAK1	p21 protein (Cdc42/Rac)-activated kinase 1	3.17 × 10^−15^	2.06
230242_at	NFASC	neurofascin homolog (chicken)	2.51 × 10^−12^	3.55	228635_at	PCDH10	protocadherin 10	2.29 × 10^−11^	16.71
243645_at	NFASC	neurofascin homolog (chicken)	6.86 × 10^−15^	2.84	205534_at	PCDH7	protocadherin 7	2.49 × 10^−4^	3.81
222455_s_at	PARVA	parvin, alpha	5.02 × 10^−28^	2.01	228640_at	PCDH7	protocadherin 7	7.79 × 10^−3^	2.84
37965_at	PARVB	parvin, beta	8.71 × 10^−9^	2.05	205535_s_at	PCDH7	protocadherin 7	6.91 × 10^−5^	2.57
225977_at	PCDH18	protocadherin 18	2.01 × 10^−24^	7.05	219737_s_at	PCDH9	protocadherin 9	2.28 × 10^−3^	2.60
225975_at	PCDH18	protocadherin 18	5.95 × 10^−23^	5.41	238419_at	PHLDB2	pleckstrin homology-like domain, family B, member 2	1.23 × 10^−8^	3.41
223854_at	PCDHB10	protocadherin beta 10	2.58 × 10^−11^	2.37	214374_s_at	PPFIBP1	PTPRF interacting protein, binding protein 1 (liprin beta 1)	5.08 × 10^−8^	2.07
232099_at	PCDHB16	protocadherin beta 16	7.07 × 10^−15^	3.65	203650_at	PROCR	protein C receptor, endothelial (EPCR)	1.20 × 10^−14^	2.89
231725_at	PCDHB2	protocadherin beta 2	1.82 × 10^−30^	10.23	216915_s_at	PTPN12	protein tyrosine phosphatase, non-receptor type 12	2.10 × 10^−8^	2.00
212841_s_at	PPFIBP2	PTPRF interacting protein, binding protein 2 (liprin beta 2)	9.85 × 10^−32^	4.48	202565_s_at	SVIL	supervillin	3.65 × 10^−6^	2.95
207011_s_at	PTK7	PTK7 protein tyrosine kinase 7	1.50 × 10^−6^	2.06	206702_at	TEK	TEK tyrosine kinase, endothelial	3.34 × 10^−15^	13.28
227557_at	SCARF2	scavenger receptor class F, member 2	1.76 × 10^−18^	2.42	223314_at	TSPAN14	tetraspanin 14	1.52 × 10^−15^	2.71
212154_at	SDC2	syndecan 2	1.02 × 10^−14^	2.45	221002_s_at	TSPAN14	tetraspanin 14	2.48 × 10^−15^	2.01
212157_at	SDC2	syndecan 2	3.60 × 10^−12^	2.16	209890_at	TSPAN5	tetraspanin 5	5.39 × 10^−26^	2.00
212158_at	SDC2	syndecan 2	7.05 × 10^−13^	2.10	203868_s_at	VCAM1	vascular cell adhesion molecule 1	2.90 × 10^−4^	4.10
202898_at	SDC3	syndecan 3	2.33 × 10^−9^	2.98					
226438_at	SNTB1	syntrophin, beta 1 (dystrophin-associated protein A1, 59 kDa, basic component 1)	2.90 × 10^−4^	2.11					
218087_s_at	SORBS1	sorbin and SH3 domain containing 1	2.49 × 10^−4^	3.11					
222513_s_at	SORBS1	sorbin and SH3 domain containing 1	4.61 × 10^−3^	2.00					
225728_at	SORBS2	sorbin and SH3 domain containing 2	9.66 × 10^−4^	2.52					
204288_s_at	SORBS2	sorbin and SH3 domain containing 2	1.01 × 10^−3^	2.10					
202796_at	SYNPO	synaptopodin	8.58 × 10^−4^	2.06					
225720_at	SYNPO2	synaptopodin 2	8.30 × 10^−16^	15.40					
225895_at	SYNPO2	synaptopodin 2	6.69 × 10^−16^	9.94					
225721_at	SYNPO2	synaptopodin 2	2.22 × 10^−16^	9.02					
225894_at	SYNPO2	synaptopodin 2	4.14 × 10^−15^	4.97					
40837_at	TLE2	transducin-like enhancer of split 2 (E(sp1) homolog, Drosophila)	1.54 × 10^−15^	3.71					
221747_at	TNS1	tensin 1	4.25 × 10^−10^	2.00					
227307_at	TSPAN18	Tetraspanin 18	2.81 × 10^−9^	4.58					
227236_at	TSPAN2	tetraspanin 2	4.44 × 10^−6^	3.18					
209264_s_at	TSPAN4	tetraspanin 4	3.72 × 10^−11^	2.46					
**LINC Complexes**									
**Probe Set ID**	**Gene Symbol**	**Gene Title**	**adj-pval**	**FC**	**Probe Set ID**	**Gene Symbol**	**Gene Title**	**adj-pval**	**FC**
209230_s_at	NUPR1	nuclear protein 1	2.43 × 10^−15^	12.15	206550_s_at	NUP155	nucleoporin 155 kDa	1.03 × 10^−15^	2.04
219888_at	SPAG4	sperm associated antigen 4	1.16 × 10^−9^	3.27	225470_at	NUP35	nucleoporin 35 kDa	1.38 × 10^−21^	2.06
232027_at	SYNE1	Spectrin repeat containing, nuclear envelope 1	1.61 × 10^−17^	6.92					
209447_at	SYNE1	spectrin repeat containing, nuclear envelope 1	5.46 × 10^−12^	2.25					
**Nucleoskeleton**									
**Probe Set ID**	**Gene Symbol**	**Gene Title**	**adj-pval**	**FC**	**Probe Set ID**	**Gene Symbol**	**Gene Title**	**adj-pval**	**FC**
215071_s_at	HIST1H2AC	histone cluster 1, H2ac	8.12 × 10^−9^	2.61	227048_at	LAMA1	laminin, alpha 1	7.19 × 10^−11^	4.99
209911_x_at	HIST1H2BD	histone cluster 1, H2bd	1.44 × 10^−7^	2.08	211651_s_at	LAMB1	laminin, beta 1	2.68 × 10^−21^	2.83
214290_s_at	HIST2H2AA3 HIST2H2AA4	histone cluster 2, H2aa3 histone cluster 2, H2aa4	1.07 × 10^−10^	2.38	201505_at	LAMB1	laminin, beta 1	6.03 × 10^−20^	2.43
218280_x_at	HIST2H2AA3 HIST2H2AA4	histone cluster 2, H2aa3 histone cluster 2, H2aa4	8.30 × 10^−11^	2.22	242918_at	NASP	Nuclear autoantigenic sperm protein (histone-binding)	1.21 × 10^−4^	2.15
202708_s_at	HIST2H2BE	histone cluster 2, H2be	1.53 × 10^−8^	2.20	201970_s_at	NASP	nuclear autoantigenic sperm protein (histone-binding)	2.90 × 10^−16^	2.04
221582_at	HIST3H2A	histone cluster 3, H2a	1.51 × 10^−19^	2.39	209754_s_at	TMPO	thymopoietin	7.79 × 10^−15^	2.99
205116_at	LAMA2	laminin, alpha 2	1.03 × 10^−10^	4.64	209753_s_at	TMPO	thymopoietin	3.32 × 10^−8^	2.02
216840_s_at	LAMA2	laminin, alpha 2	4.12 × 10^−10^	4.36					
213519_s_at	LAMA2	laminin, alpha 2	1.78 × 10^−10^	3.97					
202202_s_at	LAMA4	laminin, alpha 4	7.35 × 10^−6^	5.39					
210089_s_at	LAMA4	laminin, alpha 4	1.39 × 10^−7^	3.10					
216264_s_at	LAMB2	laminin, beta 2 (laminin S)	1.72 × 10^−26^	2.94					
**Secreted Factors**									
**Probe Set ID**	**Gene Symbol**	**Gene Title**	**adj-pval**	**FC**	**Probe Set ID**	**Gene Symbol**	**Gene Title**	**adj-pval**	**FC**
229819_at	A1BG	alpha-1-B glycoprotein	1.21 × 10^−14^	2.11	204694_at	AFP	alpha-fetoprotein	2.77 × 10^−12^	2.11
202912_at	ADM	adrenomedullin	9.27 × 10^−10^	2.59	221009_s_at	ANGPTL4	angiopoietin-like 4	4.26 × 10^−25^	21.5
205141_at	ANG	angiogenin, ribonuclease, RNase A family, 5	7.82 × 10^−13^	2.35	223333_s_at	ANGPTL4	angiopoietin-like 4	1.89 × 10^−21^	11.7
213001_at	ANGPTL2	angiopoietin-like 2	5.71 × 10^−23^	7.43	205239_at	AREG	amphiregulin	9.14 × 10^−7^	2.28
213004_at	ANGPTL2	angiopoietin-like 2	9.64 × 10^−26^	4.96	211518_s_at	BMP4	bone morphogenetic protein 4	6.50 × 10^−3^	2.09
219514_at	ANGPTL2	angiopoietin-like 2	4.12 × 10^−22^	2.90	209301_at	CA2	carbonic anhydrase II	1.60 × 10^−6^	8.34
238987_at	B4GALT1	UDP-Gal:betaGlcNAc beta 1,4- galactosyltransferase, polypeptide 1	1.32 × 10^−8^	2.15	216598_s_at	CCL2	chemokine (C-C motif) ligand 2	7.24 × 10^−8^	6.16
206176_at	BMP6	bone morphogenetic protein 6	9.19 × 10^−5^	2.75	205476_at	CCL20	chemokine (C-C motif) ligand 20	2.08 × 10^−6^	2.24
220988_s_at	C1QTNF3	C1q and tumor necrosis factor related protein 3	7.54 × 10^−9^	2.28	208075_s_at	CCL7	chemokine (C-C motif) ligand 7	2.99 × 10^−9^	2.30
223499_at	C1QTNF5 MFRP	C1q and tumor necrosis factor related protein 5 /membrane frizzled-related protein	2.40 × 10^−25^	8.01	215388_s_at	CFH CFHR1	complement factor H complement factor H-related 1	1.27 × 10^−3^	2.82
235221_at	CBLN3	cerebellin 3 precursor	5.03 × 10^−12^	2.22	209395_at	CHI3L1	chitinase 3-like 1 (cartilage glycoprotein-39)	9.49 × 10^−5^	5.50
209616_s_at	CES1	carboxylesterase 1 (monocyte/macrophage serine esterase 1)	1.15 × 10^−4^	2.88	209396_s_at	CHI3L1	chitinase 3-like 1 (cartilage glycoprotein-39)	3.39 × 10^−4^	3.69
205382_s_at	CFD	complement factor D (adipsin)	2.42 × 10^−18^	5.81	235099_at	CMTM8	CKLF-like MARVEL transmembrane domain containing 8	7.13 × 10^−5^	2.02
200884_at	CKB	creatine kinase, brain	2.07 × 10^−31^	6.59	205832_at	CPA4	carboxypeptidase A4	5.82 × 10^−3^	2.61
201117_s_at	CPE	carboxypeptidase E	3.74 × 10^−32^	34.67	204470_at	CXCL1	chemokine (C-X-C motif) ligand 1	2.10 × 10^−8^	9.57
201116_s_at	CPE	carboxypeptidase E	9.98 × 10^−32^	26.80	209774_x_at	CXCL2	chemokine (C-X-C motif) ligand 2	1.84 × 10^−13^	14.5
206100_at	CPM	carboxypeptidase M	7.56 × 10^−6^	2.80	207850_at	CXCL3	chemokine (C-X-C motif) ligand 3	3.30 × 10^−17^	15.1
201200_at	CREG1	cellular repressor of E1A-stimulated genes 1	1.40 × 10^−10^	2.97	214974_x_at	CXCL5	chemokine (C-X-C motif) ligand 5	5.88 × 10^−11^	12.1
201360_at	CST3	cystatin C	6.04 × 10^−15^	2.23	215101_s_at	CXCL5	chemokine (C-X-C motif) ligand 5	5.26 × 10^−10^	8.60
206595_at	CST6	cystatin E/M	6.70 × 10^−13^	7.45	206336_at	CXCL6	chemokine (C-X-C motif) ligand 6 (granulocyte chemotactic protein 2)	2.59 × 10^−4^	5.46
209687_at	CXCL12	chemokine (C-X-C motif) ligand 12 (stromal cell-derived factor 1)	2.68 × 10^−10^	8.12	213092_x_at	DNAJC9	DnaJ (Hsp40) homolog, subfamily C, member 9	5.56 × 10^−13^	2.08
203666_at	CXCL12	chemokine (C-X-C motif) ligand 12 (stromal cell-derived factor 1)	1.08 × 10^−7^	6.47	201430_s_at	DPYSL3	dihydropyrimidinase-like 3	3.53 × 10^−11^	2.69
222484_s_at	CXCL14	chemokine (C-X-C motif) ligand 14	2.55 × 10^−14^	8.48	201431_s_at	DPYSL3	dihydropyrimidinase-like 3	1.07 × 10^−11^	2.61
218002_s_at	CXCL14	chemokine (C-X-C motif) ligand 14	1.02 × 10^−13^	7.50	206254_at	EGF	epidermal growth factor (beta-urogastrone)	1.25 × 10^−7^	2.37
212977_at	CXCR7	chemokine (C-X-C motif) receptor 7	2.78 × 10^−13^	17.10	1559072_a_at	ELFN2	extracellular leucine-rich repeat and fibronectin type III domain containing 2	2.43 × 10^−8^	2.17
232746_at	CXCR7	Chemokine (C-X-C motif) receptor 7	4.11 × 10^−6^	2.28	205767_at	EREG	epiregulin	1.96 × 10^−10^	5.08
222996_s_at	CXXC5	CXXC finger 5	1.25 × 10^−9^	2.33	208378_x_at	FGF5	fibroblast growth factor 5	2.27 × 10^−12^	2.17
233955_x_at	CXXC5	CXXC finger 5	2.25 × 10^−9^	2.29	210310_s_at	FGF5	fibroblast growth factor 5	5.16 × 10^−11^	2.13
224516_s_at	CXXC5	CXXC finger 5	1.17 × 10^−8^	2.19	206614_at	GDF5	growth differentiation factor 5	1.17 × 10^−17^	6.75
207169_x_at	DDR1	discoidin domain receptor tyrosine kinase 1	2.84 × 10^−10^	2.06	38037_at	HBEGF	heparin-binding EGF-like growth factor	1.51 × 10^−9^	3.17
204602_at	DKK1	dickkopf homolog 1 (Xenopus laevis)	9.91 × 10^−7^	2.54	203821_at	HBEGF	heparin-binding EGF-like growth factor	2.98 × 10^−9^	3.15
202196_s_at	DKK3	dickkopf homolog 3 (Xenopus laevis)	8.90 × 10^−10^	4.17	209960_at	HGF	hepatocyte growth factor (hepapoietin A; scatter factor)	6.82 × 10^−10^	8.20
221127_s_at	DKK3	dickkopf homolog 3 (Xenopus laevis)	2.77 × 10^−11^	3.45	210997_at	HGF	hepatocyte growth factor (hepapoietin A; scatter factor)	2.11 × 10^−6^	4.34
214247_s_at	DKK3	dickkopf homolog 3 (Xenopus laevis)	1.33 × 10^−8^	2.71	210998_s_at	HGF	hepatocyte growth factor (hepapoietin A; scatter factor)	1.82 × 10^−4^	2.28
230508_at	DKK3	dickkopf homolog 3 (Xenopus laevis)	8.73 × 10^−5^	2.07	206924_at	IL11	interleukin 11	6.19 × 10^−6^	2.45
222802_at	EDN1	endothelin 1	8.74 × 10^−7^	4.31	210118_s_at	IL1A	interleukin 1, alpha	4.81 × 10^−4^	2.87
218995_s_at	EDN1	endothelin 1	3.17 × 10^−6^	4.06	205067_at	IL1B	interleukin 1, beta	1.25 × 10^−5^	5.09
227708_at	EEF1A1	eukaryotic translation elongation factor 1 alpha 1	1.54 × 10^−14^	2.03	39402_at	IL1B	interleukin 1, beta	4.60 × 10^−5^	4.09
201313_at	ENO2	enolase 2 (gamma, neuronal)	2.61 × 10^−8^	2.11	209821_at	IL33	interleukin 33	5.24 × 10^−5^	2.71
210839_s_at	ENPP2	ectonucleotide pyrophosphatase/phosphodiesterase 2	9.19 × 10^−10^	12.01	204863_s_at	IL6ST	interleukin 6 signal transducer (gp130, oncostatin M receptor)	1.19 × 10^−8^	2.33
209392_at	ENPP2	ectonucleotide pyrophosphatase/phosphodiesterase 2	2.29 × 10^−10^	10.40	211000_s_at	IL6ST	interleukin 6 signal transducer (gp130, oncostatin M receptor)	3.36 × 10^−8^	2.17
205756_s_at	F8	coagulation factor VIII, procoagulant component	2.18 × 10^−8^	2.13	202859_x_at	IL8	interleukin 8	4.66 × 10^−8^	8.46
226722_at	FAM20C	family with sequence similarity 20, member C	3.87 × 10^−7^	2.58	211506_s_at	IL8	interleukin 8	2.57 × 10^−6^	5.63
205110_s_at	FGF13	fibroblast growth factor 13	7.54 × 10^−9^	3.02	204926_at	INHBA	inhibin, beta A	1.82 × 10^−14^	4.83
204422_s_at	FGF2	fibroblast growth factor 2 (basic)	6.77 × 10^−19^	2.29	210511_s_at	INHBA	inhibin, beta A	6.98 × 10^−8^	4.63
205782_at	FGF7	fibroblast growth factor 7 (keratinocyte growth factor)	1.37 × 10^−11^	10.94	205266_at	LIF	leukemia inhibitory factor (cholinergic differentiation factor)	4.28 × 10^−10^	6.74
1554741_s_at	FGF7 KGFLP1 KGFLP2	fibroblast growth factor 7 (keratinocyte growth factor) keratinocyte growth factor-like protein 1 keratinocyte growth factor-like protein 2	6.50 × 10^−11^	7.49	205381_at	LRRC17	leucine rich repeat containing 17	5.79 × 10^−13^	42.4
206404_at	FGF9	fibroblast growth factor 9 (glia-activating factor)	1.50 × 10^−5^	2.74	207703_at	NLGN4Y	neuroligin 4, Y-linked	6.00 × 10^−9^	5.96
209093_s_at	GBA GBAP	glucosidase, beta; acid (includes glucosylceramidase) glucosidase, beta; acid, pseudogene	1.35 × 10^−18^	2.01	229838_at	NUCB2	nucleobindin 2	4.07 × 10^−22^	2.21
205498_at	GHR	growth hormone receptor	5.70 × 10^−6^	2.11	216867_s_at	PDGFA	platelet-derived growth factor alpha polypeptide	4.78 × 10^−10^	3.93
220794_at	GREM2	gremlin 2, cysteine knot superfamily, homolog (Xenopus laevis)	1.54 × 10^−29^	52.50	205463_s_at	PDGFA	platelet-derived growth factor alpha polypeptide	3.82 × 10^−10^	3.83
240509_s_at	GREM2	gremlin 2, cysteine knot superfamily, homolog (Xenopus laevis)	3.80 × 10^−33^	45.90	221898_at	PDPN	podoplanin	2.45 × 10^−8^	3.21
235504_at	GREM2	gremlin 2, cysteine knot superfamily, homolog (Xenopus laevis)	2.59 × 10^−33^	37.93	204879_at	PDPN	podoplanin	8.06 × 10^−7^	2.10
216041_x_at	GRN	granulin	4.12 × 10^−26^	2.77	218454_at	PLBD1	phospholipase B domain containing 1	7.38 × 10^−7^	2.70
200678_x_at	GRN	granulin	8.63 × 10^−27^	2.60	213449_at	POP1	processing of precursor 1, ribonuclease P/MRP subunit (S. cerevisiae)	6.40 × 10^−12^	2.14
211284_s_at	GRN	granulin	1.04 × 10^−26^	2.45	213421_x_at	PRSS3	protease, serine, 3	9.97 × 10^−4^	2.31
206326_at	GRP	gastrin-releasing peptide	1.71 × 10^−5^	2.37	207463_x_at	PRSS3	protease, serine, 3	2.59 × 10^−3^	2.17
204773_at	IL11RA	interleukin 11 receptor, alpha	9.87 × 10^−12^	2.32	206631_at	PTGER2	prostaglandin E receptor 2 (subtype EP2), 53 kDa	2.43 × 10^−9^	2.96
206295_at	IL18	interleukin 18 (interferon-gamma-inducing factor)	6.66 × 10^−3^	2.15	204897_at	PTGER4	prostaglandin E receptor 4 (subtype EP4)	1.62 × 10^−5^	4.35
202948_at	IL1R1	interleukin 1 receptor, type I	2.88 × 10^−10^	2.33	227146_at	QSOX2	quiescin Q6 sulfhydryl oxidase 2	1.21 × 10^−23^	2.14
228575_at	IL20RB	interleukin 20 receptor beta	3.21 × 10^−33^	20.03	204916_at	RAMP1	receptor (G protein-coupled) activity modifying protein 1	5.83 × 10^−5^	2.06
221658_s_at	IL21R	interleukin 21 receptor	5.81 × 10^−10^	2.67	219140_s_at	RBP4	retinol binding protein 4, plasma	1.87 × 10^−4^	2.28
226333_at	IL6R	interleukin 6 receptor	1.33 × 10^−9^	2.61	206805_at	SEMA3A	sema domain, immunoglobulin domain (Ig), short basic domain, secreted, (semaphorin) 3A	1.29 × 10^−5^	2.54
206693_at	IL7	interleukin 7	7.42 × 10^−8^	2.00	244163_at	SEMA3A	sema domain, immunoglobulin domain (Ig), short basic domain, secreted, (semaphorin) 3A	4.25 × 10^−7^	2.02
226218_at	IL7R	interleukin 7 receptor	4.57 × 10^−14^	12.12	230345_at	SEMA7A	semaphorin 7A, GPI membrane anchor (John Milton Hagen blood group)	5.22 × 10^−8^	2.70
205798_at	IL7R	interleukin 7 receptor	5.98 × 10^−15^	10.35	209723_at	SERPINB9	serpin peptidase inhibitor, clade B (ovalbumin), member 9	1.23 × 10^−7^	4.73
205258_at	INHBB	inhibin, beta B	4.04 × 10^−7^	6.33	205576_at	SERPIND1	serpin peptidase inhibitor, clade D (heparin cofactor), member 1	1.39 × 10^−6^	2.04
205051_s_at	KIT	v-kit Hardy-Zuckerman 4 feline sarcoma viral oncogene homolog	1.65 × 10^−13^	6.91	213600_at	SIPA1L3	signal-induced proliferation-associated 1 like 3	4.01 × 10^−23^	2.39
207092_at	LEP	leptin	4.09 × 10^−5^	2.83	37831_at	SIPA1L3	signal-induced proliferation-associated 1 like 3	3.40 × 10^−25^	2.07
206584_at	LY96	lymphocyte antigen 96	1.50 × 10^−19^	2.46	204466_s_at	SNCA	synuclein, alpha (non A4 component of amyloid precursor)	8.83 × 10^−6^	3.50
232224_at	MASP1	mannan-binding lectin serine peptidase 1 (C4/C2 activating component of Ra-reactive factor)	4.44 × 10^−7^	5.65	201562_s_at	SORD	sorbitol dehydrogenase	4.85 × 10^−15^	2.16
201621_at	NBL1	neuroblastoma, suppression of tumorigenicity 1	2.11 × 10^−9^	2.66	242408_at	STYX	serine/threonine/tyrosine interacting protein	3.00 × 10^−8^	2.02
37005_at	NBL1	neuroblastoma, suppression of tumorigenicity 1	1.16 × 10^−8^	2.33	209676_at	TFPI	tissue factor pathway inhibitor (lipoprotein-associated coagulation inhibitor)	4.68 × 10^−5^	2.43
205893_at	NLGN1	neuroligin 1	7.98 × 10^−3^	2.09	213258_at	TFPI	tissue factor pathway inhibitor (lipoprotein-associated coagulation inhibitor)	3.07 × 10^−5^	2.34
231361_at	NLGN1	Neuroligin 1	4.26 × 10^−3^	2.02	210664_s_at	TFPI	tissue factor pathway inhibitor (lipoprotein-associated coagulation inhibitor)	1.32 × 10^−4^	2.22
231798_at	NOG	noggin	2.87 × 10^−41^	97.99	235737_at	TSLP	thymic stromal lymphopoietin	5.21 × 10^−14^	6.10
206343_s_at	NRG1	neuregulin 1	4.21 × 10^−4^	2.02	213425_at	WNT5A	wingless-type MMTV integration site family, member 5A	1.06 × 10^−13^	4.23
218625_at	NRN1	neuritin 1	3.68 × 10^−15^	29.54	205990_s_at	WNT5A	wingless-type MMTV integration site family, member 5A	6.42 × 10^−14^	3.76
200649_at	NUCB1	nucleobindin 1	7.18 × 10^−12^	2.05	238105_x_at	WNT7B	wingless-type MMTV integration site family, member 7B	1.17 × 10^−11^	2.58
213131_at	OLFM1	olfactomedin 1	5.16 × 10^−35^	18.89					
205591_at	OLFM1	olfactomedin 1	0.00 × 10^+00^	15.89					
214620_x_at	PAM	peptidylglycine alpha-amidating monooxygenase	8.58 × 10^−23^	2.25					
202336_s_at	PAM	peptidylglycine alpha-amidating monooxygenase	1.74 × 10^−22^	2.25					
212958_x_at	PAM	peptidylglycine alpha-amidating monooxygenase	1.18 × 10^−21^	2.09					
219304_s_at	PDGFD	platelet derived growth factor D	1.83 × 10^−15^	9.33					
209652_s_at	PGF	placental growth factor	1.69 × 10^−6^	2.11					
201578_at	PODXL	podocalyxin-like	7.00 × 10^−20^	20.38					
207808_s_at	PROS1	protein S (alpha)	8.76 × 10^−9^	3.01					
200866_s_at	PSAP	prosaposin	2.12 × 10^−13^	2.29					
208257_x_at	PSG1	pregnancy specific beta-1-glycoprotein 1	7.51 × 10^−7^	6.45					
210195_s_at	PSG1	pregnancy specific beta-1-glycoprotein 1	1.76 × 10^−8^	3.21					
210196_s_at	PSG1	pregnancy specific beta-1-glycoprotein 1	3.24 × 10^−5^	2.18					
208134_x_at	PSG2	pregnancy specific beta-1-glycoprotein 2	4.46 × 10^−19^	5.98					
211741_x_at	PSG3	pregnancy specific beta-1-glycoprotein 3	3.72 × 10^−27^	28.52					
203399_x_at	PSG3	pregnancy specific beta-1-glycoprotein 3	4.42 × 10^−30^	25.95					
215821_x_at	PSG3	pregnancy specific beta-1-glycoprotein 3	1.34 × 10^−22^	7.87					
208191_x_at	PSG4	pregnancy specific beta-1-glycoprotein 4	6.32 × 10^−6^	8.81					
204830_x_at	PSG5	pregnancy specific beta-1-glycoprotein 5	9.42 × 10^−29^	200.65					
209738_x_at	PSG6	pregnancy specific beta-1-glycoprotein 6	4.68 × 10^−29^	78.75					
208106_x_at	PSG6	pregnancy specific beta-1-glycoprotein 6	2.35 × 10^−28^	51.80					
205602_x_at	PSG7	pregnancy specific beta-1-glycoprotein 7	1.27 × 10^−5^	4.78					
209594_x_at	PSG9	pregnancy specific beta-1-glycoprotein 9	1.83 × 10^−29^	89.31					
207733_x_at	PSG9	pregnancy specific beta-1-glycoprotein 9	5.66 × 10^−26^	12.06					
212187_x_at	PTGDS	prostaglandin D2 synthase 21 kDa (brain)	1.76 × 10^−5^	3.89					
211748_x_at	PTGDS	prostaglandin D2 synthase 21 kDa (brain)	6.16 × 10^−6^	3.30					
211663_x_at	PTGDS	prostaglandin D2 synthase 21 kDa (brain)	7.37 × 10^−4^	2.32					
213933_at	PTGER3	prostaglandin E receptor 3 (subtype EP3)	3.46 × 10^−7^	3.51					
1555097_a_at	PTGFR	prostaglandin F receptor (FP)	5.53 × 10^−8^	2.27					
207177_at	PTGFR	prostaglandin F receptor (FP)	1.00 × 10^−7^	2.21					
206187_at	PTGIR	prostaglandin I2 (prostacyclin) receptor (IP)	1.11 × 10^−13^	2.31					
208131_s_at	PTGIS	prostaglandin I2 (prostacyclin) synthase	2.20 × 10^−16^	42.66					
211892_s_at	PTGIS	prostaglandin I2 (prostacyclin) synthase	2.07 × 10^−8^	3.10					
210702_s_at	PTGIS	prostaglandin I2 (prostacyclin) synthase	7.89 × 10^−9^	2.99					
211756_at	PTHLH	parathyroid hormone-like hormone	8.63 × 10^−3^	2.07					
215253_s_at	RCAN1	regulator of calcineurin 1	1.52 × 10^−6^	2.47					
203498_at	RCAN2	regulator of calcineurin 2	1.23 × 10^−39^	49.77					
226272_at	RCAN3	RCAN family member 3	1.16 × 10^−12^	2.59					
213716_s_at	SECTM1	secreted and transmembrane 1	1.48 × 10^−4^	2.14					
226492_at	SEMA6D	sema domain, transmembrane domain (TM), and cytoplasmic domain, (semaphorin) 6D	1.30 × 10^−4^	2.71					
200986_at	SERPING1	serpin peptidase inhibitor, clade G (C1 inhibitor), member 1	2.81 × 10^−20^	13.38					
204596_s_at	STC1	stanniocalcin 1	1.03 × 10^−4^	2.89					
204595_s_at	STC1	stanniocalcin 1	3.40 × 10^−5^	2.12					
203438_at	STC2	stanniocalcin 2	1.40 × 10^−6^	2.84					
203439_s_at	STC2	stanniocalcin 2	9.48 × 10^−5^	2.27					
212344_at	SULF1	sulfatase 1	4.55 × 10^−12^	9.94					
212353_at	SULF1	sulfatase 1	5.41 × 10^−10^	8.59					
212354_at	SULF1	sulfatase 1	6.12 × 10^−11^	8.19					
224724_at	SULF2	sulfatase 2	2.31 × 10^−10^	3.82					
207426_s_at	TNFSF4	tumor necrosis factor (ligand) superfamily, member 4	1.47 × 10^−2^	2.59					
206907_at	TNFSF9	tumor necrosis factor (ligand) superfamily, member 9	2.10 × 10^−16^	4.73					
219478_at	WFDC1	WAP four-disulfide core domain 1	2.78 × 10^−15^	35.49					
205792_at	WISP2	WNT1 inducible signaling pathway protein 2	3.53 × 10^−4^	2.90					

## References

[1] Smith L.T., Holbrook K.A. (1982). Development of dermal connective tissue in human embryonic and fetal skin. Scan Electron Microsc..

[2] Smith L.T., Holbrook K.A., Byers P.H. (1982). Structure of the dermal matrix during development and in the adult. J. Investig. Dermatol..

[3] Haydont V., Bernard B.A., Fortunel N.O. (2019). Age-related evolutions of the dermis: Clinical signs, fibroblast and extracellular matrix dynamics. Mech. Ageing Dev..

[4] Harper R.A., Grove G. (1979). Human skin fibroblasts derived from papillary and reticular dermis: Differences in growth potential in vitro. Science.

[5] Mine S., Fortunel N.O., Pageon H., Asselineau D. (2008). Aging alters functionally human dermal papillary fibroblasts but not reticular fibroblasts: A new view of skin morphogenesis and aging. PLoS ONE.

[6] Janson D.G., Saintigny G., van Adrichem A., Mahé C., El Ghalbzouri A. (2012). Different gene expression patterns in human papillary and reticular fibroblasts. J. Investig. Dermatol..

[7] Nauroy P., Barruche V., Marchand L., Nindorera-Badara S., Bordes S., Closs B., Ruggiero F. (2017). Human Dermal Fibroblast Subpopulations Display Distinct Gene Signatures Related to Cell Behaviors and Matrisome. J. Investig. Dermatol..

[8] Haydont V., Neiveyans V., Zucchi H., Fortunel N.O., Asselineau D. (2019). Genome-wide profiling of adult human papillary and reticular fibroblasts identifies ACAN, Col XI α1, and PSG1 as general biomarkers of dermis ageing, and KANK4 as an exemplary effector of papillary fibroblast ageing, related to contractility. Mech. Ageing Dev..

[9] Haydont V., Neiveyans V., Fortunel N.O., Asselineau D. (2019). Transcriptome profiling of human papillary and reticular fibroblasts from adult interfollicular dermis pinpoints the ‘tissue skeleton’ gene network as a component of skin chrono-ageing. Mech. Ageing Dev..

[10] Breathnach A.S. (1978). Development and differentiation of dermal cells in man. J. Investig. Dermatol..

[11] Mills S.J., Cowin A.J., Kaur P. (2013). Pericytes, mesenchymal stem cells and the wound healing process. Cells.

[12] Zhuang L., Lawlor K.T., Schlueter H., Pieterse Z., Yu Y., Kaur P. (2018). Pericytes promote skin regeneration by inducing epidermal cell polarity and planar cell divisions. Life Sci. Alliance.

[13] Rusu M.C., Mirancea N., Mănoiu V.S., Vâlcu M., Nicolescu M.I., Păduraru D. (2012). Skin telocytes. Ann. Anat..

[14] Ceafalan L., Gherghiceanu M., Popescu L.M., Simionescu O. (2012). Telocytes in human skin--are they involved in skin regeneration?. J. Cell Mol. Med..

[15] Manole C.G., Gherghiceanu M., Simionescu O. (2015). Telocyte dynamics in psoriasis. J. Cell Mol. Med..

[16] Shoshkes-Carmel M., Wang Y.J., Wangensteen K.J., Tóth B., Kondo A., Massasa E.E., Itzkovitz S., Kaestner K.H. (2018). Subepithelial telocytes are an important source of Wnts that supports intestinal crypts. Nature.

[17] Gherghiceanu M., Popescu L.M. (2012). Cardiac telocytes—Their junctions and functional implications. Cell Tissue Res..

[18] Fertig E.T., Gherghiceanu M., Popescu L.M. (2014). Extracellular vesicles release by cardiac telocytes: Electron microscopy and electron tomography. J. Cell Mol. Med..

[19] Manole C.G., Simionescu O. (2016). The Cutaneous Telocytes. Adv. Exp. Med. Biol..

[20] Peltzer J., Montespan F., Thepenier C., Boutin L., Uzan G., Rouas-Freiss N., Lataillade J.J. (2015). Heterogeneous functions of perinatal mesenchymal stromal cells require a preselection before their banking for clinical use. Stem Cells Dev..

[21] Asselineau D., Bernhard B., Bailly C., Darmon M. (1985). Epidermal morphogenesis and induction of the 67 kD keratin polypeptide by culture of human keratinocytes at the liquid-air interface. Exp. Cell Res..

[22] Rheinwald J.G., Green H. (1975). Serial cultivation of strains of human epidermal keratinocytes: The formation of keratinizing colonies from single cells. Cell.

[23] Beacham D.A., Amatangelo M.D., Cukierman E. (2007). Preparation of extracellular matrices produced by cultured and primary fibroblasts. Curr. Protoc. Cell Biol..

[24] Gabbiani G. (1994). Modulation of fibroblastic cytoskeletal features during wound healing and fibrosis. Pathol. Res. Pract..

[25] Pei H., Yao Y., Yang Y., Liao K., Wu J.R. (2011). Krüppel-like factor KLF9 regulates PPARγ transactivation at the middle stage of adipogenesis. Cell Death Differ..

[26] Kudo A. (2011). Periostin in fibrillogenesis for tissue regeneration: Periostin actions inside and outside the cell. Cell Mol. Life Sci..

[27] Spicer A.P., Joo A., Bowling R.A. (2003). A hyaluronan binding link protein gene family whose members are physically linked adjacent to chondroitin sulfate proteoglycan core protein genes: The missing links. J. Biol. Chem..

[28] Kiani C., Chen L., Wu Y.J., Yee A.J., Yang B.B. (2002). Structure and function of aggrecan. Cell Res..

[29] Kadler K.E., Hill A., Canty-Laird E.G. (2008). Collagen fibrillogenesis: Fibronectin, integrins, and minor collagens as organizers and nucleators. Curr. Opin. Cell Biol..

[30] Kielty C.M., Sherratt M.J., Shuttleworth C.A. (2002). Elastic fibres. J. Cell Sci..

[31] Driskell R.R., Lichtenberger B.M., Hoste E., Kretzschmar K., Simons B.D., Charalambous M., Ferron S.R., Herault Y., Pavlovic G., Ferguson-Smith A.C. (2013). Distinct fibroblast lineages determine dermal architecture in skin development and repair. Nature.

[32] Salzer M.C., Lafzi A., Berenguer-Llergo A., Youssif C., Castellanos A., Solanas G., Peixoto F.O., Stephan-Otto Attolini C., Prats N., Aguilera M. (2018). Identity Noise and Adipogenic Traits Characterize Dermal Fibroblast Aging. Cell.

[33] Korosec A., Frech S., Gesslbauer B., Vierhapper M., Radtke C., Petzelbauer P., Lichtenberger B.M. (2019). Lineage Identity and Location within the Dermis Determine the Function of Papillary and Reticular Fibroblasts in Human Skin. J. Investig. Dermatol..

[34] Philippeos C., Telerman S.B., Oulès B., Pisco A.O., Shaw T.J., Elgueta R., Lombardi G., Driskell R.R., Soldin M., Lynch M.D. (2018). Spatial and Single-Cell Transcriptional Profiling Identifies Functionally Distinct Human Dermal Fibroblast Subpopulations. J. Investig. Dermatol..

[35] Brun C., Ly Ka So S., Maginiot F., Bensussan A., Michel L., Larghero J., Wong H., Oddos T., Cras A. (2016). Intrinsically aged dermal fibroblasts fail to differentiate into adipogenic lineage. Exp. Dermatol..

[36] Solé-Boldo L., Raddatz G., Schütz S., Mallm J.P., Rippe K., Lonsdorf A.S., Rodríguez-Paredes M., Lyko F. (2019). Single-cell transcriptomes of the aging human skin reveal loss of fibroblast priming. BioRxiv..

[37] Wollina U., Wetzker R., Abdel-Naser M.B., Kruglikov I.L. (2017). Role of adipose tissue in facial aging. Clin. Interv. Aging.

[38] Marsh E., Gonzalez D.G., Lathrop E.A., Boucher J., Greco V. (2018). Positional Stability and Membrane Occupancy Define Skin Fibroblast Homeostasis In Vivo. Cell.

[39] Varani J., Dame M.K., Rittie L., Fligiel S.E., Kang S., Fisher G.J., Voorhees J.J. (2006). Decreased collagen production in chronologically aged skin: Roles of age-dependent alteration in fibroblast function and defective mechanical stimulation. Am. J. Pathol..

